# Evaluation of 15 years of modeled atmospheric oxidized nitrogen compounds across the contiguous United States

**DOI:** 10.1525/elementa.2020.00158

**Published:** 2021-05-07

**Authors:** Claudia Toro, Kristen Foley, Heather Simon, Barron Henderson, Kirk R. Baker, Alison Eyth, Brian Timin, Wyat Appel, Deborah Luecken, Megan Beardsley, Darrell Sonntag, Norm Possiel, Sarah Roberts

**Affiliations:** 1 U.S. Environmental Protection Agency, Ann Arbor, MI, USA; 2 U.S. Environmental Protection Agency, Research Triangle Park, NC, USA

**Keywords:** CMAQ, NO_*X*_, NO_*Y*_, O_3_, Mobile emissions

## Abstract

Atmospheric nitrogen oxide and nitrogen dioxide (NO + NO_2_, together termed as NO_*X*_) estimates from annual photochemical simulations for years 2002–2016 are compared to surface network measurements of NO_*X*_ and total gas-phase-oxidized reactive nitrogen (NO_*Y*_) to evaluate the Community Multiscale Air Quality (CMAQ) modeling system performance by U.S. region, season, and time of day. In addition, aircraft measurements from 2011 Deriving Information on Surface Conditions from Column and Vertically Resolved Observations Relevant to Air Quality are used to evaluate how emissions, chemical mechanism, and measurement uncertainty each contribute to the overall model performance. We show distinct seasonal and time-of-day patterns in NO_*X*_ performance. Summertime NO_*X*_ is overpredicted with bimodal peaks in bias during early morning and evening hours and persisting overnight. The summertime morning NO_*X*_ bias dropped from between 28% and 57% for earlier years (2002–2012) to between −2% and 7% for later years (2013–2016). Summer daytime NO_*X*_ tends to be unbiased or underpredicted. In winter, the evening NO_*X*_ overpredictions remain, but NO_*X*_ is unbiased or underpredicted overnight, in the morning, and during the day. NO_*X*_ overpredictions are most pronounced in the Midwestern and Southern United States with Western regions having more of a tendency toward model underpredictions of NO_*X*_. Modeled NO_*X*_ performance has improved substantially over time, reflecting updates to the emission inputs and the CMAQ air quality model. Model performance improvements are largest for years simulated with CMAQv5.1 or later and for emission inventory years 2014 and later, coinciding with reduced onroad NO_*X*_ emissions from vehicles with newer emission control technologies and improved treatment of chemistry, deposition, and vertical mixing in CMAQ. Our findings suggest that emissions temporalization of specific mobile source sectors have a small impact on model performance, while chemistry updates improve predictions of NO_*Y*_ but do not improve summertime NO_*X*_ bias in the Baltimore/DC area. Sensitivity runs performed for different locations across the country suggest that the improvement in summer NO_*X*_ performance can be attributed to updates in vertical mixing incorporated in CMAQv5.1.

## Introduction

Exposure to nitrogen oxides (NO_*X*_ = NO + NO_2_) can lead to the development of asthma and asthma exacerbation and has been associated with cardiovascular effects, diabetes, cancer, and premature mortality (e.g., U.S. Environmental Protection Agency [EPA], 2016a). In addition, NO_*X*_ is a precursor to other detrimental air pollutants such as ozone (O_3_) and particulate matter less than 2.5 micrometers in diameter (PM_2.5_; Seinfeld and Pandis, 1997). Consequently, understanding NO_*X*_ sources, chemistry, and deposition contributes to sound air quality management decisions. Emission inventories that quantify NO_*X*_ emissions from different sources are often used as input to photochemical models which simulate the atmospheric fate and transport of NO_*X*_ and its reaction products (HNO_3_, N_2_O_5_, HONO, HO_2_NO_2_, ClNO_2_, peroxyl acetyl nitrate [PAN], NO_3_, and organic nitrates), together referred to as total gas-phase-oxidized reactive nitrogen or NO_*Y*_. Evaluation of NO_*X*_ emissions inventories, as well as photochemical model treatment of physical and chemical processes which impact atmospheric NO_*Y*_ budgets, is essential for building confidence in the tools that inform regulatory decisions.

Evaluation of the National Emission Inventory (NEI) is challenging due to the large number of sources contributing to NO_*X*_ emissions in the United States. Emissions of NO_*X*_ originate from many different types of source categories, and the emission contributions vary both between and within sectors. Furthermore, variations in emissions timing (diurnal and seasonal), location, and technology are important for interpreting model performance and making appropriate choices for emissions control programs.

**Figure 1.**
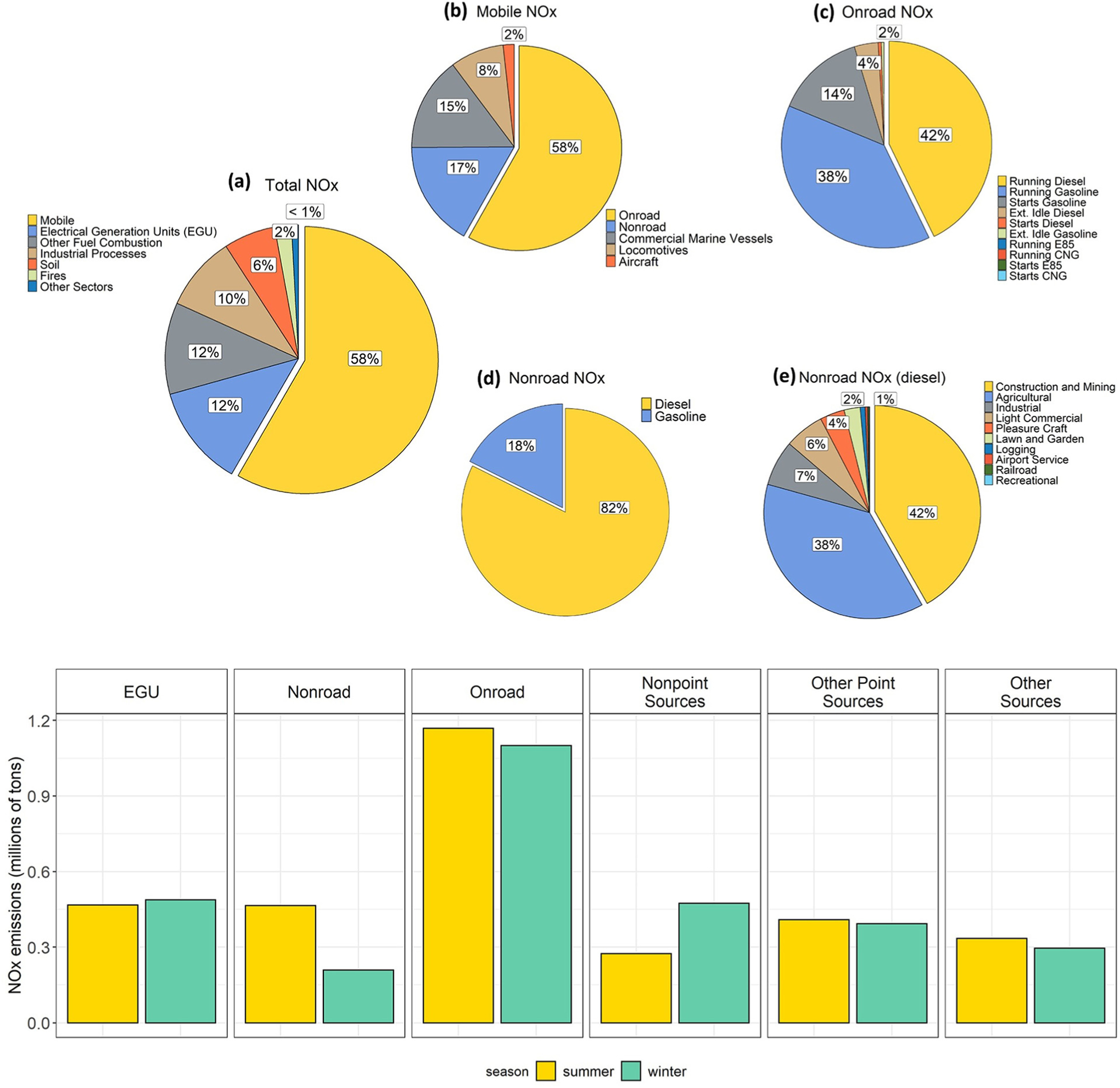
National Emission Inventory of nitrogen oxides for 2014. Top panel shows pie charts with the proportion of each sector for (a) all sources, (b) mobile, (c) onroad, (d) nonroad, and (e) nonroad diesel categories. Bottom panel shows seasonal emissions for the major sectors contributing to NO_*X*_ emissions in 2014. Summer represents June–July–August and Winter represents December–January–February. DOI: https://doi.org/10.1525/elementa.2020.00158.f1

Nationally, the majority of NO_*X*_ emissions come from mobile sources that are split in the NEI into different categories (see Figure 1). Within the onroad category, running emissions are defined as emissions resulting from vehicle operations on the road either under load or idling. NO_*X*_ emissions that occur when the engine catalyst is not fully warmed up during the start-up of the vehicle are particularly important in gasoline vehicles. Emissions associated with extended idling operation result from the activity of heavy-duty trucks with the engine idling to power vehicle accessories (such as air conditioning, heat, television, etc.) during the mandatory rest period. These accessories generally require more engine power than regular idling on the road which is why the process is differentiated in the inventory.

Other large contributors to U.S. NO_*X*_ inventory include emissions from fuel combustion (boilers and internal combustion engines, power plants, and residential combustion of coal, oil, natural gas, and wood), industrial processes (oil and gas, manufacturing, and mining), nonroad equipment (including construction, mining, agricultural, and lawn and garden equipment), commercial marine vessels, and biogenic sources.

In addition to the spatial and temporal variability associated with emissions from different emission sectors, transport, chemistry, and deposition can impact observed and modeled NO_*X*_ mixing ratios. A variety of approaches have been used to assess NO_*X*_ emissions, including comparing emission ratios with ambient (surface and aircraft) or remotely sensed (satellite) information, fuel-based analysis, and the application of photochemical grid models using NEI data (U.S. EPA, 2015a; U.S. EPA, 2016b) as input.

Anderson et al. (2014) and Canty et al. (2015) compared NEI emission ratios with measurements from a July 2011 mid-Atlantic intensive field measurement campaign and concluded that NO_*X*_ emissions from mobile sources were overpredicted by a factor of 2. Simon et al. (2018) also compared 2011-based model predictions with the same field data and suggest that attribution of model performance to a specific sector is challenging using only emission ratios and that measurement uncertainty also confounds emissions evaluation. Fuel-based analysis of the mobile emission inventory for 2013 suggests that mobile source emissions of NO_*X*_ are overestimated by 28% (McDonald et al., 2018). Other researchers used measurements from various field campaigns that took place during the summer of 2013 in Texas (Souri et al., 2016) and across the Southeastern United States (Li et al., 2016; Travis et al., 2016) and suggested that NO_*X*_ emissions from anthropogenic sources other than power plants were overpredicted by between 30% and 60%. Appel et al. (2017) used NO_*X*_ measurements from ambient monitoring networks across the United States to evaluate modeled NO_*X*_ mixing ratios in 2011 and found model overpredictions of NO_*X*_ mixing ratios overnight and during the morning and evening hours during the summer but not during winter or during the middle of the day. Salmon et al. (2018) and Jaeglé et al. (2018) found reasonable agreement between model predictions based on an EPA emissions inventory and measured ambient NO_*X*_ in the mid-Atlantic and Northeast, respectively, during the winter of 2015.

Here, photochemical model predictions of NO_*X*_ for 15 annual simulations (2002–2016) were compared with routine surface network measurements to illustrate the modeling systems’ ability to replicate observed patterns across space, season, and time of day. NO_*X*_ emissions are presented from the 2014 NEI to provide broader context about the largest sectors. Because meteorology can affect evaluation results for a single year (e.g., years with cool wet summers can reveal different model biases compared to years with more hot, stagnant summer conditions), evaluation across multiple years provides the chance to identify systematic biases in the Community Multiscale Air Quality (CMAQ) estimates. In addition, during this 15-year time period, there have been improvements in the input data sets and parameterizations used by the meteorology, emissions, and air quality models applied in this study. Dynamic evaluation studies, such as this one, offer the chance to analyze the cumulative impacts of these improvements on NO_*X*_ performance. Diagnostic evaluation is also used to isolate and test specific hypotheses about the causes of model biases. Aircraft measurements (Crawford et al., 2014; Crawford and Pickering, 2014) were compared to model estimates to further explore how model formulation (e.g., chemistry) and measurement uncertainty contribute to bias in model estimates of NO_*X*_.

## Methods

The CMAQ model (U.S. EPA Office of Research and Development, 2014) was applied for multiple annual simulations covering 2002–2016. Meteorological inputs were developed using the Weather Research and Forecasting (WRF) model (Skamarock and Klemp, 2008). These simulations were developed over several years to support multiple projects, so model versions and assumptions about inputs vary. However, this collective data set has the advantage of demonstrating how the state-of-the-science models were performing at the time that EPA and others were modeling each historic year, as well as for the most recent years. The 2002–2012 CMAQ v5.0.2 simulations are described in Zhang et al. (2019). A complete list of annual simulations, model versions, and other key information is provided in Table 1. All simulations were performed for a model domain covering the contiguous United States using 12-km horizontal grid spacing. Lateral boundary inflow for chemical species was extracted from coarser scale hemispheric air quality model simulations (Henderson et al., 2014). Additional model sensitivity simulations were conducted to test several hypotheses on drivers of modeled NO_*X*_ bias for the period coincident with the 2011 Deriving Information on Surface Conditions from Column and Vertically Resolved Observations Relevant to Air Quality (DISCOVER-AQ) field study.

Air quality model-ready emissions were prepared using the Sparse Matrix Operator Kernel Emissions (SMOKE; Baek and Seppanen, 2021) modeling system. Biogenic emissions were estimated with the Biogenic Emission Inventory System (Pierce et al., 1998; Schwede et al., 2005). Wildland fire emissions were developed using SmartFire for fire timing and location and BlueSky Framework for fuels and emissions (Baker et al., 2016). NO from lightning production was calculated online in CMAQ using year-specific lightning strike data from the National Lightning Detection Network (Kang, 2019a, 2019b).

Year-specific information for anthropogenic emissions was used where possible for each model simulation. Electrical Generating Unit (EGU) emissions were based on hourly data submitted to the Continuous Emissions Monitor system. Other (non-EGU) anthropogenic point and area source emissions were based on EPA’s triennial NEI (U.S. EPA, 2016b). Onroad mobile emissions were estimated using the most current version of the Motor Vehicle Emission Simulator (MOVES; U.S. EPA, n.d.-b) available at the time of NEI development (see Table 1). Nonroad mobile emissions were developed using the most current version of the Nonroad component of MOVES model available at the time of NEI development. MOVES emissions of onroad and nonroad emissions for California were adjusted to the inventory estimates provided by that state. For years between NEIs, the sectors without specific data (such as EGUs) were interpolated from the nearest available NEI at the time the inventory was developed.

Meteorological input data were evaluated against observations from meteorological aerodrome reports (ME-TAR) available from the Meteorological Assimilation and Data Ingest System (National Oceanic and Atmospheric Administration [NOAA], n.d.) Model and observed meteorological data were paired in time and space using the Atmospheric Model Evaluation Tool version 1.4 (U.S. EPA, 2019).

Nitrogen oxides (NO_*X*_) monitor sites included in the analysis used a standard chemiluminescence instrument. Ambient data from national networks included in the EPA’s Air Quality System (AQS; U.S. EPA, n.d.-a) for hourly gas species were matched to model predictions in time and space based on the grid cell where the monitor was located. These comparisons were made for whichever set of monitors was operational on each day from 2002 to 2016, so different locations and numbers of monitors are included in comparisons for different years and seasons (see Figures S1, S3, and S7). Monitors in EPA’s near-road monitoring network were excluded from this analysis because 12-km resolution grid boxes are not expected to accurately capture near-road conditions. Mean bias (MB) and normalized mean bias (NMB) were calculated as described in Simon et al. (2012). Ambient NO_*X*_ monitors have known artifacts and often pick up additional NO_*Y*_ species in their measurements (Dunlea et al., 2007; Dickerson et al., 2019). This problem is most pronounced at times and locations with higher fractions of aged NO_*Y*_ species compared to NO_*X*_. To bound this measurement uncertainty, we compared measured NO_*X*_ both to modeled NO_*X*_ and to modeled NO_*Y*_. In addition, we focus analysis on morning hours (4–9 AM LST) when fresh NO_*X*_ emissions are expected to dominate NO_*Y*_, thus limiting the impact of measurement artifacts.

Oxidized nitrogen gas field measurements were made as part of the July 2011 DISCOVER-AQ field study over the Baltimore, Maryland, region (Crawford et al., 2014; Crawford and Pickering, 2014). This field study includes surface and upper air measurements of NO_*X*_ and NO_*Y*_, which provides greater spatial and temporal coverage of the metropolitan area than available from routine ground-based monitors. In this analysis, NO_*Y*_ is examined to more fully characterize oxidized nitrogen gases to reduce model and measurement incommensurability solely related to the operational definition of NO_*X*_. Aircraft measurements of oxidized nitrogen gases from DISCOVER-AQ were matched to the model predictions in time and space (Simon et al., 2018). Specifically, hourly 3D gridded model mixing ratios were matched to 5-min aircraft measurements using the grid-cell center closest to the aircraft latitude, longitude, and altitude and the modeled mixing ratio at the top-of-the-hour closest to the measurement time. Model species that were mapped to each measured species are listed in Table S1.

## Results and discussion

### Emission inventory characterization

**Figure 2.**
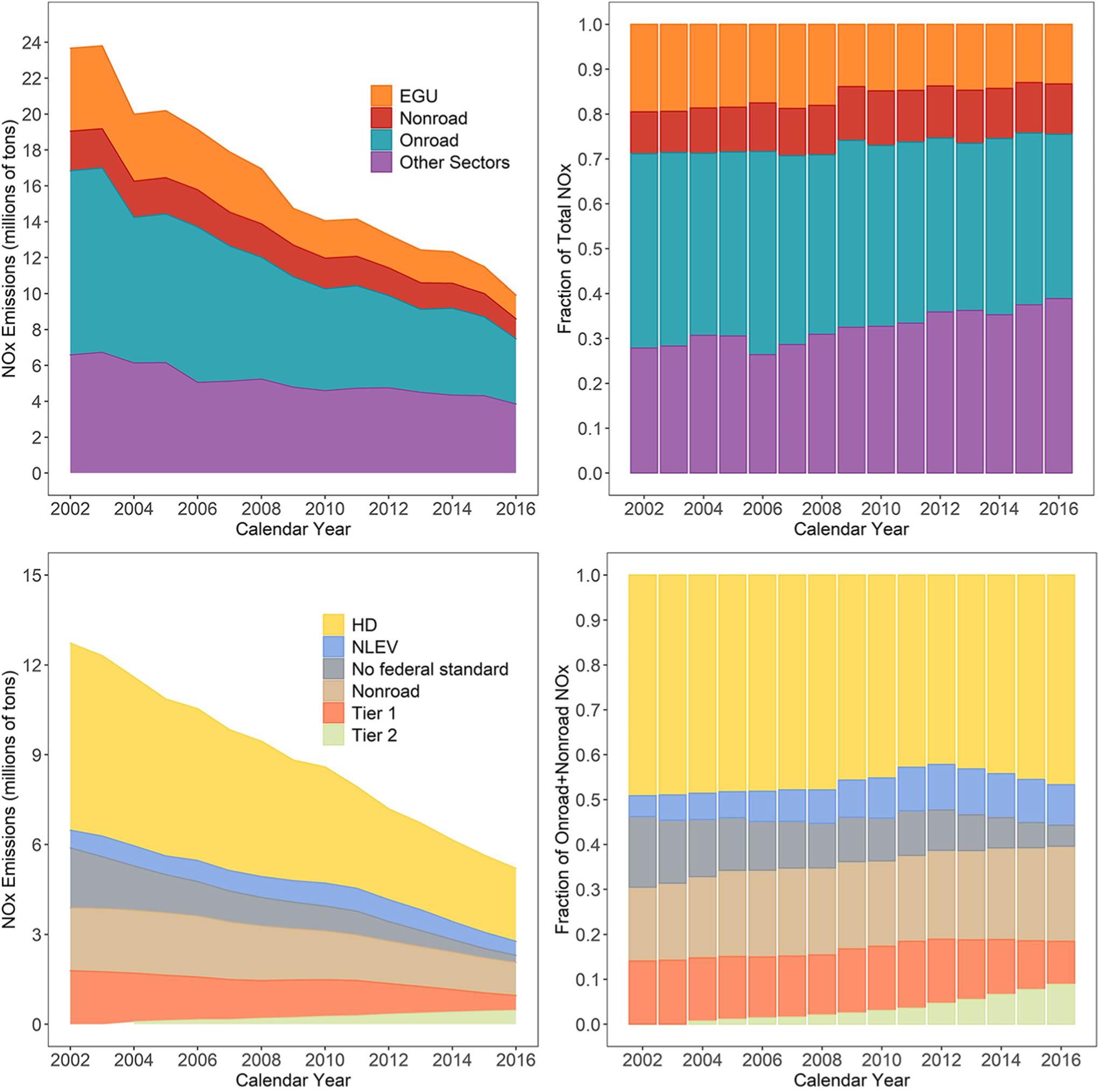
National trend between 2002 and 2016 for total NO_*X*_ emissions (top row) and for onroad and nonroad vehicles (bottom row). Trends are shown as absolute emissions (left column) and as fractional contribution to total NO_*X*_ emissions (top right) and to onroad + nonroad NO_*X*_ emissions (bottom right). Bottom row figures were estimated using MOVES2014b. The light-duty sector (passenger cars, passenger trucks, and commercial trucks) is divided to represent the contribution of vehicles manufactured under specific emission standards (Tier 1 = model years between 1994 and 2000; National Low Emissions Vehicle = model years 2000–2003; Tier 2 = model years after 2004). DOI: https://doi.org/10.1525/elementa.2020.00158.f2

Figure 1 provides an annual and seasonal breakout of NO_*X*_ in the 2014 NEI by sector and further breaks out subsectors for mobile sources. Sector definitions are available in the 2014 NEI technical support document (U.S. EPA, 2018). The national trend between 2002 and 2016 for NO_*X*_ emissions from onroad and off-road vehicles estimated using MOVES2014b consistently for all years is shown in Figure 2. Note that this trend is different than the emissions used in the CMAQ runs, as shown in Table 1, since each of the CMAQ simulations used different emissions model versions, local inputs, and activity. Nationally, onroad and off-road emissions have decreased significantly each year, and these differences vary among vehicles/equipment with different emission control technologies, as older vehicles/equipment are phased out from the fleet and as vehicles/equipment meeting more stringent standards come into the fleet. Nonetheless, it should be noted that relative contributions from different mobile source sectors will vary spatially within the United States. Past studies have pointed toward onroad vehicles as a major source of NO_*X*_ emissions overpredictions (Anderson et al., 2014; Travis et al., 2016). Figure 1 shows that while mobile sources account for over half of U.S. NO_*X*_ emissions and onroad vehicles contribute over half of U.S. mobile source emissions, onroad vehicles only contribute about one-third of total U.S. NO_X_ emissions with roughly equal parts coming from gasoline and diesel vehicles for the calendar year 2014.

NO_*X*_ emissions can vary by season (Figure 1, bottom panel) due to environmental reasons (e.g., wildfires) or due to changes in anthropogenic activities. Total NO_*X*_ emissions vary by approximately 15% between summer (June–July–August) and winter (December–January–February), with summer emissions generally higher due to seasonal activity. For example, EGU usage varies depending on electricity demand, which is generally highest in summer. This peak demand generally results in 14%–16% increase in NO_*X*_ emissions for this sector during the summer season. However, 2014 had a particularly cold winter and the 2014 NEI reflects this by estimating less than 5% change in NO_*X*_ emissions between summer and winter (Figure 1). Nonroad equipment usage also changes through the year with sectors like Construction and Lawn & Garden significantly increasing during summer months, resulting in summer emissions estimates that are more than twice the winter NO_*X*_ emissions estimates for 2014. Onroad emissions do not show large differences in activity between seasons, but emissions of NO_*X*_ from gasoline vehicles are influenced by the increase in engine load caused by A/C usage during the summer season. On a national scale, the seasonal difference in total onroad emissions is small (approximately 6% higher in summer). Nonetheless, it is important to highlight that temperature adjustments of onroad gasoline running emissions are dependent on local conditions. EPA’s SMOKE-MOVES modeling system predicts that absolute NO_*X*_ gasoline running emissions increase slightly during summer afternoon hours in some locations compared to winter months. The change in light-duty running emissions estimated in summer is a balance between the impact of humidity on combustion temperature, which effectively reduces NO_*X*_ emissions, and the increase in engine load due to the use of air conditioning which increases NO_*X*_ emissions (U.S. EPA, 2015c). The net result depends on the meteorological conditions of each location. Ambient temperature also has an impact on start emissions. Colder temperatures impact the time that the catalyst requires to reach a functional temperature which results in higher NO_*X*_ start emissions during winter season (U.S. EPA, 2015c), but as noted in Figure 1, these emissions are a small part of the total NO_*X*_ inventory and more relevant at a local scale. Ambient temperature has not been shown to impact running emissions significantly in laboratory tests as the exhaust air temperature is on the order of 300 °C (U.S. EPA, 2015c). Nonetheless, seasonal variability of onroad emissions due to ambient temperature, particularly from heavy-duty trucks, has been suggested as a reason to explain a disagreement of a factor of 2 between comparisons of near-road NO_*X*_ measurements and MOVES2014 (Hall et al., 2020) and aircraft measurements with the onroad sector of the 2011 NEI during summer season (Salmon et al., 2018).

### Modeled spatial patterns of NO_*X*_, NO_*Y*_, and NO_*Z*_

**Figure 3.**
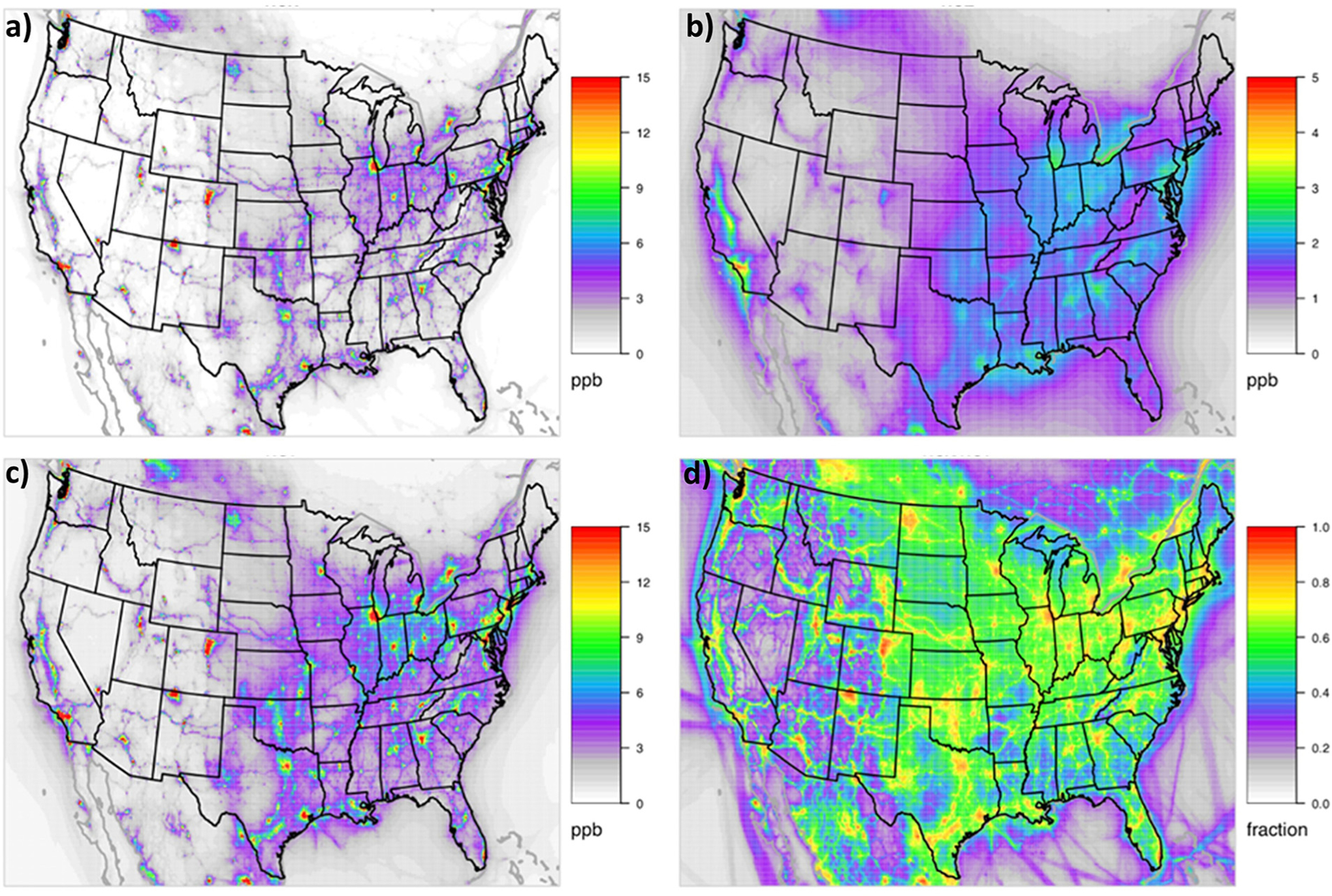
Modeled annual average of oxidized nitrogen species for 2015. (a) NO_*X*_ (NO+ NO_2_), (b) NO_*Z*_ (the difference between NO_*Y*_ and NO_*X*_), (c) NO_*Y*_ (all oxidized species), and (d) the ratio of NO_*X*_ to NO_*Y*_. Spatial maps for winter and summer are shown in Supplemental Material (Figure S2). DOI: https://doi.org/10.1525/elementa.2020.00158.f3

Modeled annual average NO_*X*_ is highest in urban areas and near large industrial sources (Figure 3). Major transportation corridors are a large source of NO_*X*_ in both urban and rural areas (Figure 3). Annual average NO_*Y*_ has a very similar spatial pattern as NO_*X*_ but is always slightly higher since NO_*X*_ is a subset of NO_*Y*_. Oxide nitrogen species other than NO_*X*_ (NO_*Z*_) comprise the difference between NO_*Y*_ and NO_*X*_. NO_*Z*_ species come from atmospheric aging of NO_*X*_ and tend to be more regionally homogeneous than NO_*X*_ but still highest in places with high NO_*X*_ emissions (e.g., urban areas, large transportation corridors). Major NO_*Z*_ components include nitric acid which has a short atmospheric lifetime due to a high dry deposition velocity and alkyl nitrates (ANs) and PANs which can travel farther downwind and contribute to O_3_ production days after formation in NO_*X*_-limited areas. In many urban areas of the United States, where NO_*X*_ mixing ratios tend to be the highest, NO_*X*_ comprises more than 90% of NO_*Y*_ annually on average (Figure 3). Figure S2, which maps NO_*X*_ and NO_*Y*_ mixing ratios in summer and winter, shows that modeled NO_*X*_ mixing ratios are larger in the winter while modeled NO_*Z*_ mixing ratios are larger in the summer. As a result, the NO_*X*_/NO_*Y*_ fraction is substantially larger in the winter than in the summer. In the summer, NO_*X*_ often makes up less than 80% of the NO_*Y*_ in urban core areas, around 70% of NO_*Y*_ near large transportation corridors, and between 40% and 60% of NO_*Y*_ regionally across the Eastern United States. In contrast, wintertime NO_*X*_ makes up over 90% of NO_*Y*_ in urban core areas and between 60% and 80% of NO_*Y*_ regionally across the Eastern United States. As a consequence, NO_*X*_ measurement artifacts are expected to add more uncertainty to model bias calculations in the summer and outside of urban core areas.

### 2002–2016 model performance

**Figure 4.**
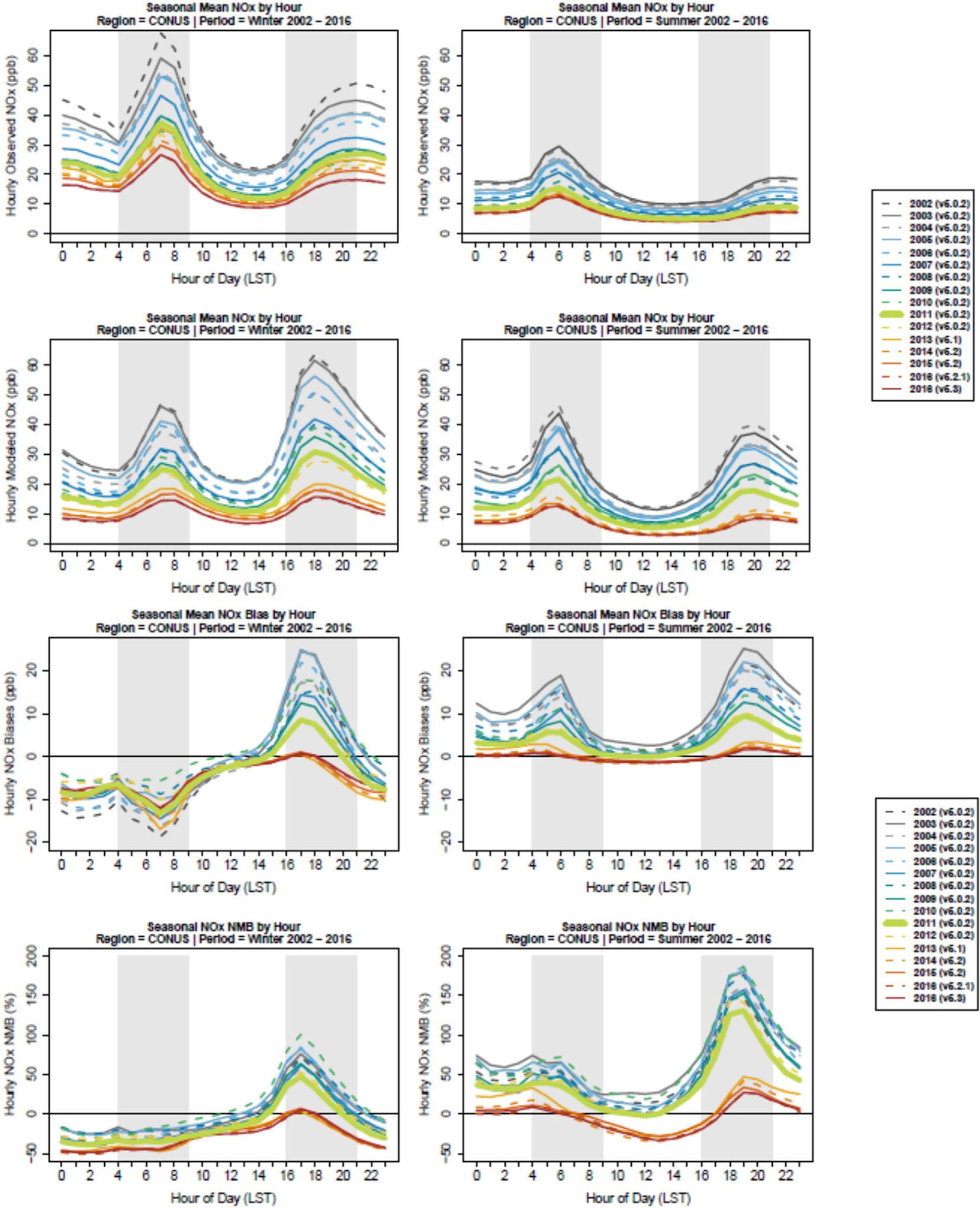
Diurnal mean NO_*X*_ observations (top) and bias (bottom). Profiles for winter (left) and summer (right) are shown by year. Modeled years include 2002–2016. DOI: https://doi.org/10.1525/elementa.2020.00158.f4

The diurnal pattern of mean NO_*X*_ mixing ratio and NO_*X*_ bias for monitors in the United States for the summer and winter season is shown in Figure 4 for each annual simulation. During summer months, there is a distinct morning and evening peak in MB from 4 to 9 AM and 4 to 9 PM, respectively, while at midday hours, the model underestimates NO_*X*_ mixing ratios. The morning biases track the hourly observations well, while the timing of the peak in the evening biases occurs during the rise in evening NO_*X*_ and subsides when the evening NO_*X*_ peaks. These two different behaviors are the result of competing effects of emissions versus planetary boundary layer (PBL) depth which may coincide temporally more closely in the morning than in the evening. We explore the interplay of these two factors in more depth in the following section. During winter months, the morning peak in bias observed in summer is absent while the evening peak shifts to earlier hours. The morning and evening periods of model overestimation (MB and NMB) of NO_*X*_ have been reduced between the 2002 and 2016 simulations. As discussed in more detail below and shown in Table 1, several shifts in the modeling platform may contribute to the year-to-year trends in NO_*X*_ bias shown in Figure 4 including a change in CMAQ model version to CMAQv5.1 in the 2013 simulation and a change in the underlying NEI version in the 2014 simulations and later. Nationally, summertime morning NO_*X*_ bias (4–9 AM) was between 28% and 57% from 2002 to 2012 (Figure S4). This bias dropped to 7% in 2013 and further to between −2% and 2% in the 2014–2016 time period. Similar improvements in summertime evening overpredictions are also shown in Figure 4. However, modest daytime (10 AM–3 PM) overpredictions in 2012 and prior (5%–34%) become modest underpredictions in 2013 and later simulations (−24% to −32%; see Figure S5). During the winter of these years, the model tends to underpredict the morning commute and midday hours and is less biased during the evening commute. The wintertime morning NO_*X*_ underpredictions increased from a bias between −17% and −33% in 2002–2012 simulations to a bias between −41% and −43% in 2013–2016 simulations (Figure S4). In contrast, wintertime evening NO_*X*_ overpredictions shifted from between 92% and 142% in 2002–2012 simulations to 12%–27% in 2013–2016 simulations (Figure S6).

**Figure 5.**
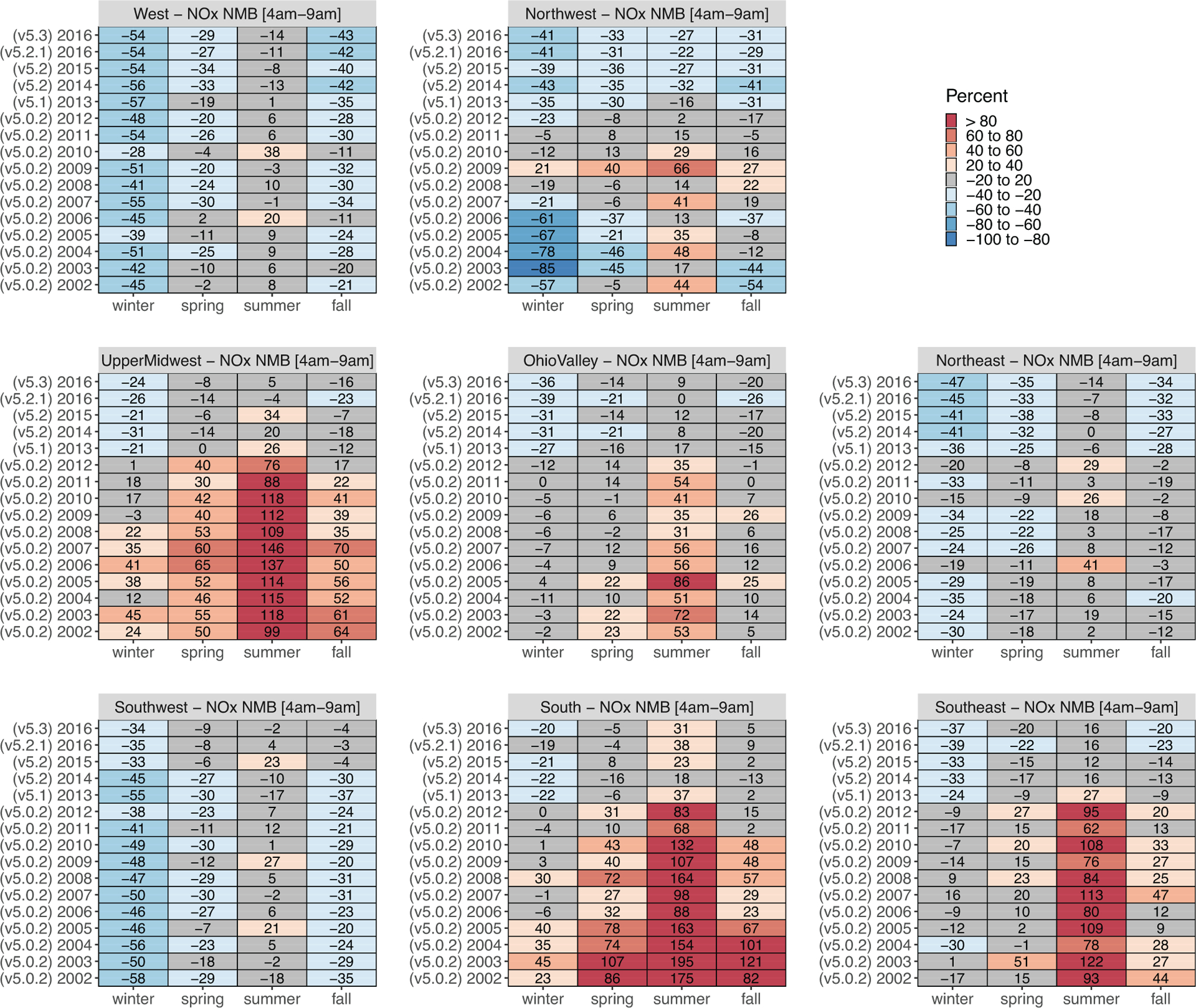
Normalized mean bias of morning modeled NO_*X*_ – observed NO_*X*_. Morning hours are 4–9 AM LST. Morning bias is aggregated by season for each annual simulation across monitors in multiple regions defined by National Oceanic and Atmospheric Administration climate region (Figure S1). Community Multiscale Air Quality model version used for each simulated year is shown in parentheses. Reds indicate model overprediction, and blues show model underprediction. West = CA and NV; Northwest (combined with the Northern Rockies and Plains) = OR, WA, ID, MT, NE, ND, SD, and WY; Upper Midwest = IA, MI, MN, and WI; Ohio Valley = IL, IN, KY, MO, OH, TN, and WV; Northeast = CT, DE, ME, MD, MA, NH, MJ, NY, PA, RI, and VT; Southwest = AZ, CO, NM, and UT; South = AR, KS, LA, MS, OK, and TX; and Southeast = AL, FL, GA, NC, SC, and VA. DOI: https://doi.org/10.1525/elementa.2020.00158.f5

NMB based on hourly NO_*X*_ data from 4 to 9 AM LST aggregated by season and region for each annual simulation is shown in Figure 5.The number of monitoring sites used to calculate NMB for each region/season/year combination is provided in Figure S7, and other model performance metrics (normalized mean error and correlation) are shown in Figures S12–S14. Performance features vary by region of the United States, season, and modeled year. The modeling system tended to overpredict NO_*X*_ during the summer, particularly in the Midwest and Southern United States.

The summertime NMB decreased substantially across the country beginning with the 2013 simulation with the switch to more recent CMAQ versions. Several major updates were made to science processes in the CMAQ model starting in v5.1 including changes in the treatment of vertical mixing in urban areas that resulted in more mixing and less pollutant buildup near the surface, an update of the underlying chemical mechanism from Carbon Bond 5 (CB05) to CB05e51, improved calculations of photolysis with a more detailed treatment of cloud and aerosol interactions, and updates to the dry deposition scheme (Appel et al., 2017). There was an additional incremental improvement in the NMB from 2013 to 2014–2016 simulations since they were simulated using an even more recent version of CMAQ (v5.2) and included emissions based on different NEI inventory—2011 NEI for 2013 simulation and 2014 NEI with improved methodology and data for the 2014–2016 simulations.

The version of WRF that was used to provide meteorological inputs also changed across the simulations. WRF version 3.4 was used for the 2002–2012 simulations, version 3.7.1 for 2013, and version 3.8 for 2014–2016. Evaluation of estimated temperature, windspeed, and mixing ratio against METAR observations shows the MB in these variables tends to be low and fairly stable across the 16 years for all regions (e.g., less than ±0.5 °C temperature bias; ≤1 m/s windspeed bias, see Figures S8–S11). This is to be expected since the WRF simulations used data assimilation to adjust model values toward surface and upper-level meteorological data. Even with the use of data assimilation for all simulation years, meteorological updates can still impact NO_*X*_ mixing ratios, as discussed further in the next section.

Another potential contributing factor to the reduced NMB is that NO_*X*_ emissions from various types of sources have decreased at different rates (see Figure 2), so any emissions bias that resulted from a subset of sources may become less pronounced if that group of sources makes up a smaller portion of the total NO_*X*_ emissions. Figure 2 shows annual trends in total and relative NO_*X*_ emissions magnitudes from different source sectors and further breaks out for onroad vehicles and off-road equipment. While onroad and nonroad NO_*X*_ emissions have continuously decreased throughout the entire time series (Figure 2), older gasoline vehicles with higher NO_*X*_ emissions (manufactured in years without federal NO_*X*_ emissions standards) contribute less to onroad NO_*X*_ steadily in later years as newer gasoline vehicles meeting more stringent NO_*X*_ emissions standards (Tier 2) have become more prevalent. For instance, vehicles without any federal NO_*X*_ emissions standards made up 16% of the total onroad and nonroad NO_*X*_ emissions in 2002 and only 5% in 2016. Even though their share of the total on- and nonroad NO_*X*_ emissions is not the largest, changes in gasoline vehicle NO_*X*_ contribution are relevant to the morning and evening overprediction since these are the times of the day when their activity is highest in urban areas. While all of these factors likely contribute to a large reduction in NMB beginning with the 2013 simulation, the correlation between modeled and observed NO_*X*_ was not similarly improved (Figure S13). *R*^2^ values were below 0.6 for all seasons, even in the most recent simulation years with values in the range of 0.3 for most regions, seasons, and simulation years. The correlation values reflect the model’s ability to capture both spatial and temporal (daily) variability. The temporal variability is impacted both by emissions timing and meteorology. Past studies have shown CMAQ generally does a reasonable job of capturing day-to-day variability for other primary pollutants (Appel et al., 2012; U.S. EPA, 2012), so it is likely that comparatively low *R*^2^ values are due to the model’s inability to fully capture the spatial variability of NO_*X*_ which may have steep gradients in urban areas near sources. It may not be possible to capture these NO_*X*_ gradients in urban areas with 12-km resolution modeling.

In contrast to the summer overestimation, the modeling system tended to underestimate NO_*X*_ in the winter. The underestimation occurred mainly in the three western regions and the northeast, but all eight regions had negative NMB values for the most recent 2013–2016 simulations. Maps of summer and winter season MB for all simulations are provided in Figure S15 to illustrate regional and within-region differences in NO_*X*_ performance. For the most recent model simulation (2016 using CMAQv5.3), the winter season underpredictions occur throughout the United States and most notably in large urban areas. Modeled NO_*X*_ summer overestimation persists in this simulation in states along the Gulf of Mexico, while large metropolitan areas such as New York and Los Angeles have monitors with both over and underprediction of NO_*X*_ (Figure S15).

In addition to the model performance for NO_*X*_ shown in Figure 5, we investigated the impact of measurement uncertainty from routine network NO_*X*_ monitors on model performance by comparing modeled NO_*Y*_ to measured NO_*X*_ as recommended by Dickerson et al. (2019). The evaluation in Figure S14 treats the AQS measurements as capturing all NO_*Y*_ species rather than NO_*X*_. This comparison shows generally larger modeled NO_*Y*_ overpredictions (as expected since monitors are unlikely to capture total NO_*Y*_ mixing ratios) but with the same temporal trends and spatial patterns as were shown in Figure 5.

### Diagnostic evaluation using 2011 model sensitivity simulations and evaluation against field data

Multiple processes could be driving the seasonal differences in biases seen in Figures 4 and 5. Due to the available modeling simulations that represent the evolution of state-of-the-science modeling over time, it is difficult to isolate causes of NO_*X*_ bias and the main drivers for changing model performance over this set of simulations. Major aspects of the modeling system which may contribute to NO_*X*_ bias include modeled temperature dependence of mobile emissions in MOVES or other NO_*X*_ sources (e.g., soil NO), the temporal allocation of emissions used to convert annual or daily total emissions to hourly estimates, the parameterization of vertical mixing in WRF and CMAQ, and the seasonal differences in the impact of chemistry and deposition on NO_*X*_ atmospheric lifetime. In this section, we use additional detailed model and measurement data to explore the potential impacts of these various phenomena for the 2011 modeling year.

Figures 4 and 5 showed some distinct temporal patterns in NO_*X*_ bias including higher bias at night and during morning/evening rush hour and a tendency for the model to overpredict NO_*X*_ mixing ratios in the summer and underpredict NO_*X*_ mixing ratios in the winter. In order to test the impact of emissions timing on these bias patterns, we first identified several emissions sectors with large contributions to total NO_*X*_ emissions (Figure 2) and uncertain or potentially inaccurate temporal profiles. These included heavy-duty onroad vehicles, nonroad equipment, and the subset of EGUs without continuous emissions monitoring systems. We conducted three sensitivity simulations for 2011 in which we updated the timing of emissions from each of these sectors using the best current information which is described in detail in the supplemental information. These updates often resulted in more emissions being allocated to times of day and year with higher PBL heights and more mixing and fewer emissions being allocated to times of day and year with lower PBL heights and less vertical mixing. As a result of these updates, NO_*X*_ mixing ratios across the United States decreased generally by less than 1 ppb on average (Figure S19) although decreases were more pronounced at specific times and locations. The results of these sensitivity simulations indicate that the emissions timing updates did not systematically change modeled NO_*X*_ mixing ratios in a way that could explain the modeled NO_*X*_ bias.

**Figure 6.**
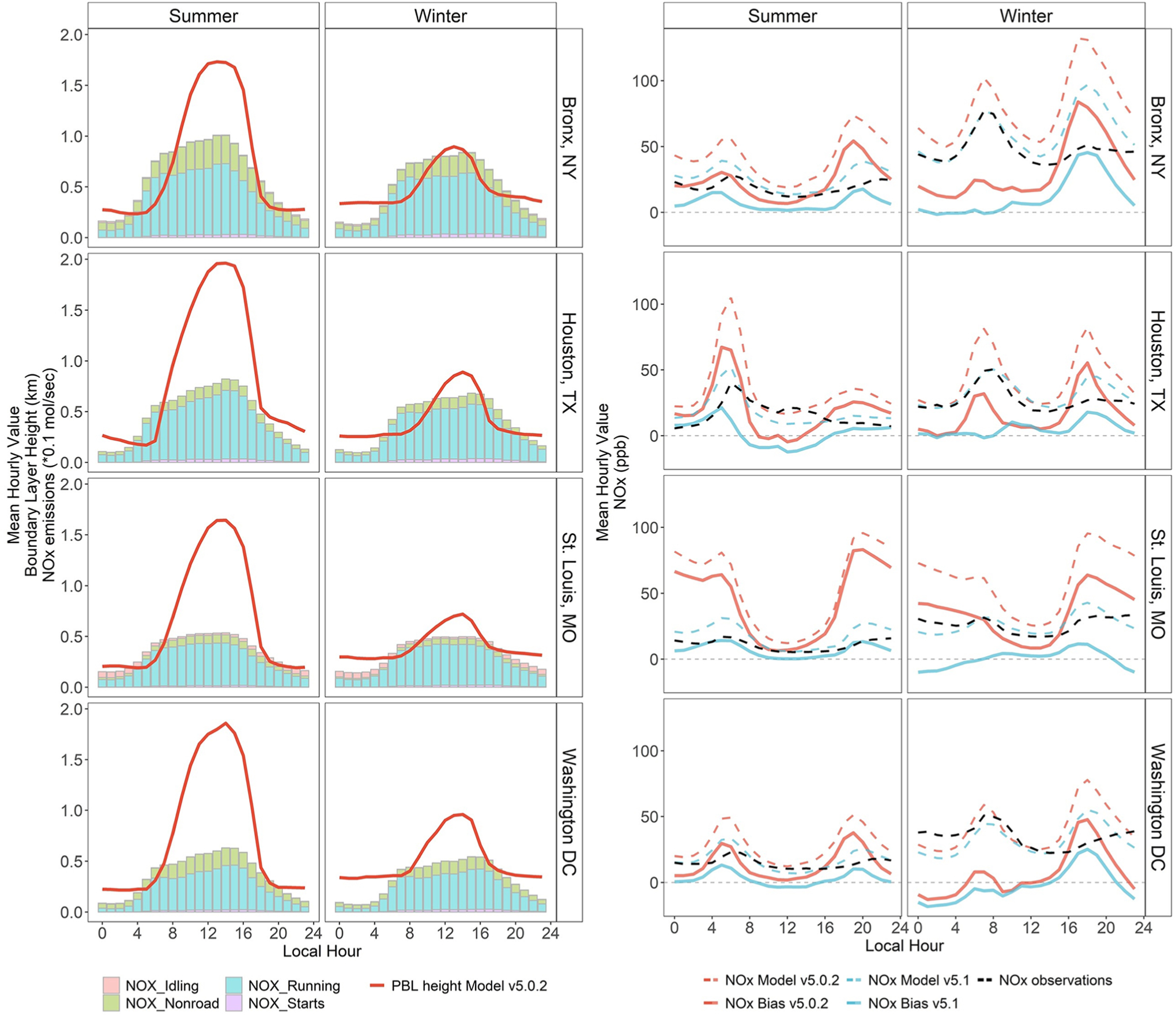
Seasonal differences in diurnal profiles of NO_*X*_ bias, mobile NO_*X*_ emissions, and modeled boundary layer. Summer and winter diurnal profiles for modeled average boundary layer height, NO_*X*_ onroad + nonroad emissions (left panel), NO_*X*_ observations, NO_*X*_ modeled mixing ratios, and calculated bias (right panel). NO_*X*_ bias and mixing ratios are shown for two Community Multiscale Air Quality (CMAQ) versions (v5.02 and v5.1) for multiple urban areas in 2011. Emissions are scaled by 0.1 for all sites. The boundary layer height values for CMAQv5.0.2 come from WRFv3.4. DOI: https://doi.org/10.1525/elementa.2020.00158.f6

In Figure 6, we further explore the diurnal patterns of two important model processes (PBL height and mobile source emissions) and their temporal relationship to observed and modeled NO_*X*_ mixing ratios and the resulting modeled NO_*X*_ bias in four urban areas for summer and winter of 2011. This figure includes modeled values from CMAQ v5.0.2 (i.e., the model version used in the 2011 simulation shown in Figures 4 and 5) as well as CMAQ v5.1 (the model version used starting in 2013 in Figures 4 and 5). The four urban areas in Figure 6 represent different regions of the country impacted by different types of meteorology: a location in the Northeast impacted by strong seasonal changes in temperature (Bronx, NY), a location in the mid-Atlantic (Washington, DC) with more modest seasonal variations, a location on the gulf coast (Houston, TX), and an inland location in the Central United States (St. Louis, MO). The grid cells chosen also illustrate emissions variability between grid cells representing down-town urban areas with high traffic (e.g., DC, Bronx, Houston) and grid cells that include highways (St. Louis, MO).We focus on mobile NO_*X*_ emissions for these figures since mobile sources are generally the dominant NO_*X*_ source in urban areas. We note that vehicle running emissions make up the majority of mobile source emissions for each of the four urban locations included in Figure 6.

Similar to comparisons across the contiguous United States shown in Figure 4, modeled NO_*X*_ mixing ratios in these areas are highest in the early morning and late evening hours when mobile activity is high and the surface mixing layer is relatively shallow. It is during these same times of day when summertime NO_*X*_ overpredictions are largest. Additionally, in the Bronx and St. Louis locations, the MB remains elevated overnight in the CMAQv5.0.2 simulation compared to daytime bias. When comparing summer and winter NO_*X*_ mixing ratios, we see higher observed and modeled mixing ratios in winter due to a combination of shallower boundary layer heights (Seidel et al., 2010) and a longer atmospheric NO_*X*_ lifetime (Beirle et al., 2011) in winter. We note that the modeled local nonroad and running NO_*X*_ emissions shown in Figure 6 do not change substantially from summer to winter, with summer emissions being 6%–18% higher than winter emissions depending on the city. Nationally, the seasonal behavior of emissions also does not change significantly (with exception of the EGU anomaly captured in 2014 and explained earlier), thus we use Figure 1 to show that the nonpoint sector is the only sector for which NO_*X*_ emissions increase in winter versus summer. Therefore, emissions are unlikely to explain higher modeled wintertime NO_*X*_ mixing ratios. The diurnal pattern of NO_*X*_ bias changes during winter, where the morning period shows a smaller peak overprediction than in summertime (and an underprediction at some hours in DC) while the peak evening bias is more pronounced than was seen in the summertime for three of the four cities (Bronx, Houston, and DC). These seasonal changes in the NO_*X*_ bias suggest that the modeling system is not capturing the real-world seasonality in one or more of the following processes: emissions magnitudes, atmospheric mixing, or processes that affect NO_*X*_ lifetime such as chemistry and deposition.

The right-hand panels in Figure 6 show a change in the 2011 model estimated NO_*X*_ with the shift from the CMAQ5.0.2 to CMAQv5.1 simulation, with emissions inputs held constant. The version CMAQv5.1 sensitivity also included updated WRF meteorology inputs (from version 3.4 to 3.7). Similar to the results in Figures 4 and 5 when comparing 2012–2013 results, Figure 6 shows this system update had a substantial impact on the 2011 NO_*X*_ bias at these sites. In all four cities, NO_*X*_ mixing ratios predicted by CMAQv5.1 are lower than those predicted by CMAQv5.0.2 in both summer and winter. In the summer, peak morning NO_*X*_ overpredictions are reduced by a factor of 2–4 and peak evening NO_*X*_ overpredictions are reduced by a factor of 3–6 when updating to the more recent model version. The wintertime peak morning overprediction is reduced by a factor of 6–10 in the Bronx, Houston, and St. Louis, with morning bias becoming an underprediction in DC using the newer modeling system. Similarly, the peak evening overprediction is reduced by a factor of approximately 2–5 in each of the four cities with the newer v5.1 modeling system compared to v5.0.2.

**Figure 7.**
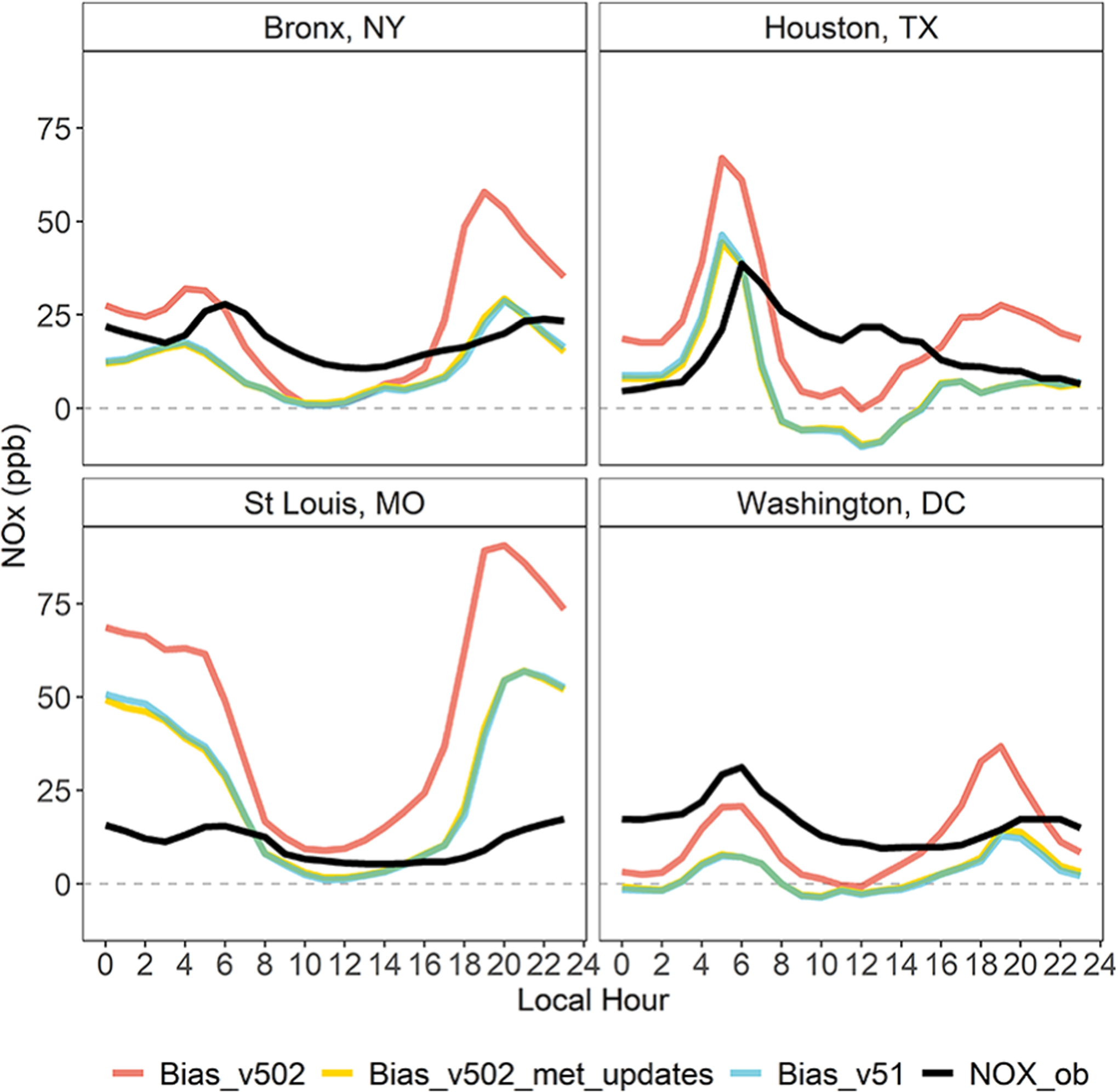
Sensitivity results showing diurnal profiles of summer mean NO_*X*_ observations and model mean bias for sites shown in Figure 6. The Community Multiscale Air Quality model version for both the base case and the sensitivity case is v5.0.2. DOI: https://doi.org/10.1525/elementa.2020.00158.f7

To better explain the change in NO_*X*_ bias seen at these sites, we leverage the evaluation of CMAQv5.1 presented in Appel et al. (2017). The incremental testing in that study included a CMAQ sensitivity to updates in WRF and CMAQ related to transport and vertical mixing (U.S. EPA, 2015b). The evaluation of this sensitivity found the net effect of these model changes in summer was an increase in vertical mixing, particularly in the late afternoon and early evening. This resulted in a decrease in PM_2.5_ at many urban locations which was attributed to a decrease in primary emitted elemental carbon and organic carbon and an increase in maximum daily 8-h average ozone attributed to a decrease in NO_*X*_ titration. Changes in PM_2.5_ and ozone in winter were found to be generally small with the exception of a relatively large increase in PM_2.5_ (>2.5 μg m^−3^) in the San Joaquin Valley of California. Using this sensitivity simulation, Figure S20 maps the change in 4–9 AM average NO_*X*_ between CMAQv5.0.2 and CMAQv5.1 and shows how much of this change is from the meteorology updates (S20e and f) and how much is from chemistry and other updates in CMAQv5.1 (S20g and h). In both summer and winter, the impacts from the more aggressive vertical mixing are most pronounced in urban areas while the impacts from chemistry and other model updates are spread out more regionally. While the winter decrease in NO_*X*_ is driven both by the mixing updates and by chemistry and other model updates, the largest decreases in summer NO_*X*_, particularly in urban areas, are almost exclusively from the more aggressive mixing within the boundary layer. This result is reflected in Figure 7 which shows the diurnal profile of the summer NO_*X*_ bias from Figure 6 but with the addition of the NO_*X*_ bias from the sensitivity (Bias_v502_met_updates).

The evaluation in Figures 4–7 focused on model estimated NO_*X*_ at surface monitoring locations. Another of the major updates in CMAQv5.1 was the change in chemical mechanism from CB05 to Carbon Bond 05 EPAv5.1 (CB05ev1). While this update did not impact the NO_*X*_ at the sites in Figures 6 and 7, evaluation against aloft measurements shows the importance of the chemistry changes on the NO_*Y*_ budget. Here, we present additional results from several July 2011 simulations testing the impacts of chemistry changes as described in Luecken et al. (2019), including a comparison against the chemistry in Carbon Bond 6 (CB6) which was first implemented in CMAQv5.2 (the model version used starting in the 2014 simulation). For this evaluation, modeled NO_*Y*_ aloft is compared to data from aircraft measurements taken during the DISCOVER-AQ field study to complement the evaluations using ground-level measurements from routine NO_*X*_ monitoring network sites. Measured NO_*Y*_ is shown in two ways: (1) as NO_*Y*_ measured using a chemiluminescence instrument (Ridley and Grahek, 1990), referred to in this article simply as “NO_*Y*_”, and (2) as the sum of NO_*Y*_ component species measured using a thermal dissociation laser-induced fluorescence instrument (Thornton et al., 1999), referred to as ∑NO_*Yi*_ in this article as was done in Simon et al. (2018). High temporal resolution time series of these paired modeled and observed NO_*Y*_ mixing ratios are available in the Supplement Figures S21–S34. These plots show large variability in measured NO_*Y*_ mixing ratios as the aircraft traveled between the free troposphere with relatively lower mixing ratios and into the planetary boundary layer with higher mixing ratios. Smaller variability in measurements made within the boundary layer was mostly driven by the aircraft’s location within or outside of the Baltimore and Washington, DC, urban plumes. Table 2 provides statistics (correlation, MB, and NMB) for model performance across all measured data pairs and also for pairs only within the measured boundary layer. Correlation across all data points ranges between 0.65 and 0.78 depending on which measurements and model simulations are used, with ∑NO_*Yi*_ measurements having higher correlation with modeled NO_*Y*_ than the chemiluminescence NO_*Y*_. Correlation drops somewhat (0.5–0.66) when using the subset of measurements taken within the boundary layer demonstrating that the model is doing a good job of simulating the boundary layer height and the resulting change in NO_*Y*_ mixing ratio between the boundary and the free troposphere. Luecken et al. (2019) also compared these model simulations to vertical profiles from the DISCOVER-AQ campaign and showed that the models capture the boundary layer height reasonably well and that the CB05e51 and CB6 chemical mechanisms generally simulate the vertical profile of several NO_*Y*_ components (ANs and peroxy nitrates [PNs]) better than the CB05 chemical mechanism. Correlations above 0.5 within the boundary layer suggest that that model is doing a reasonable job of capturing the location of the urban plume. While MB numbers differ above and within the boundary layer due to higher NO_*Y*_mixing ratioswithinthe boundary layer, the NMB is almost identical within the boundary layer and above the boundary layer (Table 2).This further suggests that any NO_*Y*_ biases are driven by systematic processes within the modeling system and are not isolated to localized emissions or meteorological processes in the Baltimore/DC area.

**Figure 8.**
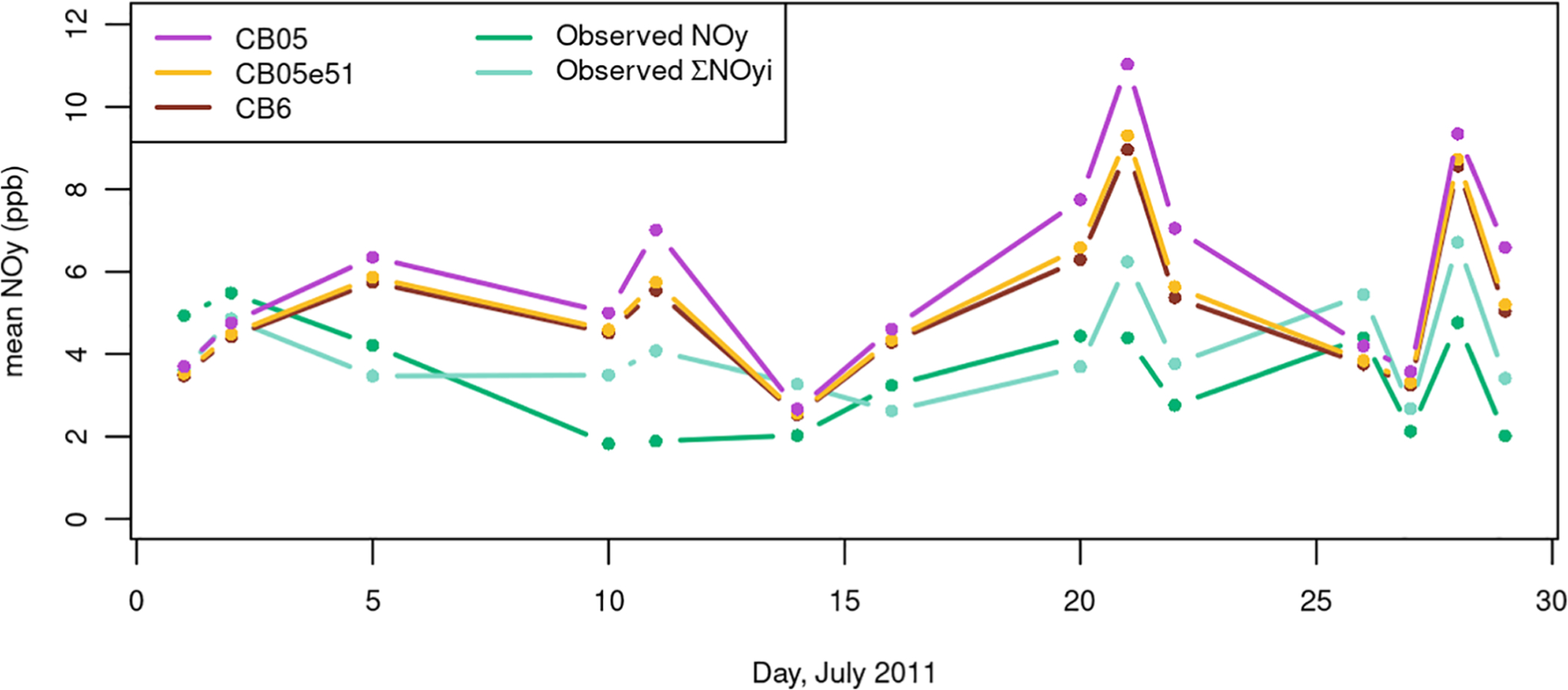
Daily averages of model NO_*Y*_ predictions compared to daily averages of aircraft measurements made during the July 2011 DISCOVER-AQ field study. Plotted data represent the subset of measurements taken within the observed boundary layer near Baltimore, MD. Model predictions are provided from simulations using three different chemical mechanisms: CB05, CB05e51, and CB6. Measurements are presented both as observed NO_*Y*_ from the chemiluminescence instrument and as the sum of measured NO_*Y*_ species from the thermal dissociation laser-induced fluorescence instrument. DISCOVER-AQ = Deriving Information on Surface Conditions from Column and Vertically Resolved Observations Relevant to Air Quality; CB = carbon bond. DOI: https://doi.org/10.1525/elementa.2020.00158.f8

It is informative to look more closely at the measurements made within the boundary layer to understand model performance and impacts of chemistry on locations that are most representative of NO_*Y*_ mixing ratios near the surface. Figure 8 shows daily averages of NO_*Y*_ from all model and measurement pairs that occurred within the measured PBL. The modeling system tends to overpredict the average aircraft measurements of NO_*Y*_ for most days of the field study. Predictions using CB6 and CB05e51 chemical mechanisms are very similar to each other. In addition, NO_*Y*_ predictions are closer to measurements using CB6/CB05e51 gas-phase chemistry compared to CB05, which is generally consistent with other studies (Qin et al., 2019). In addition, the model predictions match ∑NO_*Yi*_ more closely than NO_*Y*_. Together, the chosen measurement method and the chemical mechanism implemented in the modeling can substantially impact the magnitude of the estimated model NO_*Y*_ overpredictions: NMB = 75% when comparing CB05 modeling to measured NO_*Y*_ (the worst-performing combination); NMB = 27% when comparing CB6 modeling to measured ∑NO_*Yi*_ (the best performing combination).

**Figure 9.**
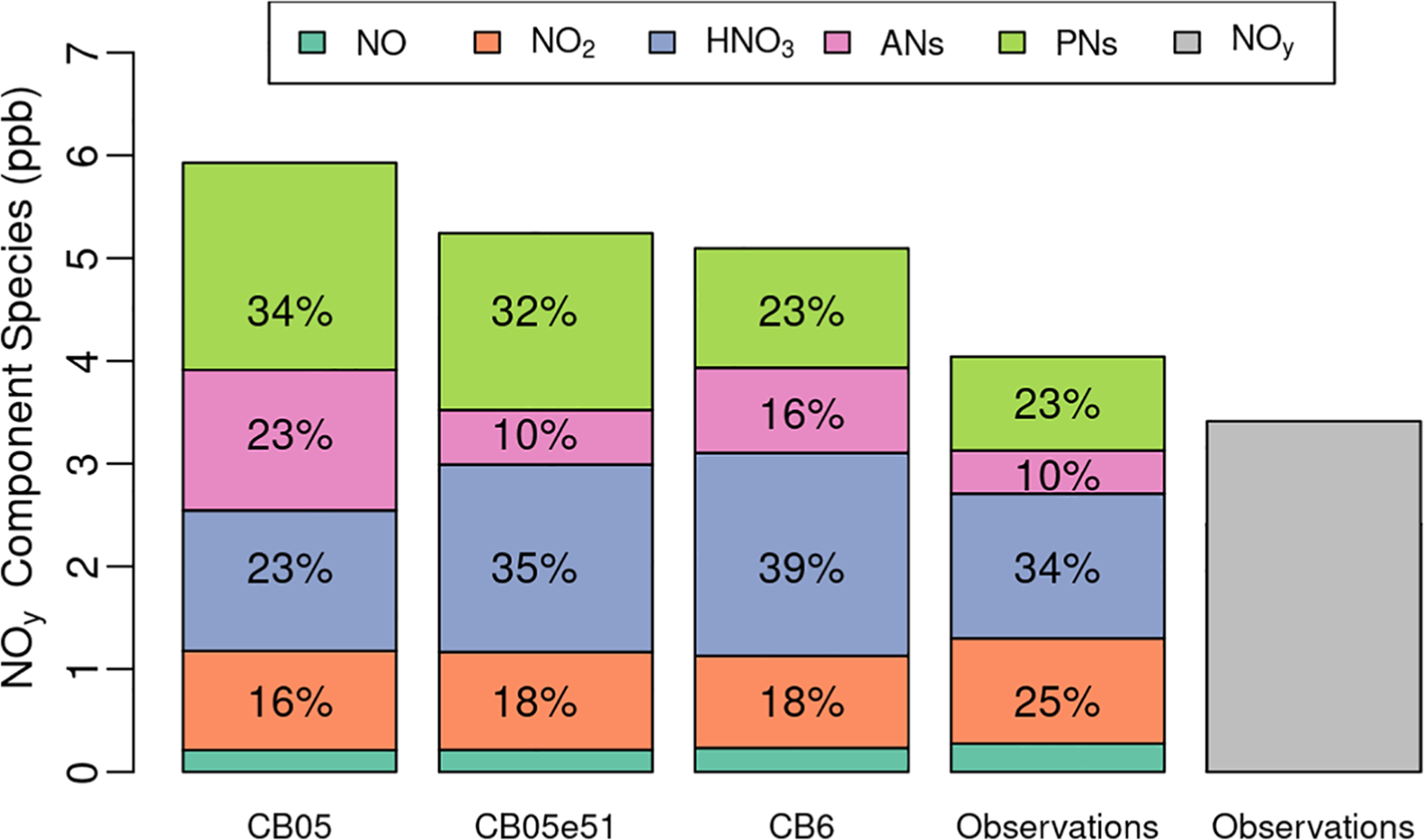
Aggregated model predictions and measurements of NO_*Y*_ species. All measurements considered are within the boundary layer over all flight days part of the July DISCOVER-AQ 2011 field study near Baltimore, MD. Bars representing observations are derived from both LIF (NO_2_, HNO_3_, alkyl nitrates, peroxy nitrates) and chemiluminescence (NO and NO_*Y*_) instruments. NO_Y_ is shown as a gray bar. DISCOVER-AQ = Deriving Information on Surface Conditions from Column and Vertically Resolved Observations Relevant to Air Quality. DOI: https://doi.org/10.1525/elementa.2020.00158.f9

Figure 9 shows the comparison of modeled NO_*Y*_ species against average DISCOVER-AQ aircraft measurements of NO_*Y*_ components averaged across all boundary layer measurements on all July 2011 flight days for model sensitivity simulations with the three chemical mechanisms tests. The mechanisms generally show relatively good agreement between modeled and measured NO_*X*_ mixing ratios but overestimates ANs (∑RNO_2_), PNs (∑RO_2_NO_2_), and HNO_3_. Changes in total NO_*X*_ mixing ratios between mechanisms (also shown in the map on Figure S19d) are quite small. The slight decrease in NO_*X*_ is due to the fact that CB6 has fewer pathways for recycling NO_*X*_ from nitrate (Luecken et al., 2019). These impacts occur on long enough timescales to result in a regional signal that is not localized to areas with high emissions (Figure S19d). The more substantial changes in the mechanism involve the aged NO_*Y*_ species. The CB6 chemical mechanism adds new pathways to transform ANs to HNO_3_ which is lost quickly from the atmosphere and also includes updates to the volatile organic compound and peroxy radical chemistry which impact PN levels. Consequently, AN and PN overpredictions improved from the CB05 to CB6 mechanism. The conversion of more NO_*Y*_ to HNO_3_ results in a shorter NO_*Y*_ lifetime and decreases the total NO_*Y*_ overprediction when compared against the aircraft measurements. Luecken et al. (2019) report that CB6 chemistry leads to 30%–40% more NO_*Y*_ removal through HNO_3_ deposition than CB05 chemistry. However, Figure 9 shows that HNO_3_ in the CB6 simulation is slightly overpredicted.

## Conclusions

Air quality model evaluation of NO_*X*_ was presented for simulations spanning the years 2002–2016 to provide context for other model applications and to better understand how model performance has changed over time throughout the evolution of the modeling system (i.e., model version, configuration, and inputs). Annual simulations show consistent wintertime underpredictions and a complex pattern of model bias for the summertime. The summertime performance and performance improvements highlight the importance of improvements to many parts of the modeling system.

The modeling system representation of NO_*X*_ has shown dramatic improvements in the performance of summertime predictions for more recent annual simulations compared to earlier (2002–2011) annual simulations. NO_*X*_ overpredictions are most severe when emissions are high and near-surface mixing is lowest, which often coincides with early morning and evening rush hours. The summertime morning NO_*X*_ bias dropped from between 28% and 57% for earlier years (2002–2012) to between −2% and 7% for later years (2013–2016). While multiple aspects of the modeling system and inputs were updated throughout the 15-year time series, sensitivity simulations for 2011 suggest that the update between the CMAQv5.0.2 version and the CMAQv5.1 version was a driving factor in the improved summertime model performance and increases in wintertime underpredictions. Major updates in CMAQv5.1 included improved treatment of vertical mixing, the treatment of alky nitrate chemistry, more sophisticated treatment of photolysis rates, and updates to the treatment of deposition. Sensitivity exercises to explore the impact of emissions timing of important sectors showed minimal impact on model performance. Investigation into the impacts of the chemistry changes showed that these were important for improving predictions of NO_*Y*_ (i.e., chemistry updates alone could reduce NO_*Y*_ MB by 35%–45%). However, the chemistry updates did not substantially impact summertime NO_*X*_ estimates in the Baltimore/DC urban area. Finally, sensitivity performed on locations used as case study showed that the model performance improvement observed for years 2013 and after can be attributed to updates in transport processes, particularly vertical mixing.

Different aspects of the modeling system can impact overall NO_*X*_ and NO_*Y*_ mixing ratios including the magnitude of annual emissions from multiple source types and the timing and location of those emissions. The methodology and underlying input data for estimating emissions have improved throughout the time series with major updates associated with the 2011 NEI (used as a basis for the 2011–2013 model simulations) and the 2014 NEI (used as a basis for the 2014–2016 model simulations). Different regulations have facilitated reductions in many of the important contributors to total NO_*X*_. In particular, the steep decreasing trend of mobile emissions over time and the differential nature of these decreases depend on the fleet composition (e.g., age of vehicles and their emission controls) and highlight the importance of using emissions that match the year of the model simulation. We recommend that studies evaluating air quality models recognize the limitations of using NO_*X*_ emissions that do not match the year of the model simulation to reduce the uncertainty on their diagnosis of model-observation comparisons.

Furthermore, future studies focused on NO_*X*_ model performance should include satellite total column measurements of NO_2_ in addition to in situ measurements to minimize the confounding impact of vertical mixing processes. Total column measurements can provide additional spatial and temporal coverage not available from surface monitoring networks; however, they also have their own uncertainties and use assumptions about vertical profiles of NO_2_ through the boundary layer, the free troposphere, and the stratosphere that are generally based on a priori model predictions. Additionally, evaluation of PBL height and mixing within the PBL against ceilometer or lidar measurements would provide more information on the model’s ability to capture these features of the atmosphere. Further analysis could also focus on whether finer resolution modeling could better capture spatial NO_*X*_ gradients in urban areas and improve model correlation statistics. Finally, research studies focused on isolating NO_*X*_ emissions from specific sources and source categories would be valuable toward evaluating specific components of the inventory. This includes fieldwork characterizing natural emissions sources (e.g., lightning and biogenic) and the anthropogenic categories discussed in this analysis.

## Supplementary Material

Supplement1 **Figure S1.** NO_*X*_ monitoring sites by National Oceanic and Atmospheric Administration (NOAA) Climate Regions (top) and data availability by site (bottom). Bottom figure shows the number of years with data available by site for the period 2002–2016. Note that the NOAA Climate Region Northern Rockies and Plains (MT, WY, ND, SD, and NE) has been combined with the Northwest Region (WA, OR, and ID) due to the limited number of available monitors in many of these states.
**Figure S2.** Modeled annual average of oxidized nitrogen species for winter (top four-plot panel, labeled as Q1) and summer (bottom 4-plot panel, labeled as Q3) of 2015. For each four-plot panel, top left represents NO_*X*_ (NO + NO_2_), top right represents NO_*Z*_ (the difference between NO_*Y*_ and *NO*_*X*_), bottom left represents NO_*Y*_ (all oxidized species), and bottom right the ratio of NO_*X*_ to NO_*Y*_.
**Figure S3.** Number of sites used to calculate the normalized mean bias of morning NO_*X*_ in Figures S4–S6. Morning hours considered are 4–9 AM LST. Number of sites is shown by year and season.
**Figure S4.** Normalized mean bias of morning modeled NO_*X*_ and observed NO_*X*._ Morning hours are 4–9 AM LST. Data have been aggregated by season for each annual simulation across all monitors in U.S. domain (Figure S1).
**Figure S5.** Normalized mean bias of midday modeled NO_*X*_ and observed NO_*X*._ Midday hours are 11 AM–3 PM LST. Data have been aggregated by season for each annual simulation across all monitors in U.S. domain (Figure S1).
**Figure S6.** Normalized mean bias of evening modeled NO_*X*_ and observed NO_*X*._ Evening hours are 4–9 PM LST. Data have been aggregated by season for each annual simulation across all monitors in U.S. domain (Figure S1).
**Figure S7.** Number of sites used to calculate normalized mean bias of morning NO_*X*_ in Figure 4. Morning hours considered are 4–9 AM LST. Number of sites is shown by region and year/season. West = CA and NV; Northwest = OR, WA, ID, MT, NE, ND, SD, and WY; Upper Midwest = IA, MI, MN, and WI; Ohio Valley = IL, IN, KY, MO, OH, TN, and WV; Northeast = CT, DE, ME, MD, MA, NH, MJ, NY, PA, RI, and VT; Southwest = AZ, CO, NM, and UT; South = AR, KS, LA, MS, OK, and TX; Southeast = AL, FL, GA, NC, SC, and VA
**Figure S8.** METAR stations. Weather stations used to evaluate the Weather Research and Forecasting meteorological model in Figures S9–S11.
**Figure S9.** Mean bias of 2 m temperature. Data have been aggregated by season for each annual simulation across all METAR stations in U.S. domain (Figure S8).
**Figure S10.** Mean bias of 10-m windspeed. Data have been aggregated by season for each annual simulation across all METAR stations in U.S. domain (Figure S8).
**Figure S11.** Mean bias of 2 m water vapor mixing ratio. Water vapor mixing ratio is a measure of the moisture in the air and is approximately equal to specific humidity. Data have been aggregated by season for each annual simulation across all METAR stations in U.S. domain (Figure S8).
**Figure S12.** Normalized mean error of morning modeled NO_*X*_ and observed NO_*X*_. Morning hours are 4–9 AM LST. Data have been aggregated by season for each annual simulation across monitors in multiple regions defined by National Oceanic and Atmospheric Administration climate region (Figure S1). West = CA and NV; Northwest = OR, WA, ID, MT, NE, ND, SD, and WY; Upper Midwest = IA, MI, MN, and WI; Ohio Valley = IL, IN, KY, MO, OH, TN, and WV; Northeast = CT, DE, ME, MD, MA, NH, MJ, NY, PA, RI, and VT; Southwest = AZ, CO, NM, and UT; South = AR, KS, LA, MS, OK, and TX; Southeast = AL, FL, GA, NC, SC, and VA.
**Figure S13.** Correlation (*R*^2^) of morning modeled NO_*X*_ and observed NO*X*. Morning hours are 4–9 AM LST. Data have been aggregated by season for each annual simulation across monitors in multiple regions defined by National Oceanic and Atmospheric Administration climate region (Figure S1). West = CA and NV; Northwest = OR,WA, ID, MT, NE, ND, SD, and WY; Upper Midwest = IA, MI, MN, and WI; Ohio Valley = IL, IN, KY, MO, OH, TN, and WV; Northeast = CT, DE, ME, MD, MA, NH, MJ, NY, PA, RI, and VT; Southwest = AZ, CO, NM, and UT; South = AR, KS, LA, MS, OK, and TX; Southeast = AL, FL, GA, NC, SC, and VA.
**Figure S14.** As in Figure 5 of the main paper, but for modeled NO_*Y*_ – observed NO_*X.*_
**Figure S15.** Mean bias of morning modeled NO_*X*_ and observed NO_*X*_ at surface monitors for 2002–2016. Morning hours are 4–9 AM LST. Data have been aggregated for winter (left column) and summer (right column) months. Warm colors indicate model overprediction and cool colors underprediction.
**Figure S16.** Nonroad diurnal emissions profiles used in the sensitivity test. The 2011 base simulation (“old”) and sensitivity simulation are shown for the following sectors: construction equipment, residential lawn and garden equipment, commercial lawn and garden equipment, and agricultural equipment.
**Figure S17.** Map of counties and parishes depicting the source of temporal data. Locations shown in gray indicate that Environmental Protection Agency (EPA) default data (derived from vehiclen travel information system [VTRIS]) were used versus state submitted data (green and yellow). Sensitivity run #2 replaced all state-submitted temporal profile data with EPA VTRIS derived profiles (except California).
**Figure S18.** Example day-of-year temporal profile for Electrical Generating Unit sources (fuel = “other”) in Eastern Virginia. Up to 7% of the annual emissions = are emitted on a single day.
**Figure S19.** Change in the July 2011 average of modeled NO_*X*_ mixing ratios (ppb) resulting from sensitivity tests. (a) Nonroad emissions adjustments, (b) alternative heavy-duty onroad temporal profiles, (c) alternative temporal allocation of Continuous Emissions Monitors for year 2011 (12-km horizontal grid resolution, all hours averaged), and (d) CB6 chemical mechanism versus CB05 chemical mechanism (12-km horizontal grid resolution, all hours averaged). CB = carbon bond.
**Figure S20.** Change in modeled NO_*X*_ mixing ratio (ppb) resulting from updating from CMAQv5.0.2 to CMAQv5.1 Maps show the spatial distribution of this change for (a) January 2011 average 4–9 AM LST NO_*X*_ from CMAQv5.0.2 simulation, (b) same as (a) but for July 2011, (c and d) difference between CMAQv5.1 and CMAQv5.0.2, (e and f) difference between meteorology sensitivity and CMAQv5.0.2, and (g and h) difference between CMAQv5.1 and meteorology sensitivity. CMAQ = Community Multiscale Air Quality.
**Figure S21.** Paired model and observed NO_*Y*_ from Deriving Information on Surface Conditions from Column and Vertically Resolved Observations Relevant to Air Quality Baltimore flight on July 1, 2011.
**Figure S22.** Paired model and observed NO_*Y*_ from Deriving Information on Surface Conditions from Column and Vertically Resolved Observations Relevant to Air Quality Baltimore flight on July 2, 2011.
**Figure S23.** Paired model and observed NO_*Y*_ from Deriving Information on Surface Conditions from Column and Vertically Resolved Observations Relevant to Air Quality Baltimore flight on July 5, 2011.
**Figure S24.** Paired model and observed NO_*Y*_ from Deriving Information on Surface Conditions from Column and Vertically Resolved Observations Relevant to Air Quality Baltimore flight on July 10, 2011.
**Figure S25.** Paired model and observed NO_*Y*_ from Deriving Information on Surface Conditions from Column and Vertically Resolved Observations Relevant to Air Quality Baltimore flight on July 11, 2011.
**Figure S26.** Paired model and observed NO_*Y*_ from Deriving Information on Surface Conditions from Column and Vertically Resolved Observations Relevant to Air Quality Baltimore flight on July 14, 2011.
**Figure S27.** Paired model and observed NO_*Y*_ from Deriving Information on Surface Conditions from Column and Vertically Resolved Observations Relevant to Air Quality Baltimore flight on July 16, 2011.
**Figure S28.** Paired model and observed NO_*Y*_ from Deriving Information on Surface Conditions from Column and Vertically Resolved Observations Relevant to Air Quality Baltimore flight on July 20, 2011.
**Figure S29.** Paired model and observed NO_*Y*_ from Deriving Information on Surface Conditions from Column and Vertically Resolved Observations Relevant to Air Quality Baltimore flight on July 21, 2011.
**Figure S30.** Paired model and observed NO_*Y*_ from Deriving Information on Surface Conditions from Column and Vertically Resolved Observations Relevant to Air Quality Baltimore flight on July 22, 2011.
**Figure S31.** Paired model and observed NO_*Y*_ from Deriving Information on Surface Conditions from Column and Vertically Resolved Observations Relevant to Air Quality Baltimore flight on July 26, 2011.
**Figure S32.** Paired model and observed NO_*Y*_ from Deriving Information on Surface Conditions from Column and Vertically Resolved Observations Relevant to Air Quality Baltimore flight on July 27, 2011.
**Figure S33.** Paired model and observed NO_*Y*_ from Deriving Information on Surface Conditions from Column and Vertically Resolved Observations Relevant to Air Quality Baltimore flight on July 28, 2011.
**Figure S34.** Paired model and observed NO_*Y*_ from Deriving Information on Surface Conditions from Column and Vertically Resolved Observations Relevant to Air Quality Baltimore flight on July 29, 2011.
**Table S1.** List of model chemical mechanism species that were assigned to each measured NO_*Y*_ species.

## Figures and Tables

**Table 1.** Details of model simulations used in this study. DOI: https://doi.org/10.1525/elementa.2020.00158.t1

Year	Air Quality Model	Chemical Mechanism Aerosol Module	Meteorological Model	NEI Version (Emissions Platform)	Onroad Model	Reference/Metadata
2002	CMAQV5.0.2	CB05TUCL AERO6	WRFV3.4	2002 NEI v3 (2002af)	MOVES2010b	https://doi.org/10.15139/S3/IF4U6D
2003	CMAQV5.0.2	CB05TUCL AERO6	WRFV3.4	2002 NEI v3 (2003af)	Interpolated between 2002 and 2005	https://doi.org/10.15139/S3/E08VGK
2004	CMAQV5.0.2	CB05TUCL AERO6	WRFV3.4	2005 NEI v3 (2005ct_04)	Interpolated between 2002 and 2005	https://doi.org/10.15139/S3/Z0XTML
2005	CMAQV5.0.2	CB05TUCL AERO6	WRFV3.4	2005 NEI v3 (2005ct_04)	MOVES2010b	https://doi.org/10.15139/S3/GLQYWT
2006	CMAQV5.0.2	CB05TUCL AERO6	WRFV3.4	2008 NEI v3 (2007ed_06)	MOVES2010b	https://doi.org/10.15139/S3/56JKOO
2007	CMAQV5.0.2	CB05TUCL AERO6	WRFV3.4	2008 NEI v3 (2007rh)	MOVES2010b	https://doi.org/10.15139/S3/8LVCJM
2008	CMAQV5.0.2	CB05TUCL AERO6	WRFV3.4	2008 NEI v3 (2008ab)	MOVES2010b	https://doi.org/10.15139/S3/JPHKEV
2009	CMAQv5.0.2	CB05TUCL AERO6	WRFV3.4	2008 NEI v3 (2009ef)	MOVES2010b	https://doi.org/10.15139/S3/MEDEWM
2010	CMAQV5.0.2	CB05TUCL AERO6	WRFV3.4	2008 NEI v3 (2007ed_10)	MOVES2010b	https://doi.org/10.15139/S3/YIPTDY
2011	CMAQv5.0.2	CB05TUCL AERO6	WRFV3.4	2011 NEI v1 (2011ed)	MOVES2010b	https://doi.org/10.15139/S3/NS8Y6C
2011	CMAQv5.0.2_met_updates	CB05TUCL AERO6	WRFV3.7	2011 NEI v1 (2011ed)	MOVES2010b	Appel et al. (2017)
2011	CMAQv5.1	CB05e51 AERO6	WRFV3.7	2011 NEI v1 (2011ed)	MOVES2010b	Appel et al. (2017)
2012	CMAQv5.0.2	CB05TUCL AERO6	WRFV3.4	2011 NEI v2 (2011ed_12)	MOVES2014a	https://doi.org/10.15139/S3/YXQNRW
2013	CMAQv5.1	CB05e51 AERO6	WRFV3.7.1	2011 NEI v2 (2013ej)	MOVES2014a	https://doi.org/10.15139/S3/FQO7IS
2014	CMAQv5.2	CB6r3 AERO6NVPOA	WRFV3.8.1	2014 NEI v1 (2014fb)	MOVES2014a	https://doi.org/10.15139/S3/XYW3HL
2015	CMAQv5.2.1	CB6r3 AERO6NVPOA	WRFV3.8	2015 NEI v1 (2015fd)	MOVES2014a	Kelly et al. (2019)
2016	CMAQv5.2.1	CB6r3 AERO6	WRFV3.8	2016 NEI alpha (2016fe)	MOVES2014b	Appel et al. (2021)
2016	CMAQv5.3	CB6r3 AERO7	WRFV3.8	2016 NEI beta (2016ff)	MOVES2014b	Appel et al. (2021)

WRF = Weather Research and Forecasting; CMAQ = Community Multiscale Air Quality; NEI = National Emission Inventory; MOVES = Motor Vehicle Emission Simulator.

**Table 2.** Model NO_*Y*_ performance statistics compared to aircraft measurements during 2011 DISCOVER-AQ Baltimore. DOI: https://doi.org/10.1525/elementa.2020.00158.t2

Model Chemical Mechanism Versus Measurement Method	Subset	Correlation	MB (ppb)	NMB (%)
CB05 versus chemiluminescence NO_*Y*_	Boundary layer only	.50	2.6	74.9
	All measurements	.65	1.8	76.9
CB05 versus ∑NO_*Yi*_	Boundary layer only	.63	1.9	47.9
	All measurements	.76	1.5	51.0
CB05e51 versus chemiluminescence NO_*Y*_	Boundary layer only	.55	1.8	54.0
	All measurements	.68	1.3	53.7
CB05e51 versus ∑NO_*Yi*_	Boundary layer only	.66	1.2	30.0
	All measurements	.78	0.9	30.8
CB6 versus chemiluminescence NO_*Y*_	Boundary layer only	.55	1.7	49.8
	All measurements	.68	1.2	49.0
CB6 versus ∑NO_*Yi*_	Boundary layer only	.66	1.1	26.5
	All measurements	.78	0.8	26.8

DISCOVER-AQ = Deriving Information on Surface Conditions from Column and Vertically Resolved Observations Relevant to Air Quality; MB = mean bias; NMB = normalized mean bias; CB = carbon bond.
