# Supplementary material for: Evaluation of 15 years of modeled atmospheric oxidized nitrogen compounds across the contiguous United States
Source: Elementa (Wash D C). Author manuscript; Available in PMC 2022 May 7. (PMC8128711; doi:10.1525/elementa.2020.00158)
Supplement: Supplement1 — Figure S1. NOX monitoring sites by National Oceanic and Atmospheric Administration (NOAA) Climate Regions (top) and data availability by site (bottom). Bottom figure shows the number of years with data available by site for the period 2002–2016. Note that the NOAA Climate Region Northern Rockies and Plains (MT, WY, ND, SD, and NE) has been combined with the Northwest Region (WA, OR, and ID) due to the limited number of available monitors in many of these states. Figure S2. Modeled annual average of oxidized nitrogen species for winter (top four-plot panel, labeled as Q1) and summer (bottom 4-plot panel, labeled as Q3) of 2015. For each four-plot panel, top left represents NOX (NO + NO2), top right represents NOZ (the difference between NOY and NOX), bottom left represents NOY (all oxidized species), and bottom right the ratio of NOX to NOY. Figure S3. Number of sites used to calculate the normalized mean bias of morning NOX in Figures S4–S6. Morning hours considered are 4–9 AM LST. Number of sites is shown by year and season. Figure S4. Normalized mean bias of morning modeled NOX and observed NOX. Morning hours are 4–9 AM LST. Data have been aggregated by season for each annual simulation across all monitors in U.S. domain (Figure S1). Figure S5. Normalized mean bias of midday modeled NOX and observed NOX. Midday hours are 11 AM–3 PM LST. Data have been aggregated by season for each annual simulation across all monitors in U.S. domain (Figure S1). Figure S6. Normalized mean bias of evening modeled NOX and observed NOX. Evening hours are 4–9 PM LST. Data have been aggregated by season for each annual simulation across all monitors in U.S. domain (Figure S1). Figure S7. Number of sites used to calculate normalized mean bias of morning NOX in Figure 4. Morning hours considered are 4–9 AM LST. Number of sites is shown by region and year/season. West = CA and NV; Northwest = OR, WA, ID, MT, NE, ND, SD, and WY; Upper Midwest = IA, MI, MN, and WI; Ohio Valley = IL, IN, KY, [file NIHMS1701777-supplement-Supplement1.docx]

**Supplemental Material**

**Evaluation of 15 years of modeled atmospheric oxidized nitrogen compounds across the contiguous United States**

Claudia Toro^1^, Kristen Foley*^2^, Heather Simon^2^, Barron Henderson^2^, Kirk R. Baker^2^, Alison Eyth^2^, Brian Timin^2^, Wyat Appel^2^, Deborah Luecken^2^, Megan Beardsley^1^, Darrell Sonntag^1^, Norm Possiel^2^, Sarah Roberts^1^

^1^U.S. Environmental Protection Agency, Ann Arbor, Michigan, United States

^2^U.S. Environmental Protection Agency, Research Triangle Park, North Carolina, United States

*Corresponding author: foley.kristen@epa.gov

**List of Contents:**

**Figure S1.** **NO_X_ monitoring sites by NOAA Climate Regions (top) and data availability by site (bottom).** Bottom figure shows number of years with data available by site for the period 2002-2016. Note that the NOAA Climate Region Northern Rockies and Plains (MT, WY, ND, SD, NE) has been combined with the Northwest Region (WA, OR, ID) due to limited number of available monitors in many of these states.

**Figure S2**. **Modeled annual average of oxidized nitrogen species for winter (top 4-plot panel, labeled as Q1) and summer (bottom 4-plot panel, labeled as Q3) of 2015**. For each 4-plot panel, top left represents NO_X_ (NO+ NO_2_), top right represents NOz (the difference between NO_Y_ and NO_X_), bottom left represents NOy (all oxidized species), and bottom right the ratio of NO_X_ to NO_Y_.

**Figure S3.** **Number of sites used to calculate Normalized Mean Bias of morning NO_X_ in Figure S4 – S6.**

Morning hours considered are 4-9 a.m. LST. Number of sites is shown by year and season.

**Figure S4.** **Normalized Mean Bias (NMB) of morning modeled NO_X_ and observed NO_X_**_._ Morning hours are 4 – 9 a.m. LST. Data have been aggregated by season for each annual simulation across all monitors in US domain (Fig. S1).

**Figure S5.** **Normalized Mean Bias (NMB) of midday modeled NO_X_ and observed NO_X_**_._ Midday hours are 11 a.m. – 3 p.m. LST. Data have been aggregated by season for each annual simulation across all monitors in US domain (Fig. S1).

**Figure S6.** **Normalized Mean Bias (NMB) of evening modeled NO_X_ and observed NO_X_**_._ Evening hours are 4 p.m. – 9 p.m. LST. Data have been aggregated by season for each annual simulation across all monitors in US domain (Fig. S1).

**Figure S7.** **Number of sites used to calculate Normalized Mean Bias of morning NO_X_ in Figure 4.** Morning hours considered are 4-9 a.m. LST. Number of sites is shown by region and year/season. West = CA, NV; Northwest = OR, WA, ID, MT, NE, ND, SD, WY; Upper Midwest = IA, MI, MN, WI; Ohio Valley = IL, IN, KY, MO, OH, TN, WV; Northeast = CT, DE, ME, MD, MA, NH, MJ, NY, PA, RI, VT; Southwest = AZ, CO, NM, UT; South = AR, KS, LA, MS, OK, TX; Southeast = AL, FL, GA, NC, SC, VA

**Figure S8. METAR stations.** Weather stations used to evaluate the WRF meteorological model in Figures S9 – S11.

**Figure S9. Mean Bias (MB) of 2 meters temperature.** Data have been aggregated by season for each annual simulation across all METAR stations in US domain (Fig. S8).

**Figure S10. Mean Bias (MB) of 10 meters windspeed.** Data have been aggregated by season for each annual simulation across all METAR stations in US domain (Fig. S8).

**Figure S11. Mean Bias (MB) of 2 meters water vapor mixing ratio.** Water vapor mixing ratio is a measure of the moisture in the air and is approximately equal to specific humidity. Data have been aggregated by season for each annual simulation across all METAR stations in US domain (Fig. S8).

**Figure S12.** **Normalized Mean Error (NME) of morning modeled NO_X_ and observed NO_X_**_._ Morning hours are 4-9 a.m. LST. Data has been aggregated by season for each annual simulation across monitors in multiple regions defined by NOAA climate region (Fig. S1). West = CA, NV; Northwest = OR, WA, ID, MT, NE, ND, SD, WY; Upper Midwest = IA, MI, MN, WI; Ohio Valley = IL, IN, KY, MO, OH, TN, WV; Northeast = CT, DE, ME, MD, MA, NH, MJ, NY, PA, RI, VT; Southwest = AZ, CO, NM, UT; South = AR, KS, LA, MS, OK, TX; Southeast = AL, FL, GA, NC, SC, VA

**Figure S13**. **Correlation (R^2^) of morning modeled NO_X_ and observed NO_X_**_._ Morning hours are 4-9 a.m. LST. Data has been aggregated by season for each annual simulation across monitors in multiple regions defined by NOAA climate region (Fig. S1). West = CA, NV; Northwest = OR, WA, ID, MT, NE, ND, SD, WY; Upper Midwest = IA, MI, MN, WI; Ohio Valley = IL, IN, KY, MO, OH, TN, WV; Northeast = CT, DE, ME, MD, MA, NH, MJ, NY, PA, RI, VT; Southwest = AZ, CO, NM, UT; South = AR, KS, LA, MS, OK, TX; Southeast = AL, FL, GA, NC, SC, VA

**Figure S14. As in Figure 5 of the main paper, but for modeled NO_Y_ – observed NO_X_**

**Figure S15. Mean bias of morning Modeled NO_X_ and Observed NO_X_ at surface monitors for 2002–2016.** Morning hours are 4-9 a.m. LST. Data has been aggregated for winter (left column) and summer (right column) months. Warm colors indicate model over-prediction and cool colors under-prediction.

**Figure S16.** **Nonroad diurnal emissions profiles used in sensitivity test**. The 2011 base simulation (“old”) and sensitivity simulation are shown for the following sectors: construction equipment, residential lawn and garden equipment, commercial lawn and garden equipment and agricultural equipment.

**Figure S17.** **Map of counties and parishes depicting source of temporal data.** Locations shown in grey indicate that EPA default data (derived from VTRIS) was used vs. state submitted data (green and yellow). Sensitivity run #2 replaced all state-submitted temporal profile data with EPA VTRIS derived profiles (except California).

**Figure S18.** **Example day-of-year temporal profile for EGU sources (fuel = “other”) in Eastern Virginia**. X-axis provides Julian day for 2011. Y-axis provides fractional attribution of annual emissions to each day of the year. Up to 7% of the annual emissions are emitted on a single day.

**Figure S19.** **Change in the July 2011 average of modeled NO_X_ mixing ratio (ppb) resulting from sensitivity tests**. (a) nonroad emissions adjustments, (b) alternative heavy-duty onroad temporal profiles, (c) alternative temporal allocation of CEMs for year 2011 (12-km horizontal grid resolution, all hours averaged) and d) CB6 chemical mechanism versus CB05 chemical mechanism (12-km horizontal grid resolution, all hours averaged) .

**Figure S20.** **Change in modeled NOX mixing ratio (ppb) resulting from updating from CMAQv5.0.2 to CMAQv5.1** Maps show the spatial distribution of this change for (a) January 2011 average 4am – 9am LST NO_X_ from CMAQv5.0.2 simulation, (b) same as (a) but for July 2011, (c-d) difference between CMAQv5.1 and CMAQv5.0.2, (e-f) difference between meteorology sensitivity and CMAQv5.0.2, (g-h) difference between CMAQv5.1 and meteorology sensitivity.

**Figure S21. Paired model and observed NOy from DISCOVER-AQ Baltimore flight on July 1, 2011.**

**Figure S22. Paired model and observed NOy from DISCOVER-AQ Baltimore flight on July 2, 2011.**

**Figure S23. Paired model and observed NOy from DISCOVER-AQ Baltimore flight on July 5, 2011.**

**Figure S24. Paired model and observed NOy from DISCOVER-AQ Baltimore flight on July 10, 2011.**

**Figure S25. Paired model and observed NOy from DISCOVER-AQ Baltimore flight on July 11, 2011.**

**Figure S26. Paired model and observed NOy from DISCOVER-AQ Baltimore flight on July 14, 2011.**

**Figure S27. Paired model and observed NOy from DISCOVER-AQ Baltimore flight on July 16, 2011.**

**Figure S28. Paired model and observed NOy from DISCOVER-AQ Baltimore flight on July 20, 2011.**

**Figure S29. Paired model and observed NOy from DISCOVER-AQ Baltimore flight on July 21, 2011.**

**Figure S30. Paired model and observed NOy from DISCOVER-AQ Baltimore flight on July 22, 2011.**

**Figure S31. Paired model and observed NOy from DISCOVER-AQ Baltimore flight on July 26, 2011.**

**Figure S32. Paired model and observed NOy from DISCOVER-AQ Baltimore flight on July 27, 2011.**

**Figure S33. Paired model and observed NOy from DISCOVER-AQ Baltimore flight on July 28, 2011.**

**Figure S34. Paired model and observed NOy from DISCOVER-AQ Baltimore flight on July 29, 2011.**

**Table S1. List of model chemical mechanism species that were assigned to each measured NOy species.**


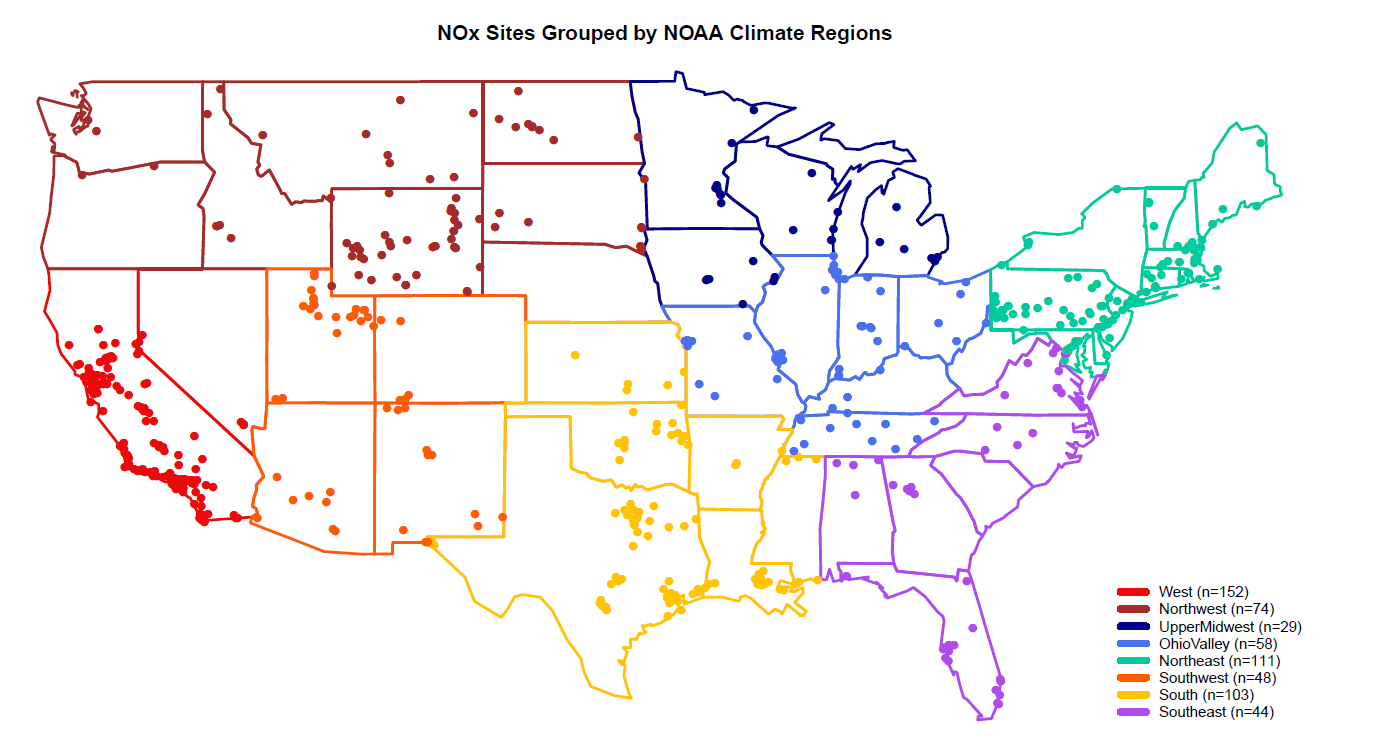


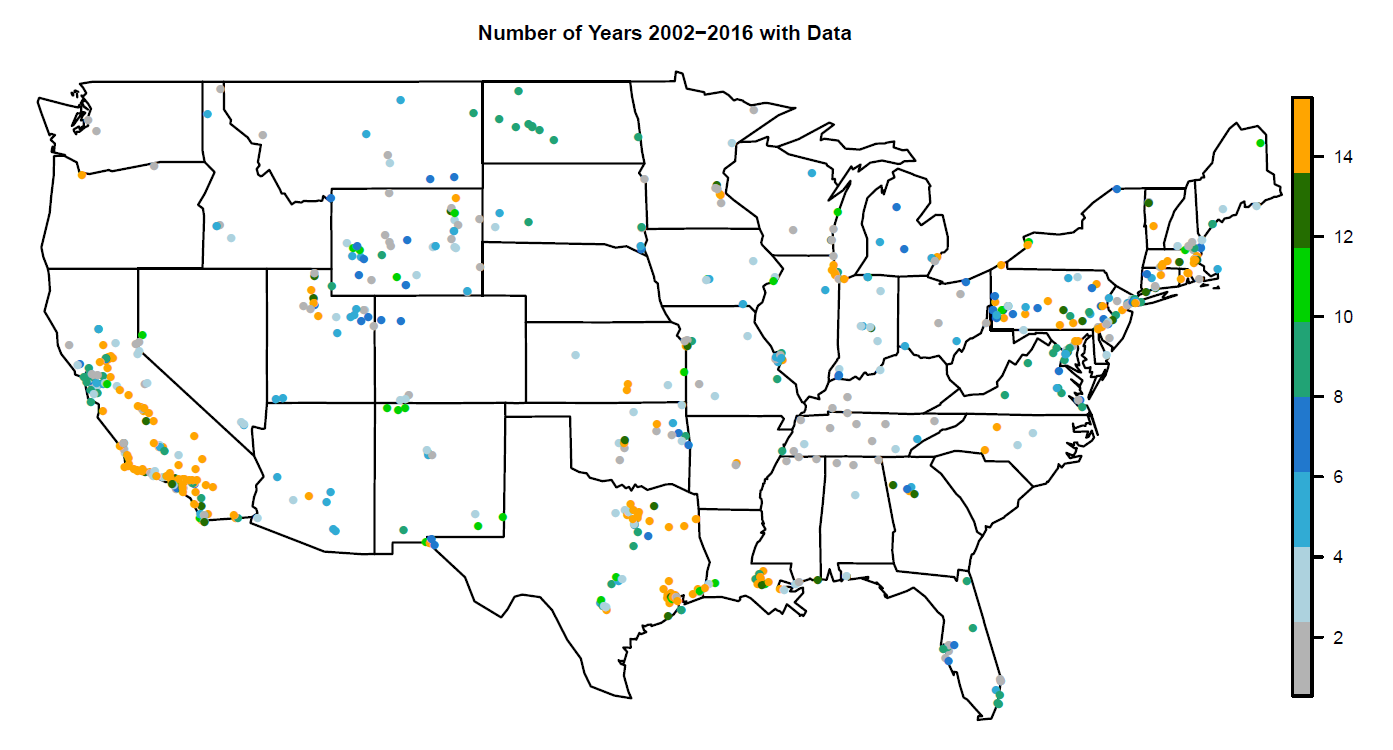


**Figure S1.** **NO_X_ monitoring sites by NOAA Climate Regions (top) and data availability by site (bottom).** Bottom figure shows number of years with data available by site for the period 2002-2016. Note that the NOAA Climate Region Northern Rockies and Plains (MT, WY, ND, SD, NE) has been combined with the Northwest Region (WA, OR, ID) due to limited number of available monitors in many of these states.


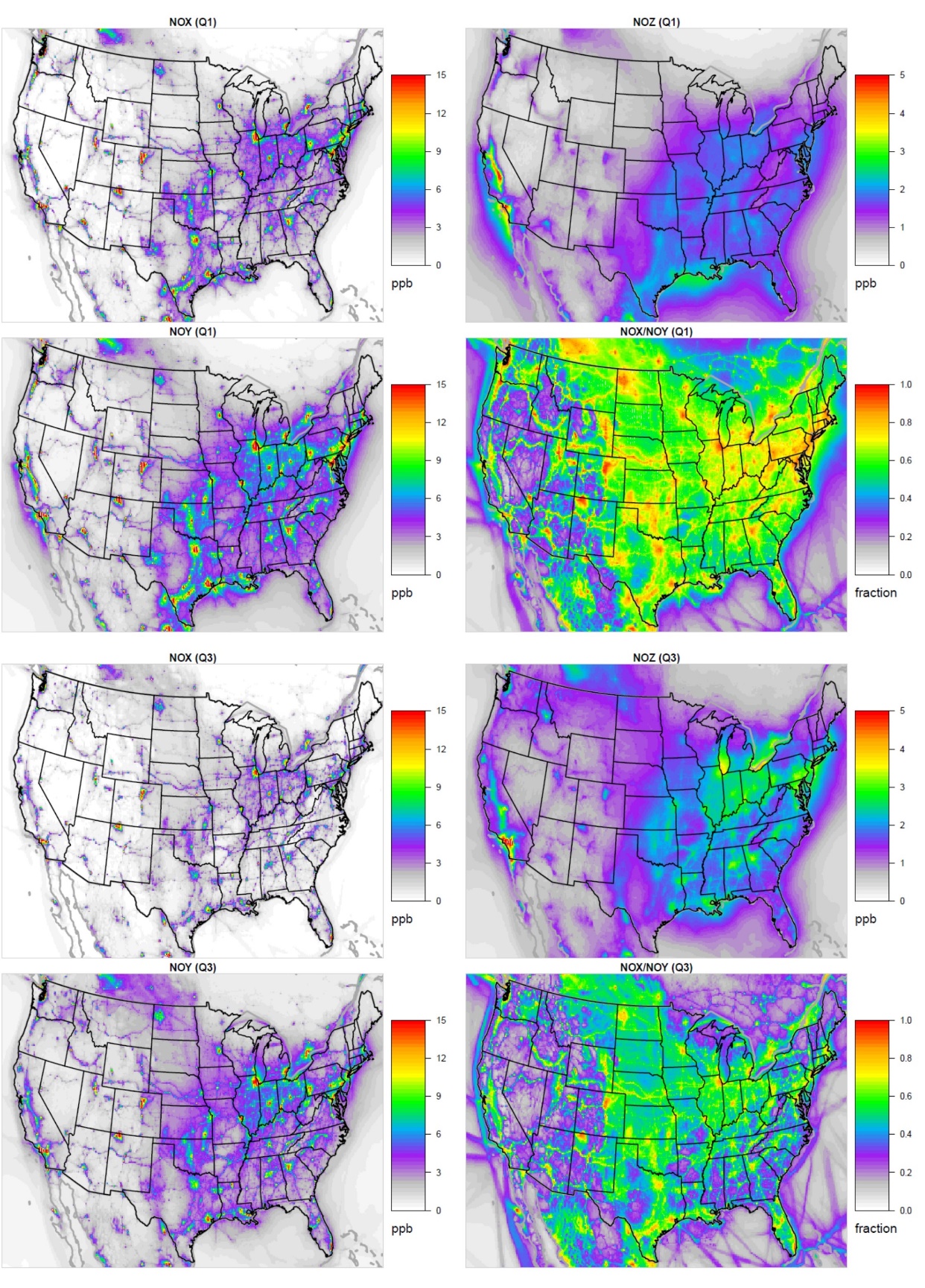


**Figure S2**. **Modeled annual average of oxidized nitrogen species for winter (top 4-plot panel, labeled as Q1) and summer (bottom 4-plot panel, labeled as Q3) of 2015**. For each 4-plot panel, top left represents NO_X_ (NO+ NO_2_), top right represents NOz (the difference between NO_Y_ and NO_X_), bottom left represents NOy (all oxidized species), and bottom right the ratio of NO_X_ to NO_Y_.
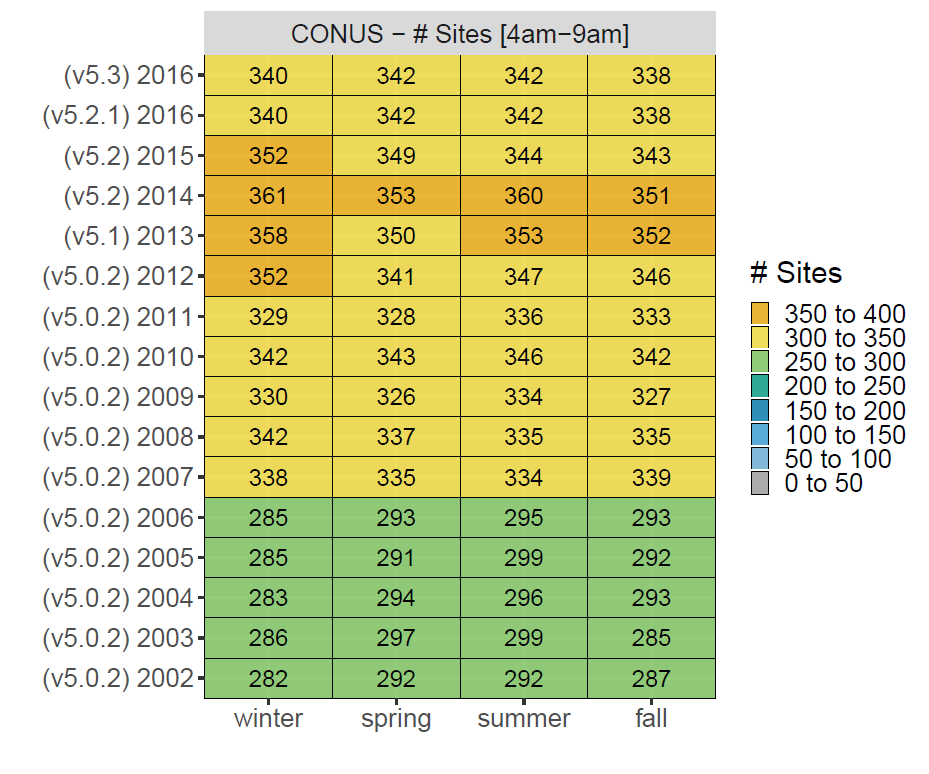


**Figure S3.** **Number of sites used to calculate Normalized Mean Bias of morning NO_X_ in Figures S4 – S6.**


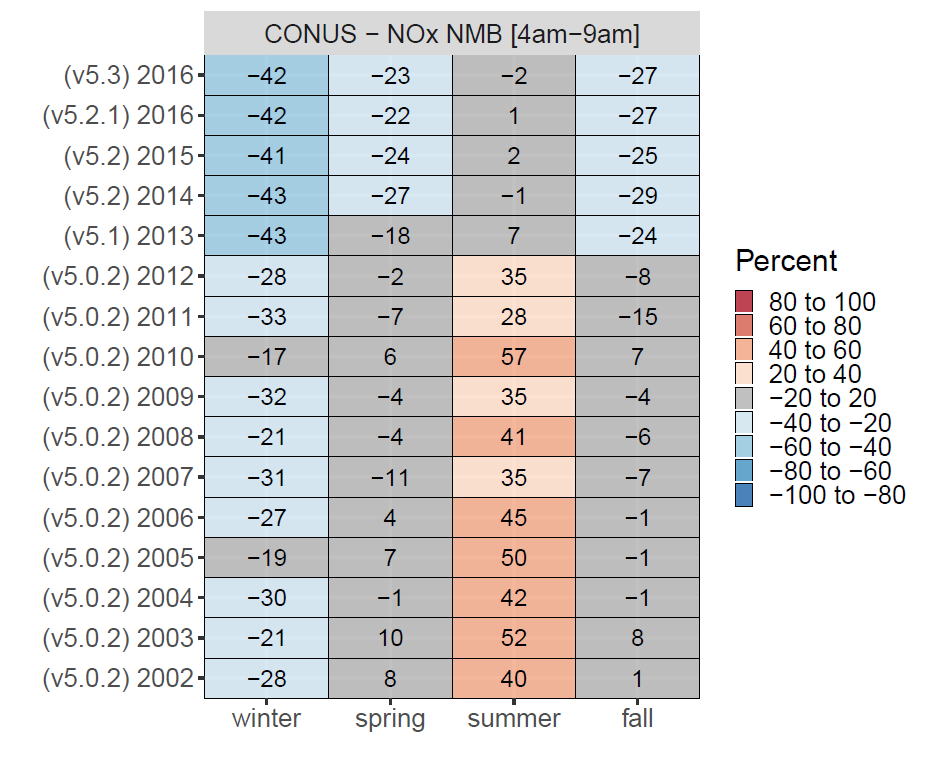


**Figure S4.** **Normalized Mean Bias (NMB) of morning modeled NO_X_ and observed NO_X_**_._ Morning hours are 4 – 9 a.m. LST. Data have been aggregated by season for each annual simulation across all monitors in US domain (Fig. S1).


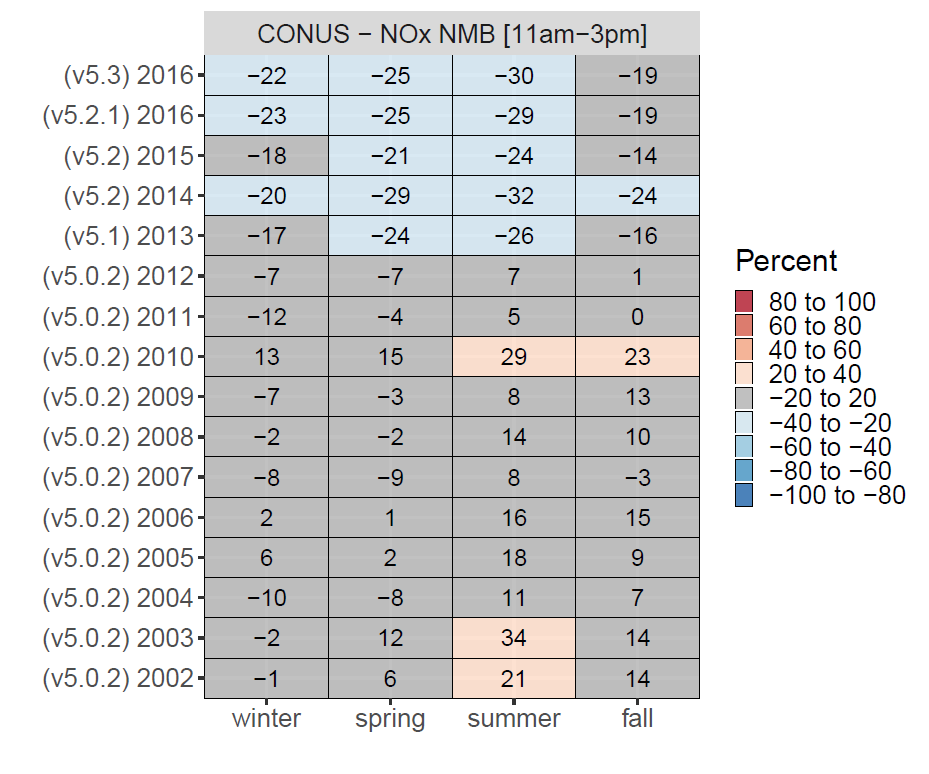


**Figure S5.** **Normalized Mean Bias (NMB) of midday modeled NO_X_ and observed NO_X_**_._ Midday hours are 11 a.m. – 3 p.m. LST. Data have been aggregated by season for each annual simulation across all monitors in US domain (Fig. S1).


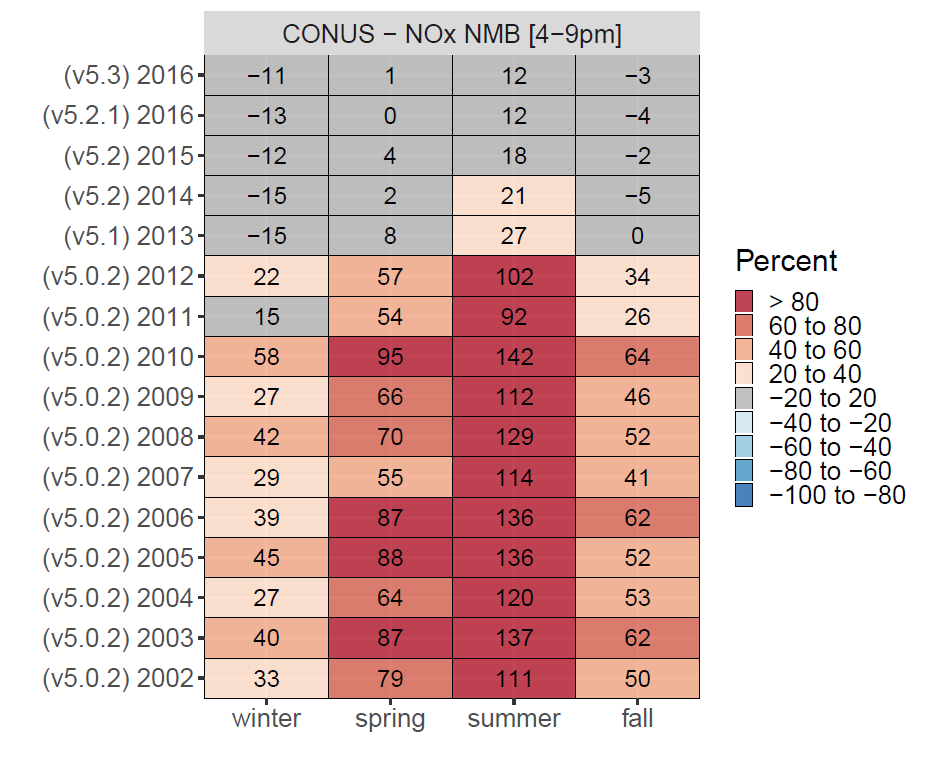


**Figure S6.** **Normalized Mean Bias (NMB) of evening modeled NO_X_ and observed NO_X_**_._ Evening hours are 4 p.m. – 9 p.m. LST. Data have been aggregated by season for each annual simulation across all monitors in US domain (Fig. S1).


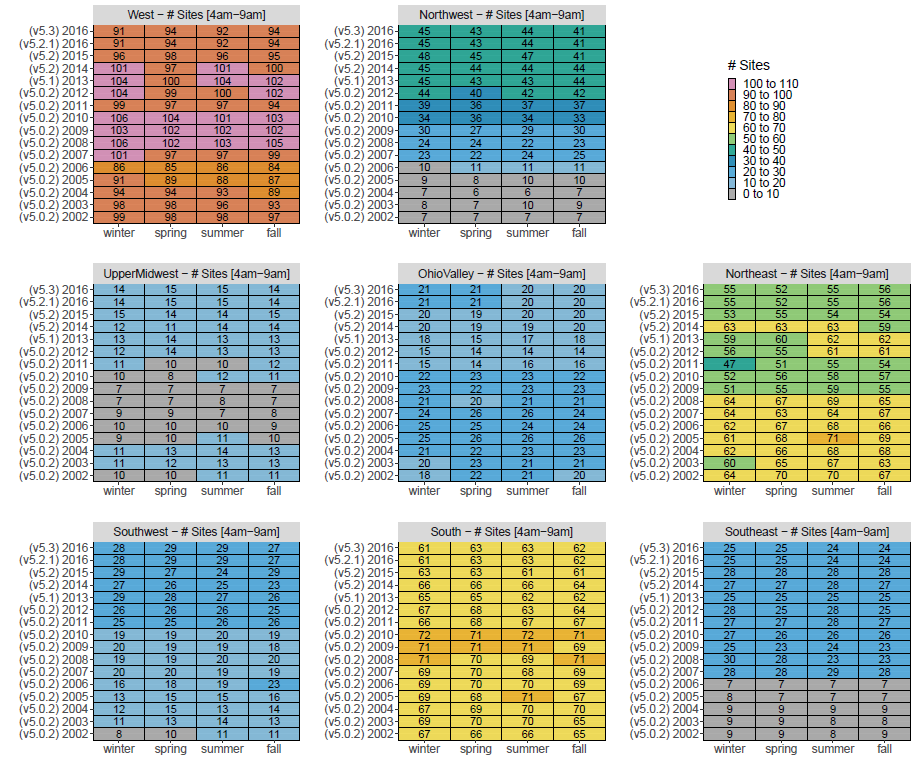


**Figure S7.** **Number of sites used to calculate Normalized Mean Bias of morning NO_X_ in Figure 5.** Morning hours considered are 4-9 a.m. LST. NMB is shown by region and year/season. West = CA, NV; Northwest = OR, WA, ID, MT, NE, ND, SD, WY; Upper Midwest = IA, MI, MN, WI; Ohio Valley = IL, IN, KY, MO, OH, TN, WV; Northeast = CT, DE, ME, MD, MA, NH, MJ, NY, PA, RI, VT; Southwest = AZ, CO, NM, UT; South = AR, KS, LA, MS, OK, TX; Southeast = AL, FL, GA, NC, SC, VA


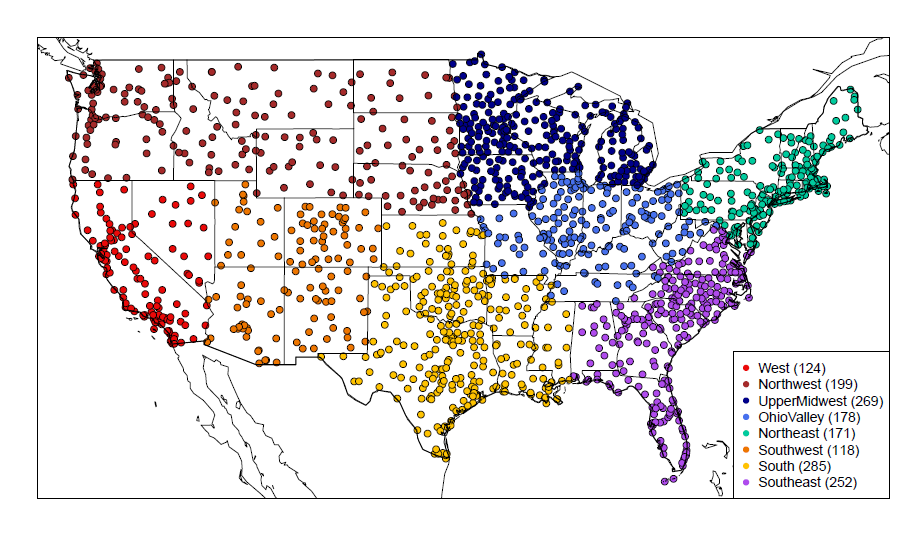
**Figure S8. METAR stations.** Weather stations used to evaluate the WRF meteorological model in Figures S9 – S11.


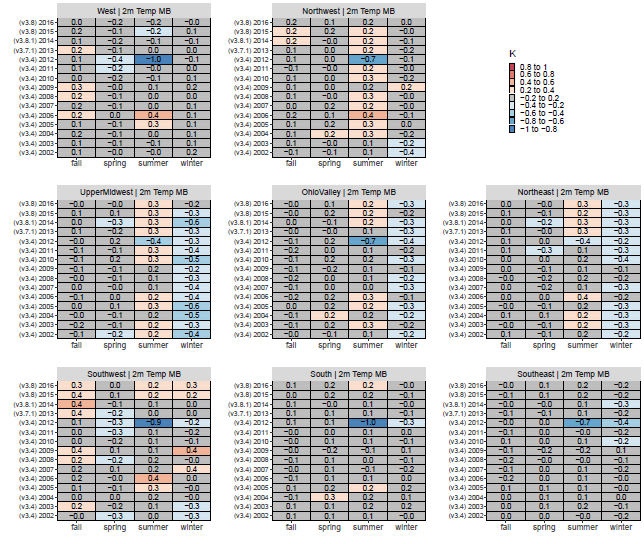


**Figure S9.** **Mean Bias (MB) of 2 meters temperature.** Data have been aggregated by season for each annual simulation across all METAR stations in US domain (Fig. S8).


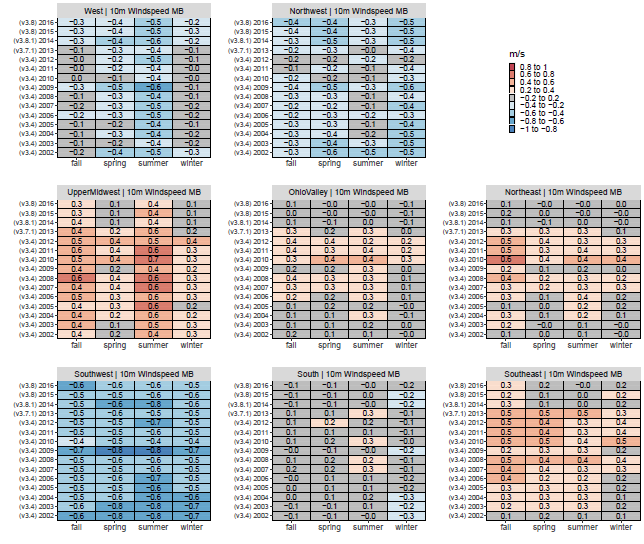


**Figure S10.** **Mean Bias (MB) of 10 meters windspeed.** Data have been aggregated by season for each annual simulation across all METAR stations in US domain (Fig. S8).


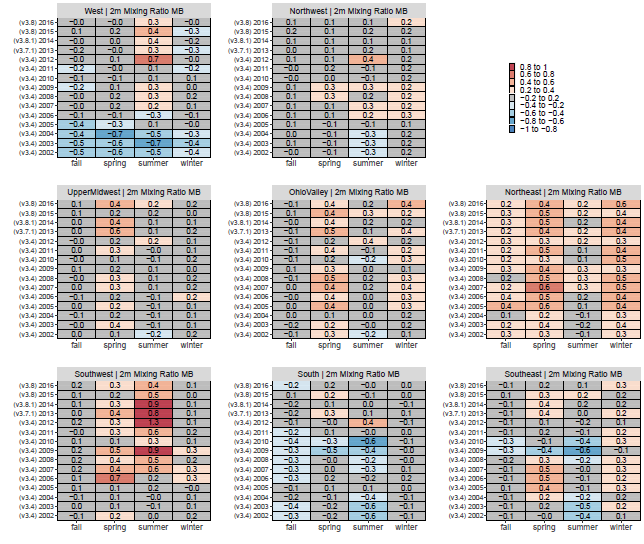


**Figure S11.** **Mean Bias (MB) of 2 meters water vapor mixing ratio.** Water vapor mixing ratio is a measure of the moisture in the air and is approximately equal to specific humidity. Data have been aggregated by season for each annual simulation across all METAR stations in US domain (Fig. S8).


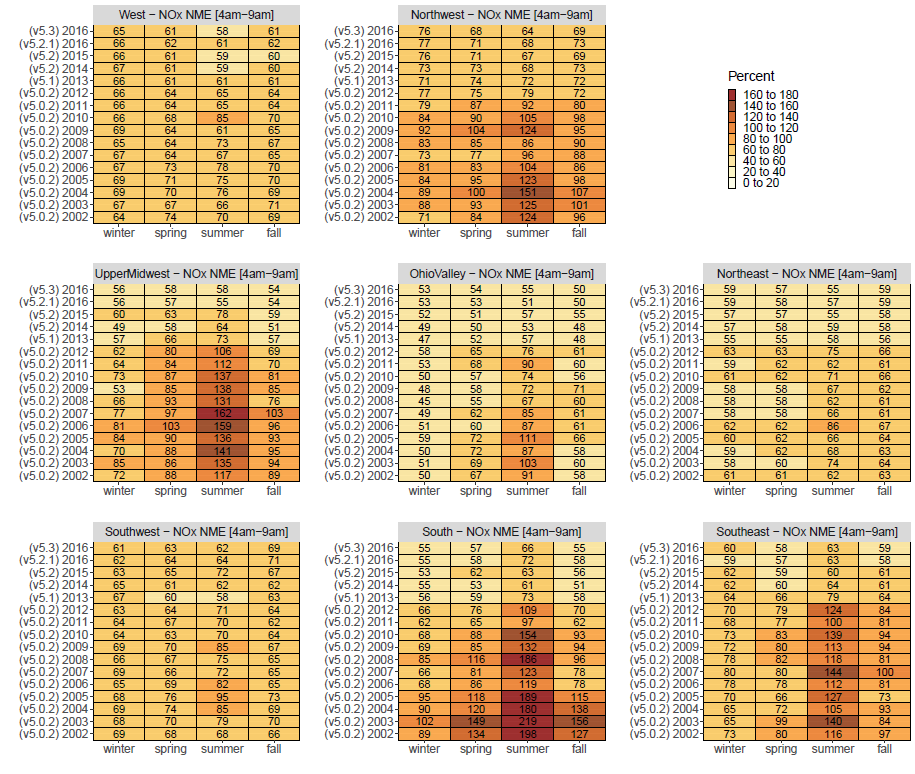


**Figure S12.** **Normalized Mean Error (NME) of morning modeled NO_X_ and observed NO_X_**_._ Morning hours are 4-9 a.m. LST. Data has been aggregated by season for each annual simulation across monitors in multiple regions defined by NOAA climate region (Fig. S1). West = CA, NV; Northwest = OR, WA, ID, MT, NE, ND, SD, WY; Upper Midwest = IA, MI, MN, WI; Ohio Valley = IL, IN, KY, MO, OH, TN, WV; Northeast = CT, DE, ME, MD, MA, NH, MJ, NY, PA, RI, VT; Southwest = AZ, CO, NM, UT; South = AR, KS, LA, MS, OK, TX; Southeast = AL, FL, GA, NC, SC, VA


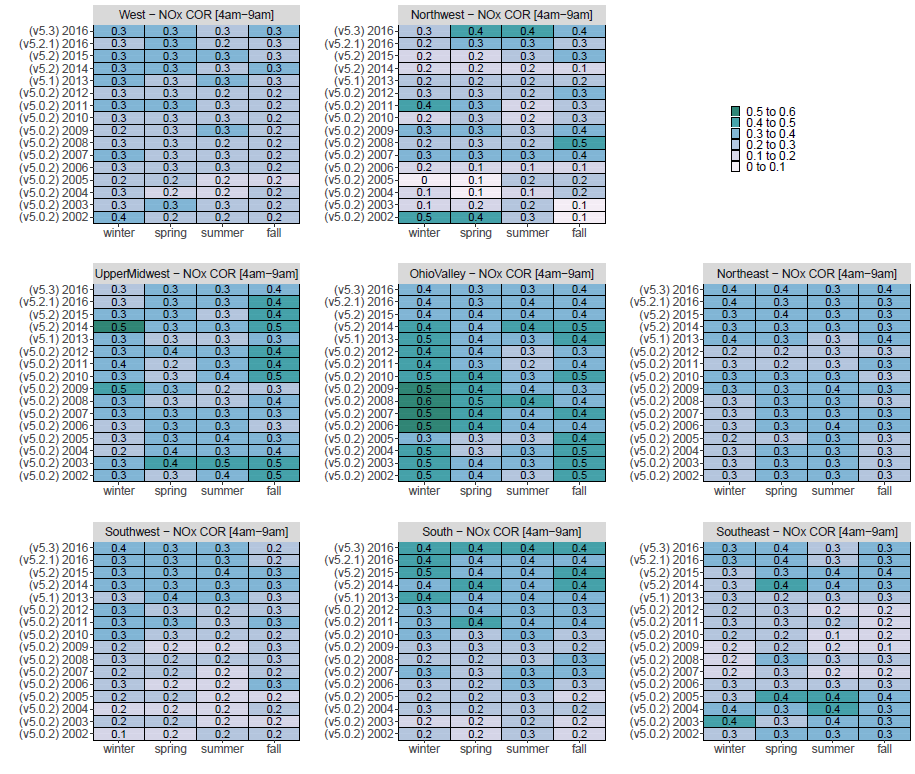


**Figure S13**. **Correlation (R^2^) of morning modeled NO_X_ and observed NO_X_**_._ Morning hours are 4-9 a.m. LST. Data has been aggregated by season for each annual simulation across monitors in multiple regions defined by NOAA climate region (Fig. S1). West = CA, NV; Northwest = OR, WA, ID, MT, NE, ND, SD, WY; Upper Midwest = IA, MI, MN, WI; Ohio Valley = IL, IN, KY, MO, OH, TN, WV; Northeast = CT, DE, ME, MD, MA, NH, MJ, NY, PA, RI, VT; Southwest = AZ, CO, NM, UT; South = AR, KS, LA, MS, OK, TX; Southeast = AL, FL, GA, NC, SC, VA


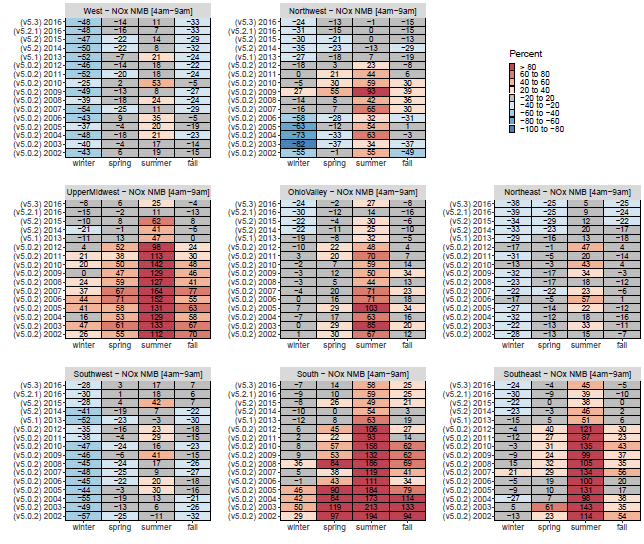


**Figure S14. As in Figure 5 of the main paper, but for modeled NO_Y_ – observed NO_X_**


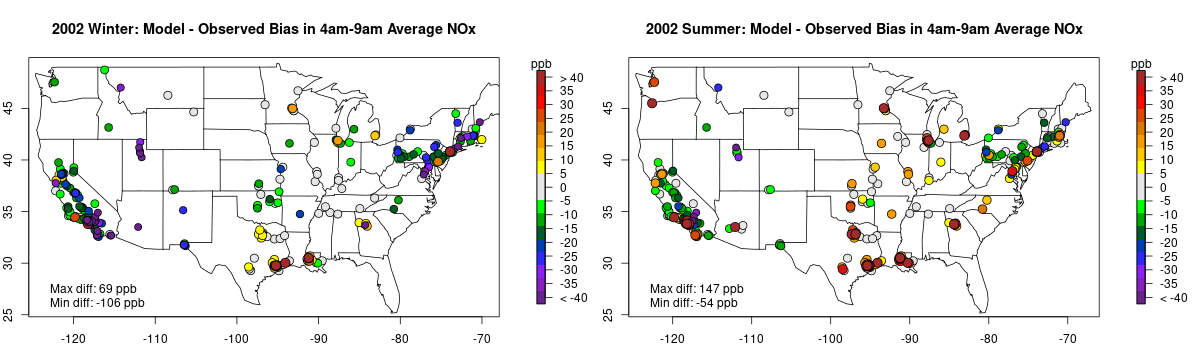


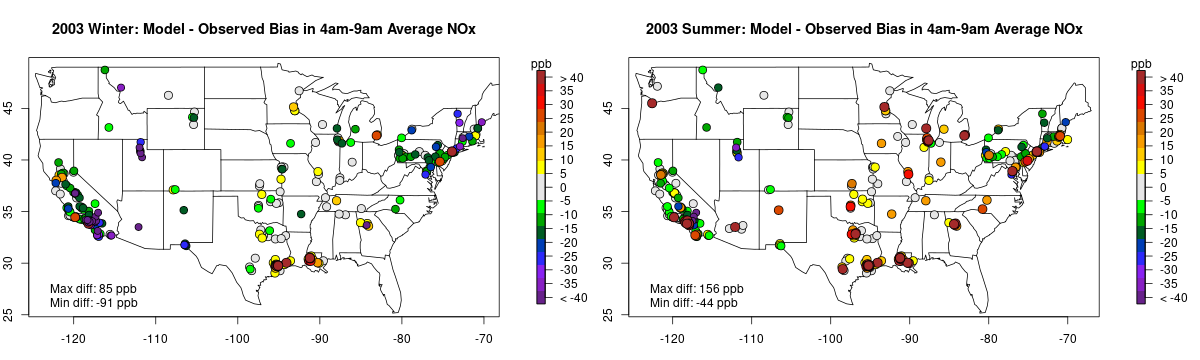

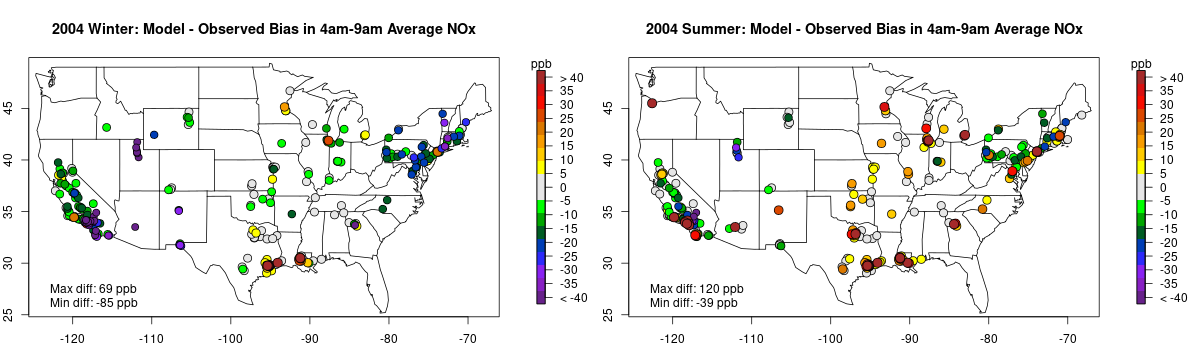

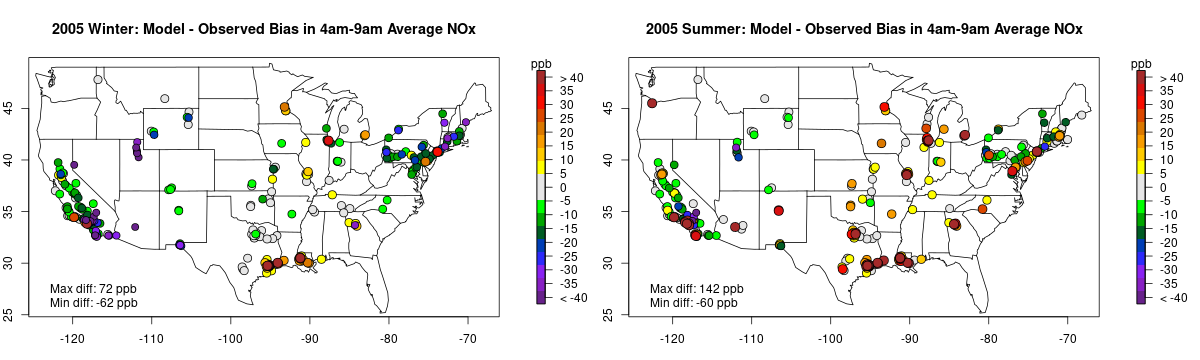


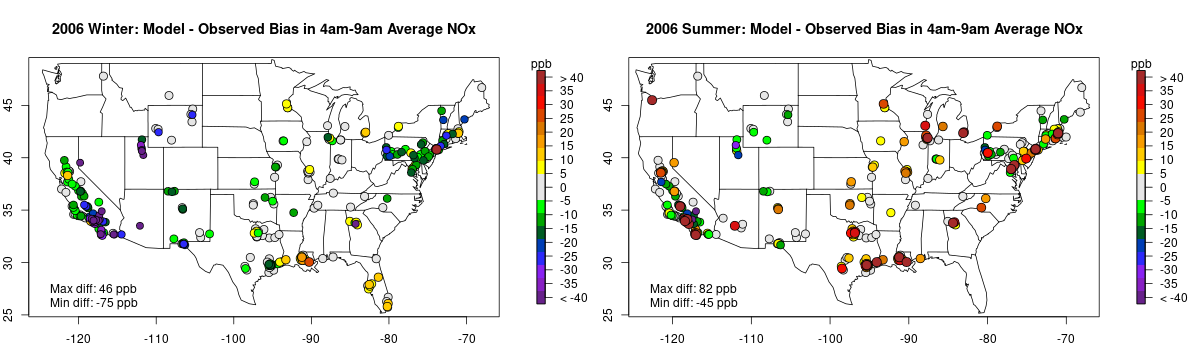

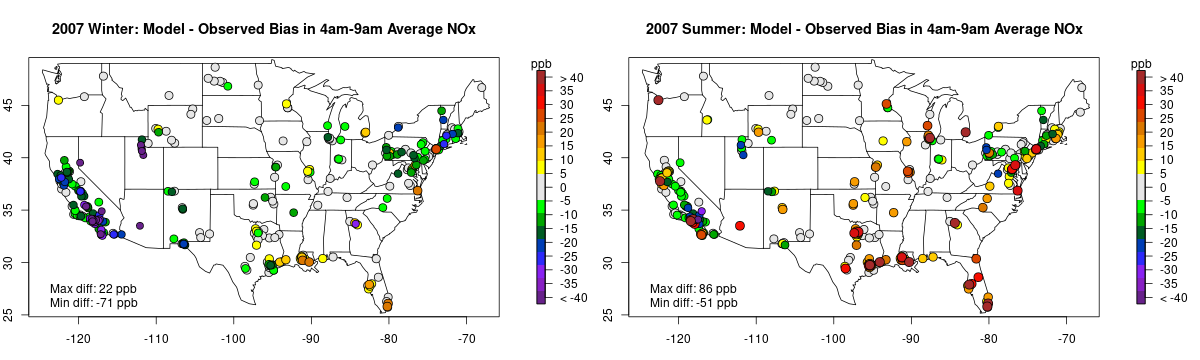

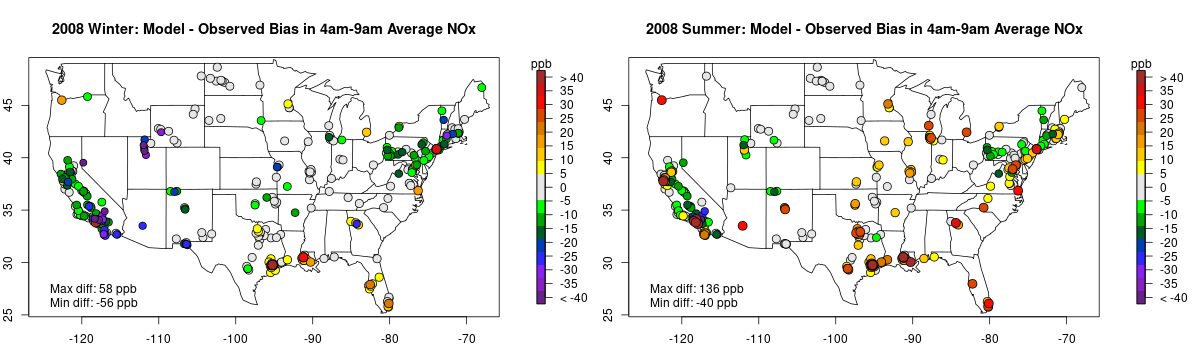

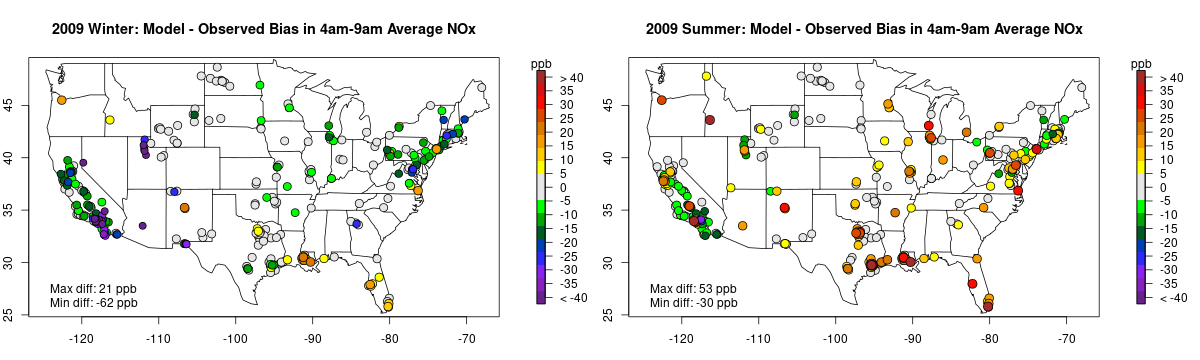

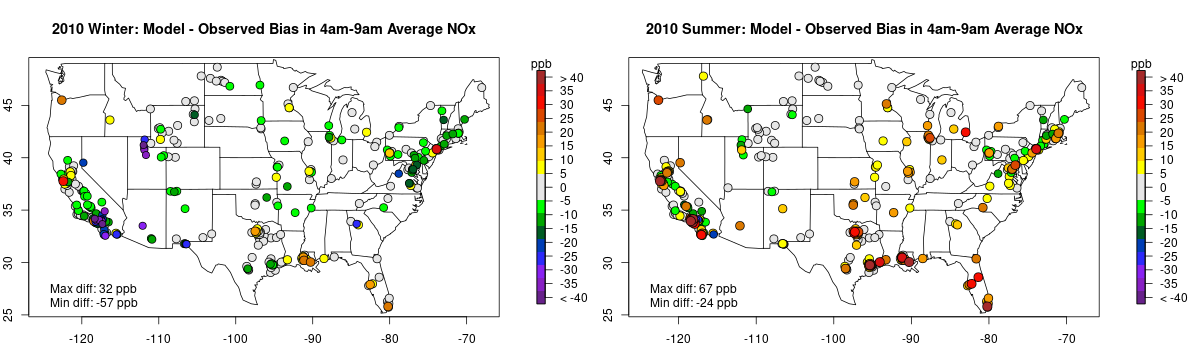

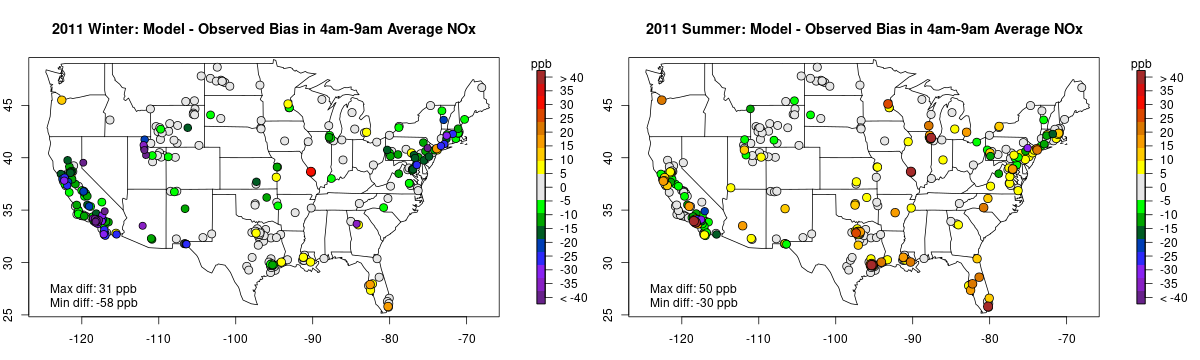

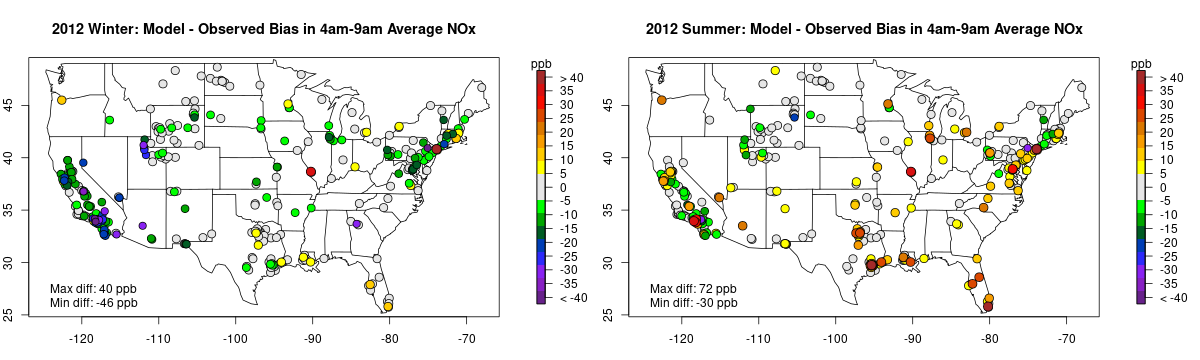

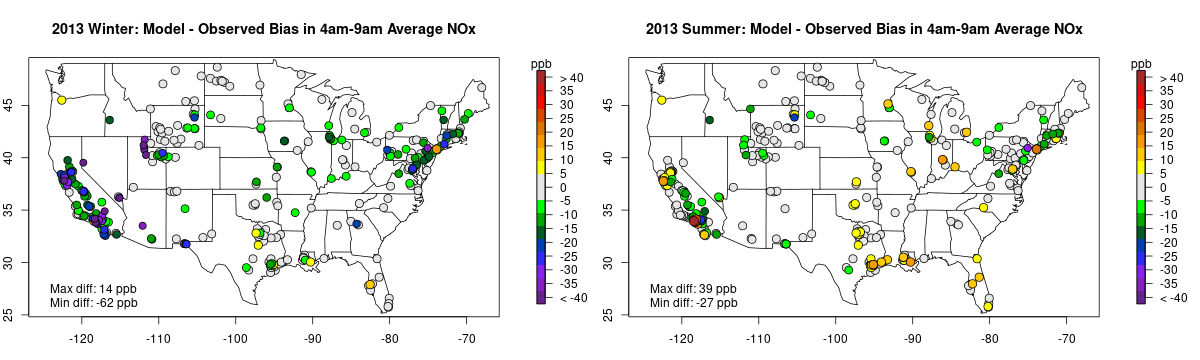

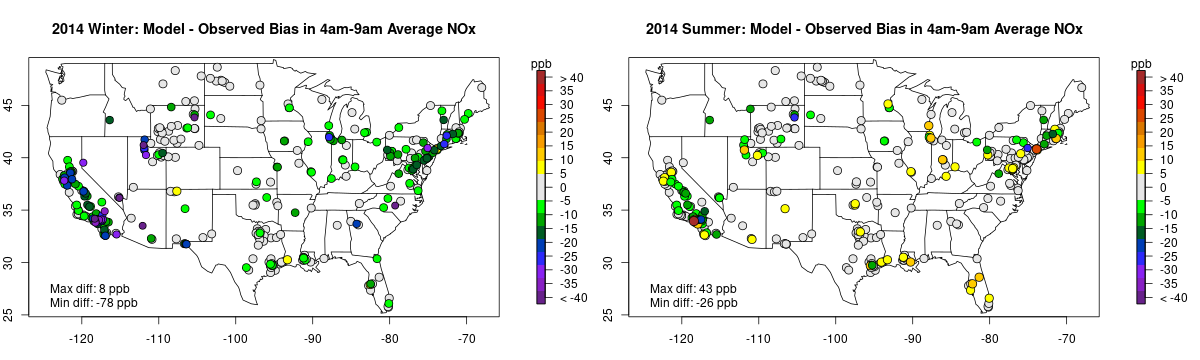


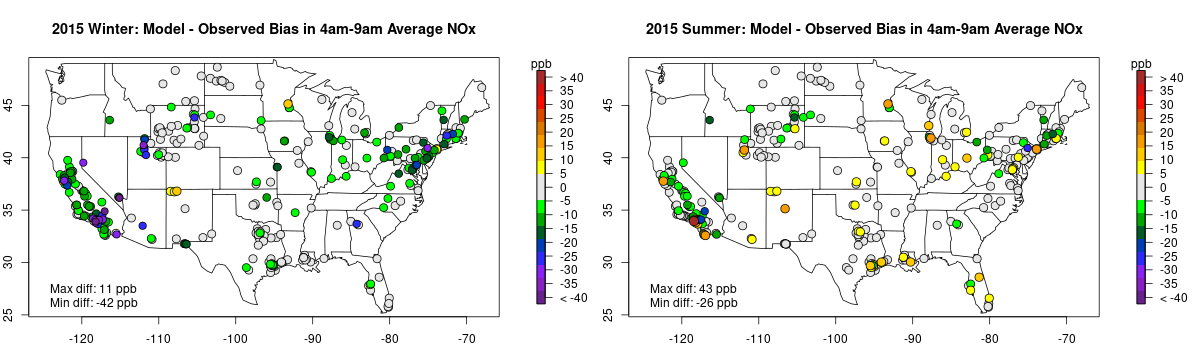


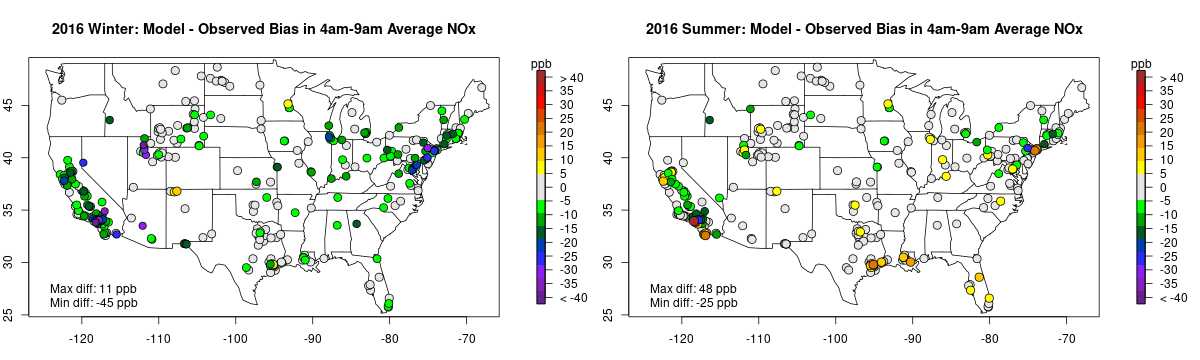

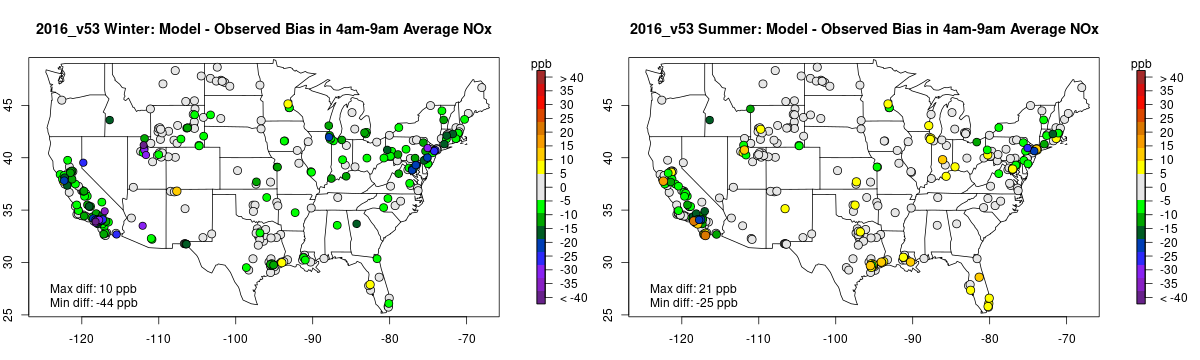


**Figure S15.** **Mean bias of morning Modeled NO_X_ and Observed NO_X_ at surface monitors for 2002–2016**. Morning hours are 4-9 a.m. LST. Data has been aggregated for winter (left column) and summer (right column) months. Warm colors indicate model over-prediction and cool colors under-prediction.

## Emissions Sensitivities

We apply three model sensitivity simulations to test the impact of various emissions updates. Model output from these simulations were compared against the 2011 simulation described in Table 1 in the main article. Evaluation of these sensitivities focused on the Northeastern US during July 2011 to include locations monitored during the DISCOVER-AQ Baltimore field campaign which has served as a valuable and widely used dataset for model NOx evaluation studies (Anderson et al., 2014; Reed et al., 2015; Zhang et al., 2016; Lee et al., 2018; Simon et al., 2018; Kang et al., 2019).

Emissions sensitivities were conducted for July 2011 that include updated equipment population information and temporal profiles for the nonroad emissions sector based on the updates released in the Nonroad component of MOVES2014b (Figure S16), more realistic temporal profiles of heavy-duty onroad emissions for some urban areas in the eastern U.S. (Figure S17) based on data from the Vehicle Travel Information Systems (VTRIS) (Federal Highway Administration, 2011), and better temporal allocation of EGUs (Figure S18). Sensitivities used perturbations of sectors with large NO_X_ contribution to emissions in the northeast U.S. (Figure 2) to gauge the importance of properly characterizing the timing of these emissions. Each of these sensitivities resulted in reduced monthly average NO_X_ throughout the northeastern U.S. Downward adjustments to estimates of nonroad equipment population resulted in ~7% reduction in nonroad NO_X_ emissions nationally. The nonroad emissions changes along with updates to the temporal profiles of construction and lawn and garden equipment that shifted activity away from nighttime hours and toward daytime hours together resulted in some moderate reductions (up to 3.2 ppb) in monthly mean NO_X_ across many of the urbanized areas in the Northeast (Figure S19a). The timing of actual mobile activity may be slightly shifted in each of these areas since the modeling system typically uses a default temporal profile to allocate emissions to hour of the day. However, these urban areas often experience increased mobile activity during morning and evening commuting periods, so temporal mis-assignment may contribute to, but not fully explain this performance feature. A model sensitivity where heavy-duty onroad temporal profiles for some parts of the northeast corridor were updated with activity patterns that reflect differences in diesel vehicle behavior reduced model predicted NO_X_ by up to 0.5 ppb in some urban areas (Figure S19b) but did not substantively change model performance. Finally, there are several point sources that generate electricity that do not have Continuous Emissions Monitoring System (CEMS) data. In the base 2011 simulation, the hourly emissions for these sources were temporalized using regional average profiles from CEMS sources (by fuel type). The average regional profiles for fuel other than oil, gas, or coal had a large percentage of emissions concentrated on a few days and hours (i.e., they may have been used to generate electricity only during time periods when demand peaked). However, these profiles are inappropriate for sources such as municipal waste combustors and co-generation units, which do not operate in a “peaking” mode, so a sensitivity was performed for which the temporal profile for these sources was changed to a flat profile (same emissions for every day and hour). Modeled concentration changes due to the (non-CEMS) EGU sensitivity run were relatively small on most days (up to 1.2 ppb, Figure S19c), but NO_X_ reductions were large on several days in July, in areas close to municipal waste combustors, most notably on July 21^st^ and 22^nd^ (up to 7.6 ppb and 6.6 ppb respectively).


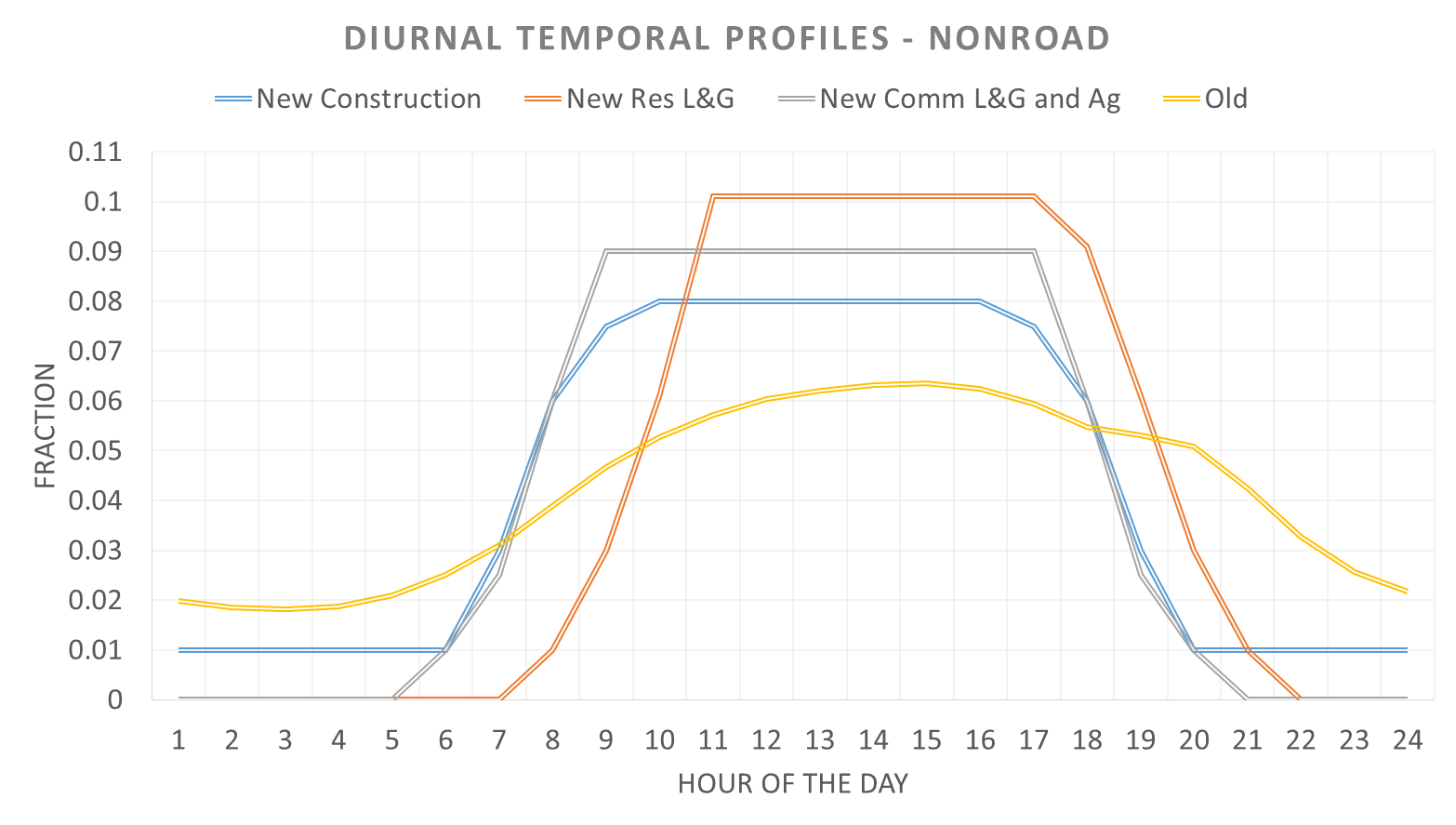


**Figure S16.** **Nonroad diurnal emissions profiles used in sensitivity test**. The 2011 base simulation (“old”) and sensitivity simulation are shown for the following sectors: construction equipment, residential lawn and garden equipment, commercial lawn and garden equipment and agricultural equipment.


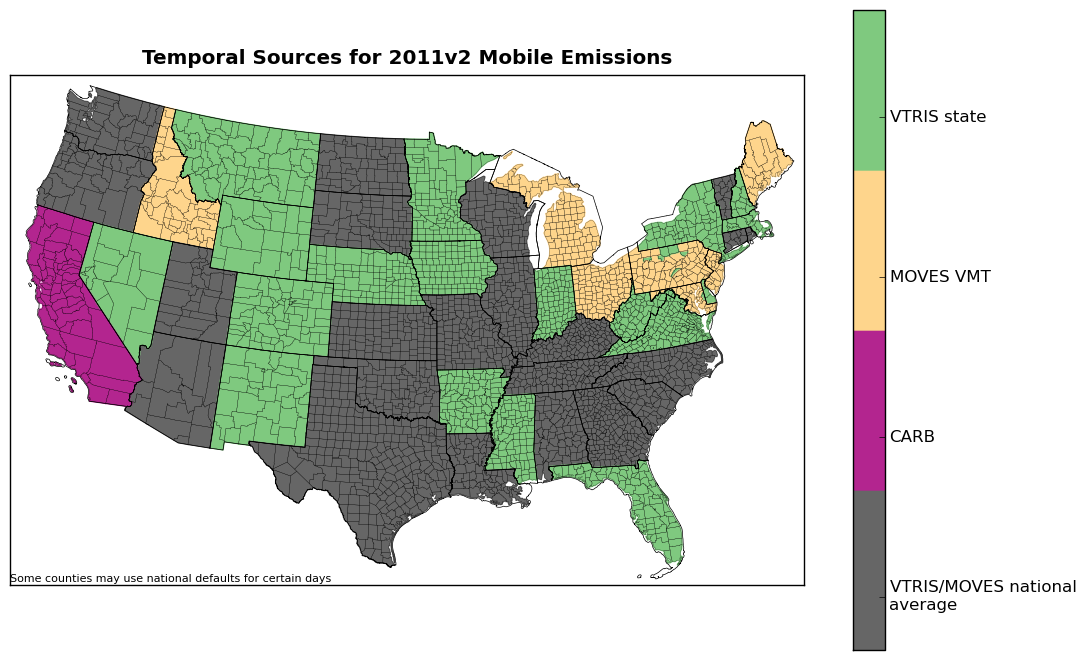


**Figure S17.** **Map of counties and parishes depicting source of temporal data.** Locations shown in grey indicate that EPA default data (derived from VTRIS) was used vs. state submitted data (green and yellow). Sensitivity run #2 replaced all state-submitted temporal profile data with EPA VTRIS derived profiles (except California). Source: (U.S. Environmental Protection Agency, 2016)


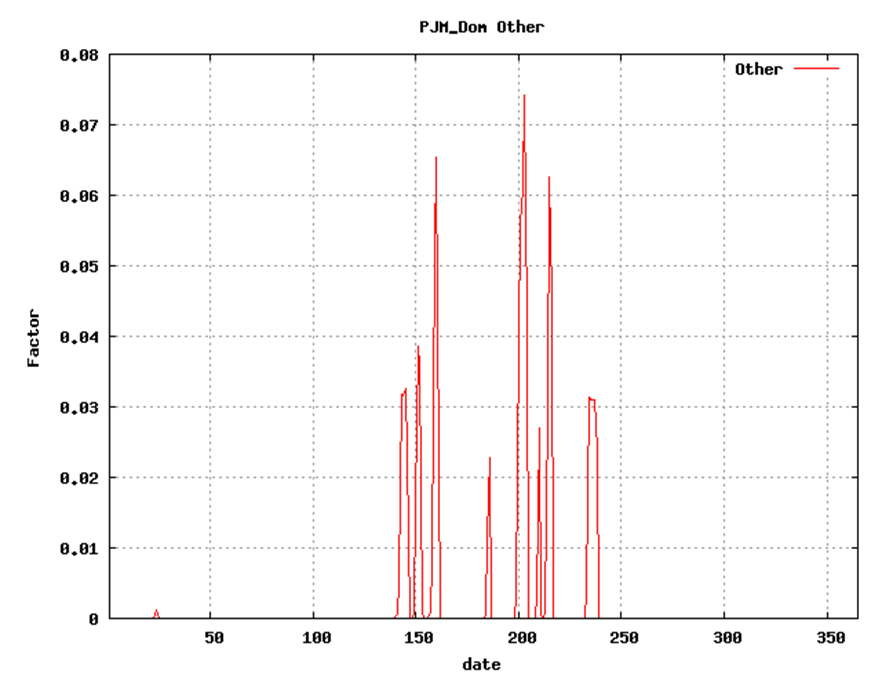


**Figure S18.** **Example day-of-year temporal profile for EGU sources (fuel = “other”) in Eastern Virginia**. X-axis provides Julian day for 2011. Y-axis provides fractional attribution of annual emissions to each day of the year. Up to 7% of the annual emissions are emitted on a single day.

**Figure S19.** **Change in the July 2011 average of modeled NO_X_ concentrations (ppb) resulting from sensitivity tests**. (a) nonroad emissions adjustments, (b) alternative heavy-duty onroad temporal profiles, (c) alternative temporal allocation of CEMs for year 2011 and d) CB6 chemical mechanism versus CB05 chemical mechanism (12-km horizontal grid resolution, all hours averaged).


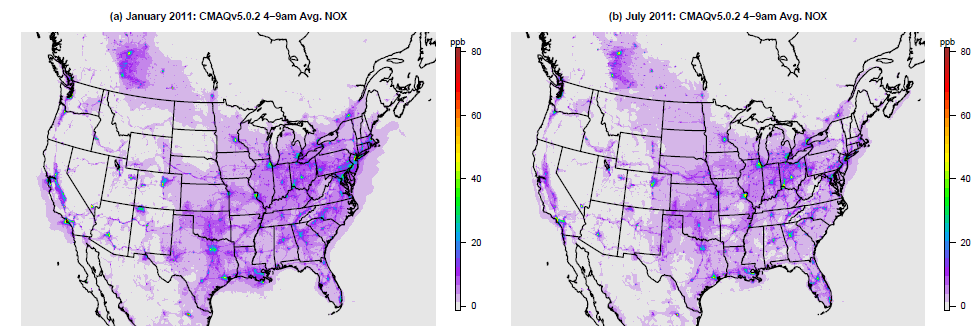

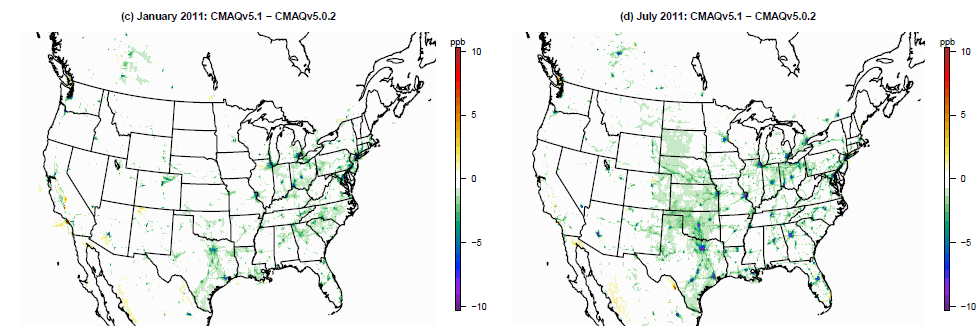

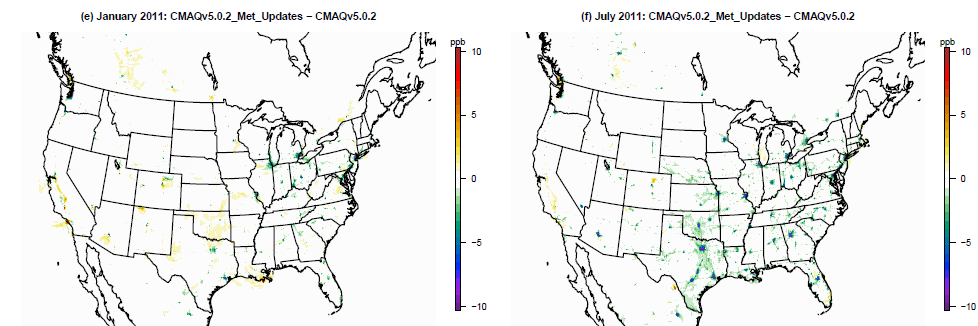

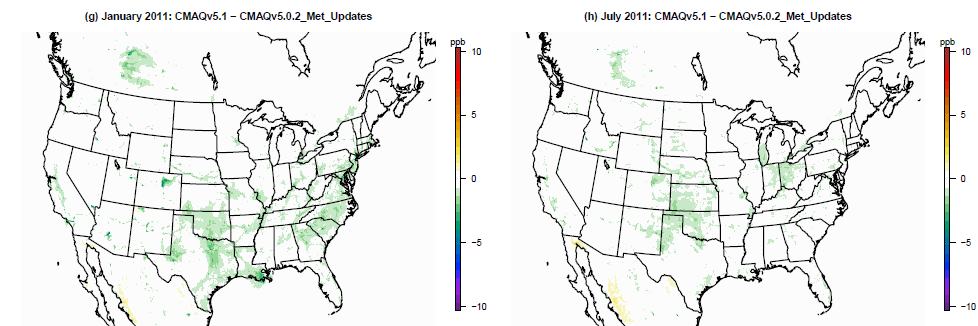


**Figure S20.** **Change in modeled NO_X_ mixing ratio (ppb) resulting from updating from CMAQv5.0.2 to CMAQv5.1.**  Maps show the spatial distribution of this change for (a) January 2011 average 4am – 9am LST NO_X_ from CMAQv5.0.2 simulation, (b) same as (a) but for July 2011, (c-d) difference between CMAQv5.1 and CMAQv5.0.2, (e-f) difference between meteorology sensitivity and CMAQv5.0.2, (g-h) difference between CMAQv5.1 and meteorology sensitivity.


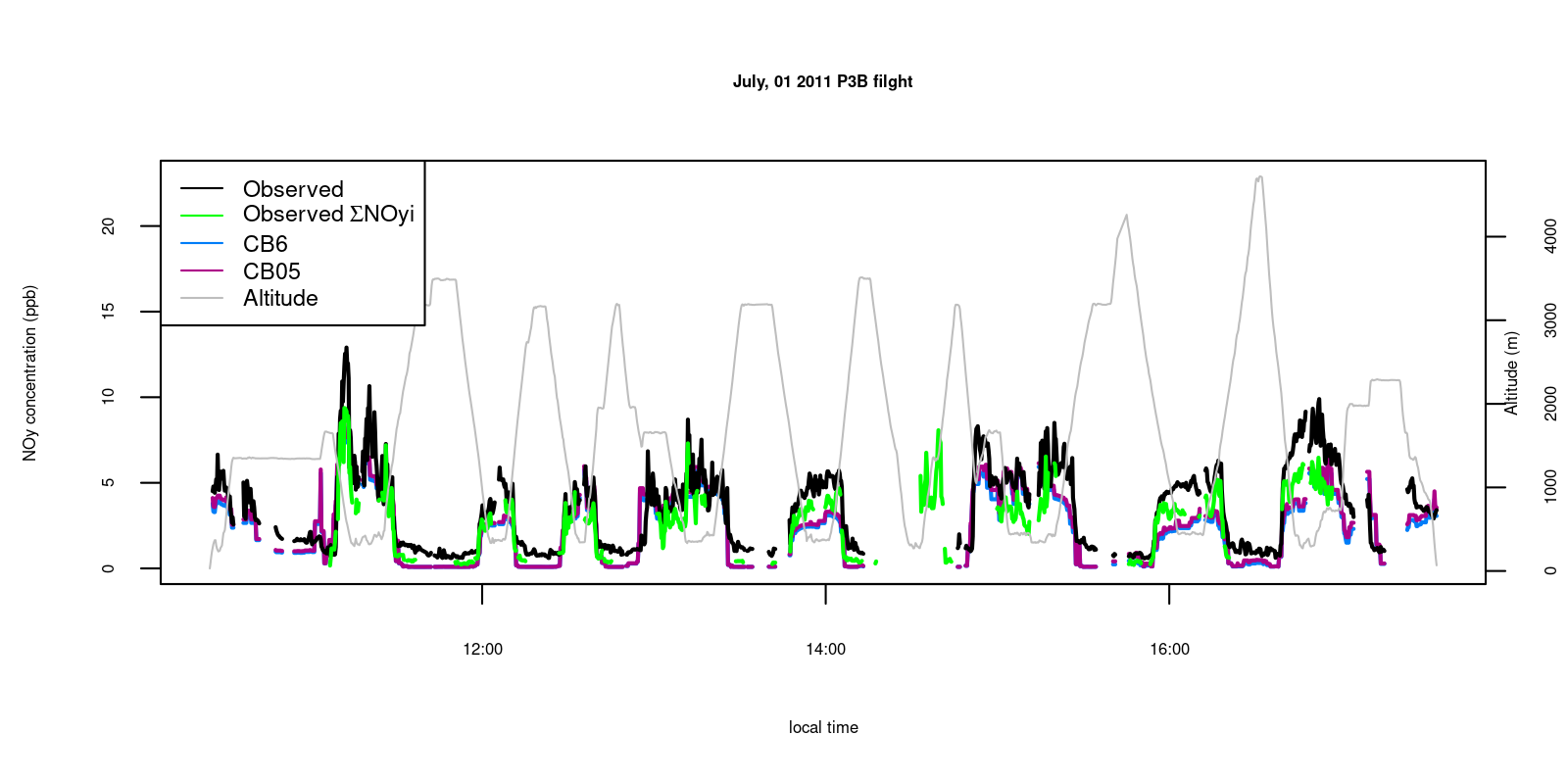


**Figure S21. Paired model and observed NOy from DISCOVER-AQ Baltimore flight on July 1, 2011.**


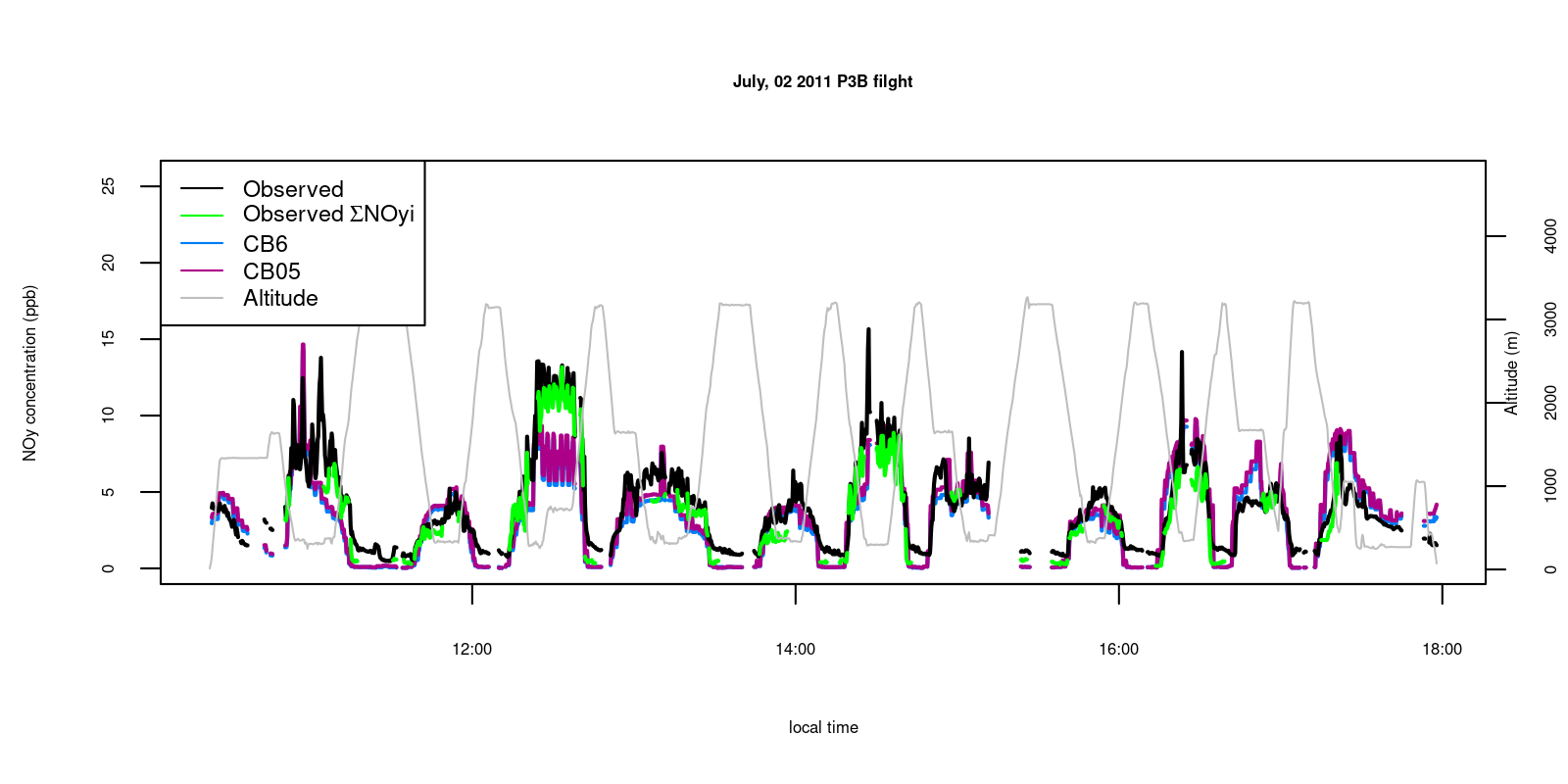


**Figure S22. Paired model and observed NOy from DISCOVER-AQ Baltimore flight on July 2, 2011.**


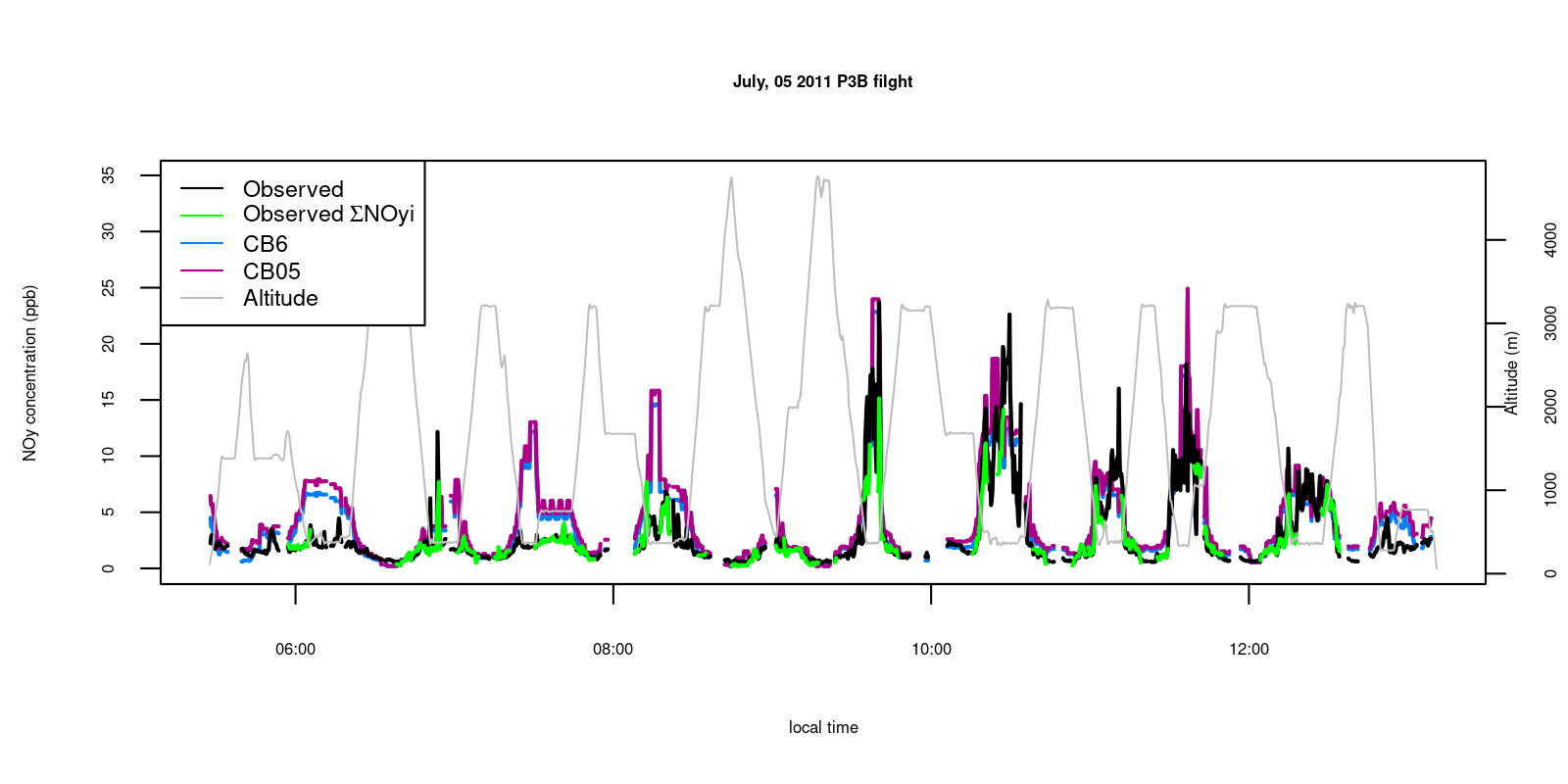


**Figure S23. Paired model and observed NOy from DISCOVER-AQ Baltimore flight on July 5, 2011.**


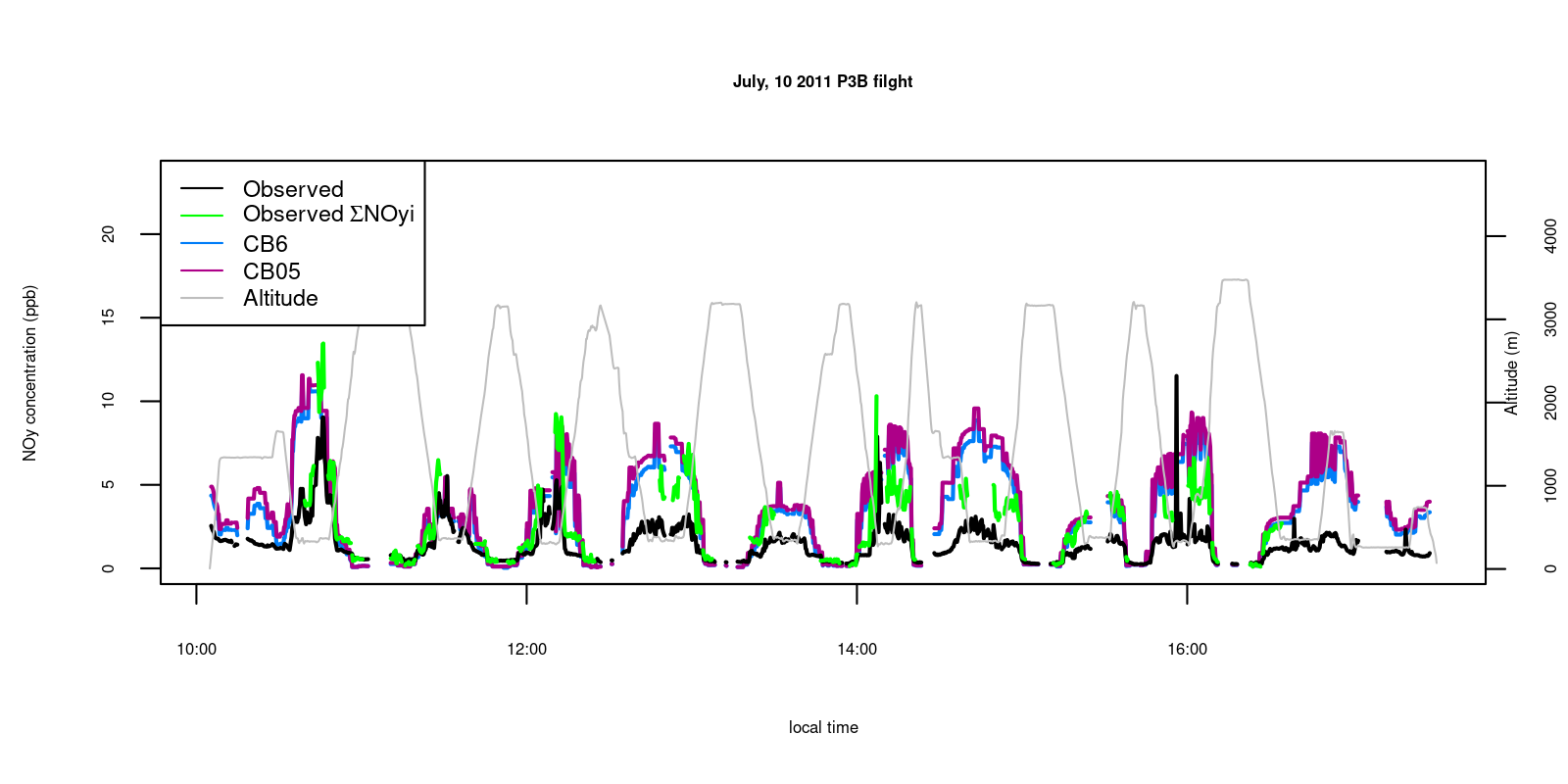


**Figure S24. Paired model and observed NOy from DISCOVER-AQ Baltimore flight on July 10, 2011.**


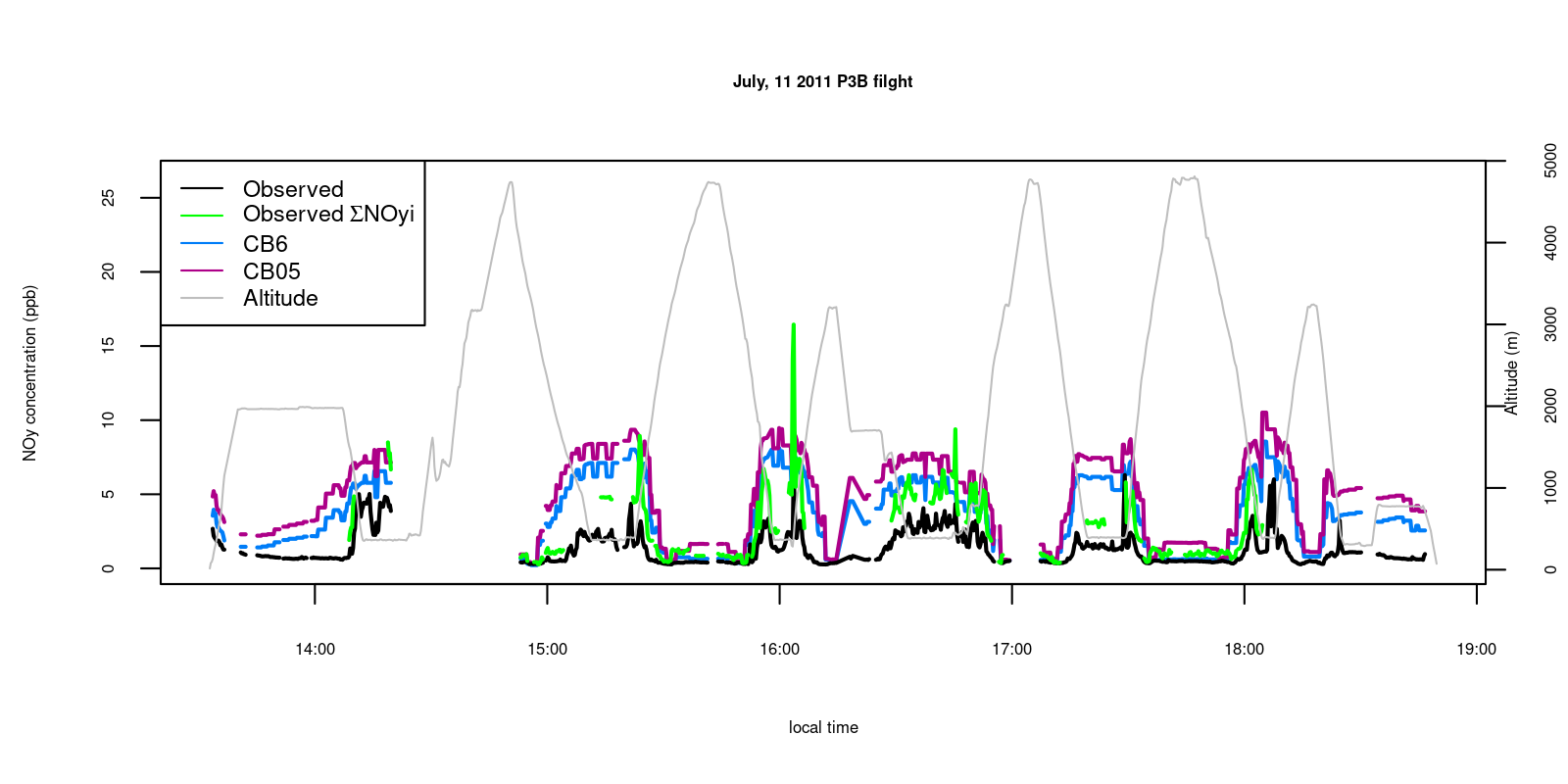


**Figure S25. Paired model and observed NOy from DISCOVER-AQ Baltimore flight on July 11, 2011.**


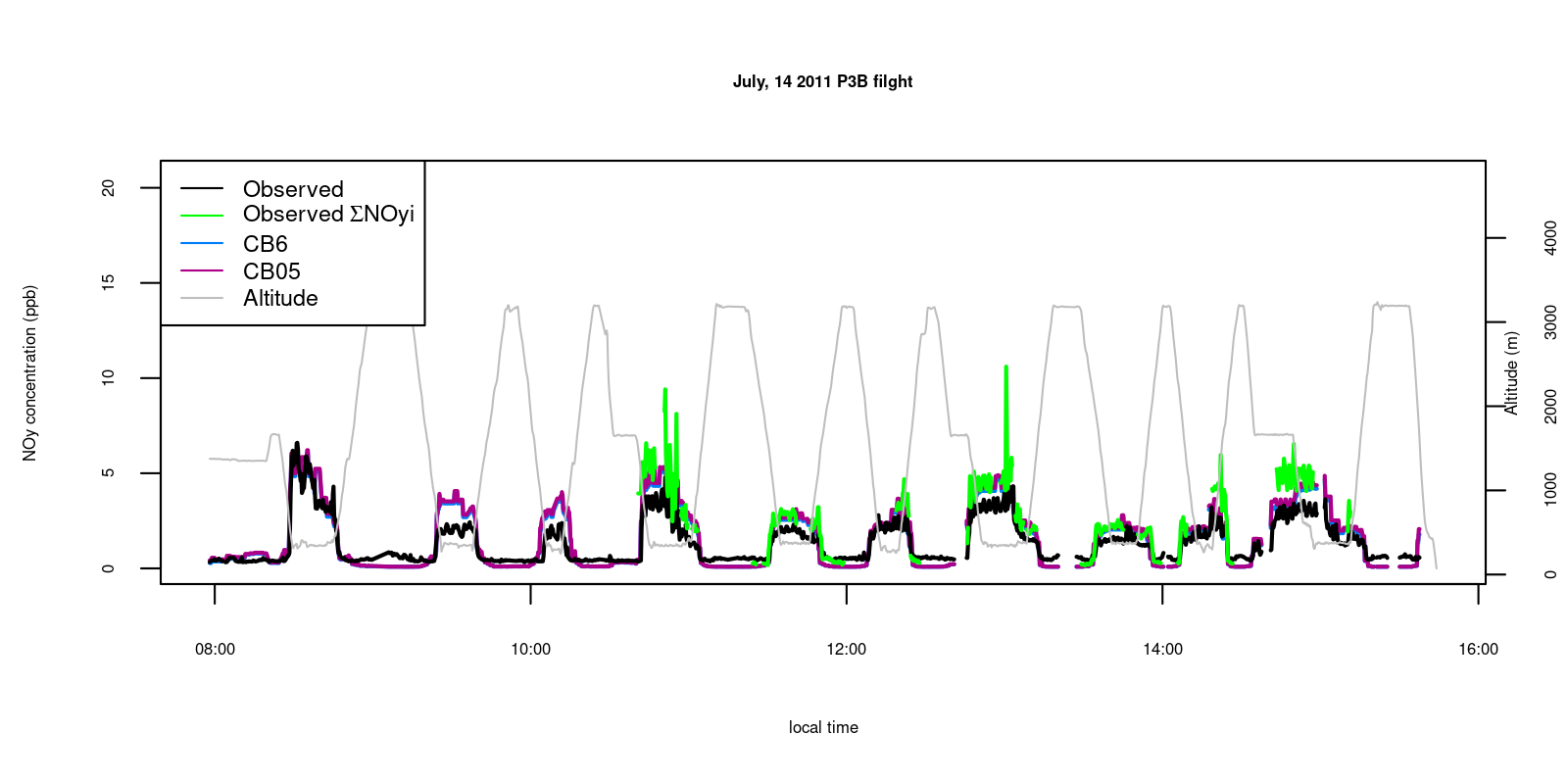


**Figure S26. Paired model and observed NOy from DISCOVER-AQ Baltimore flight on July 14, 2011.**


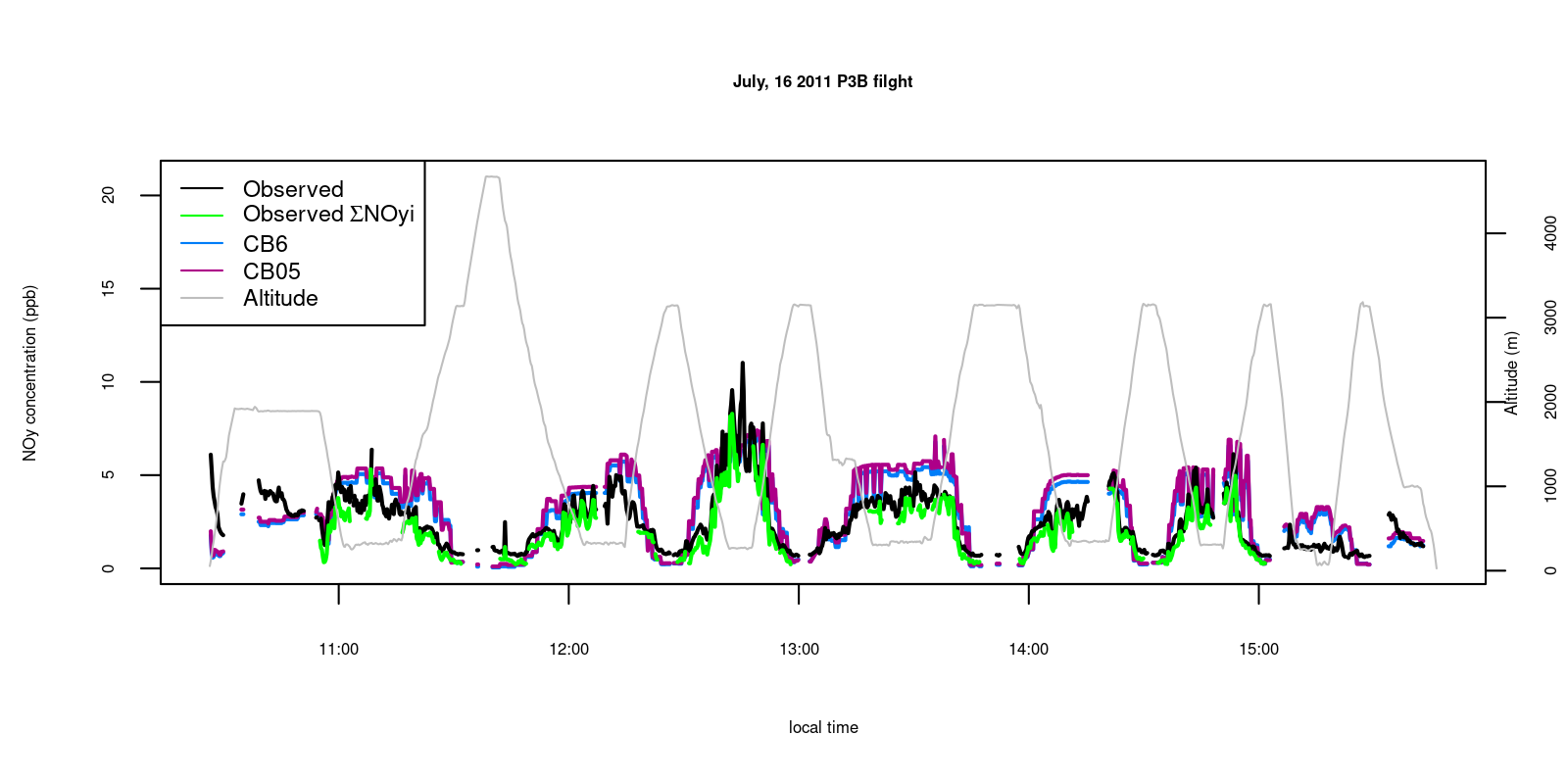


**Figure S27. Paired model and observed NOy from DISCOVER-AQ Baltimore flight on July 16, 2011.**


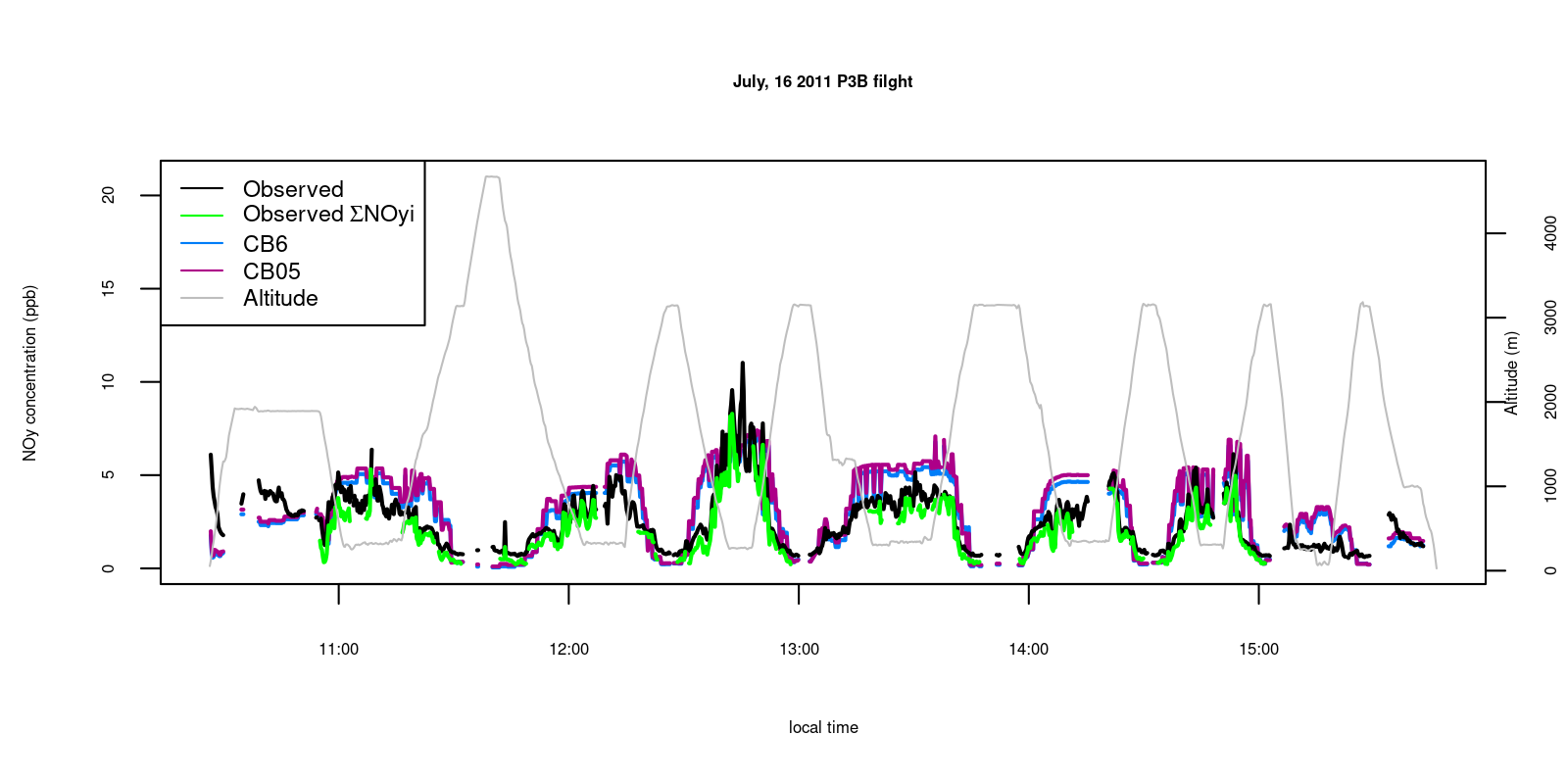


**Figure S28. Paired model and observed NOy from DISCOVER-AQ Baltimore flight on July 20, 2011.**


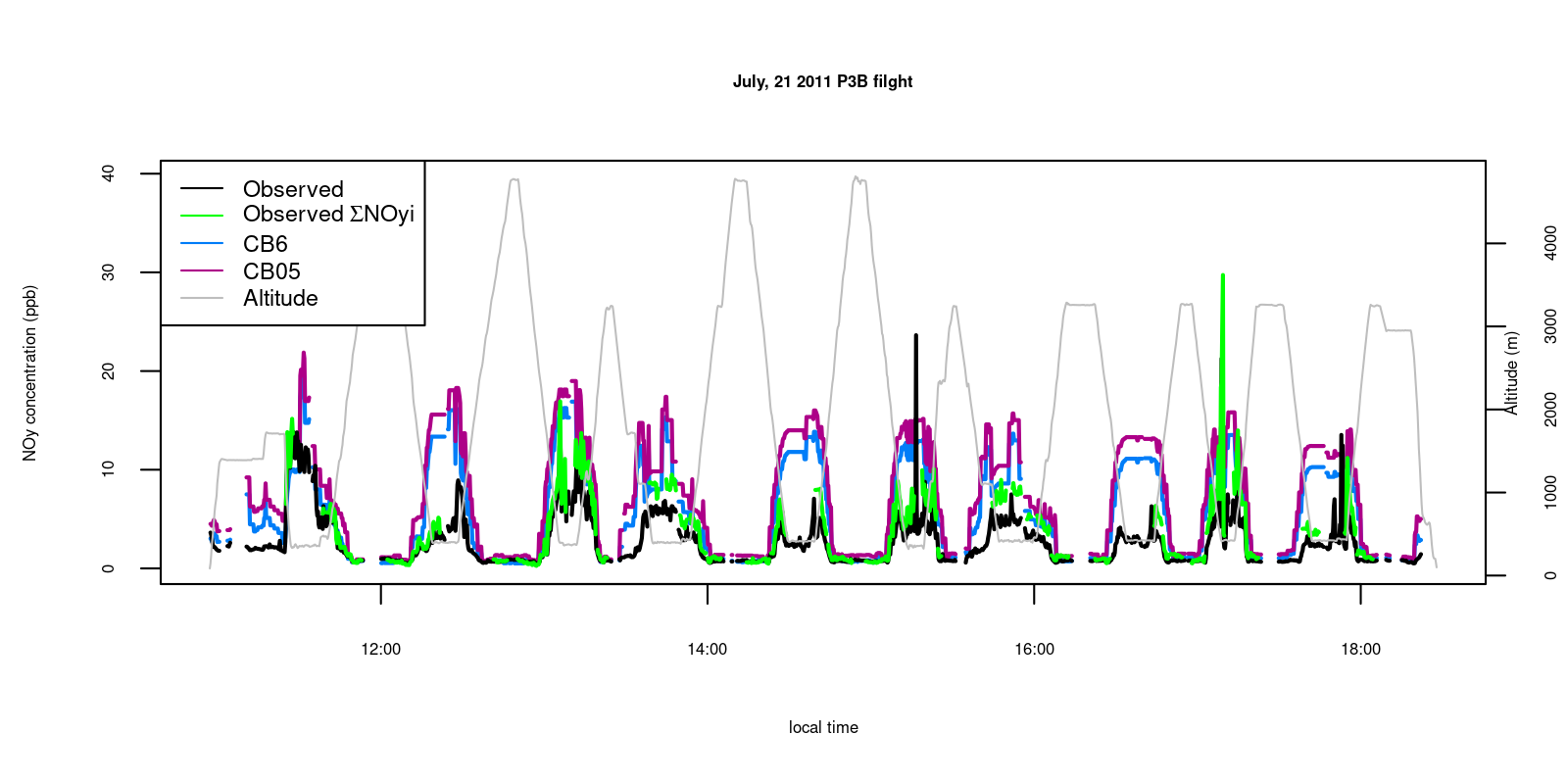


**Figure S29. Paired model and observed NOy from DISCOVER-AQ Baltimore flight on July 21, 2011.**


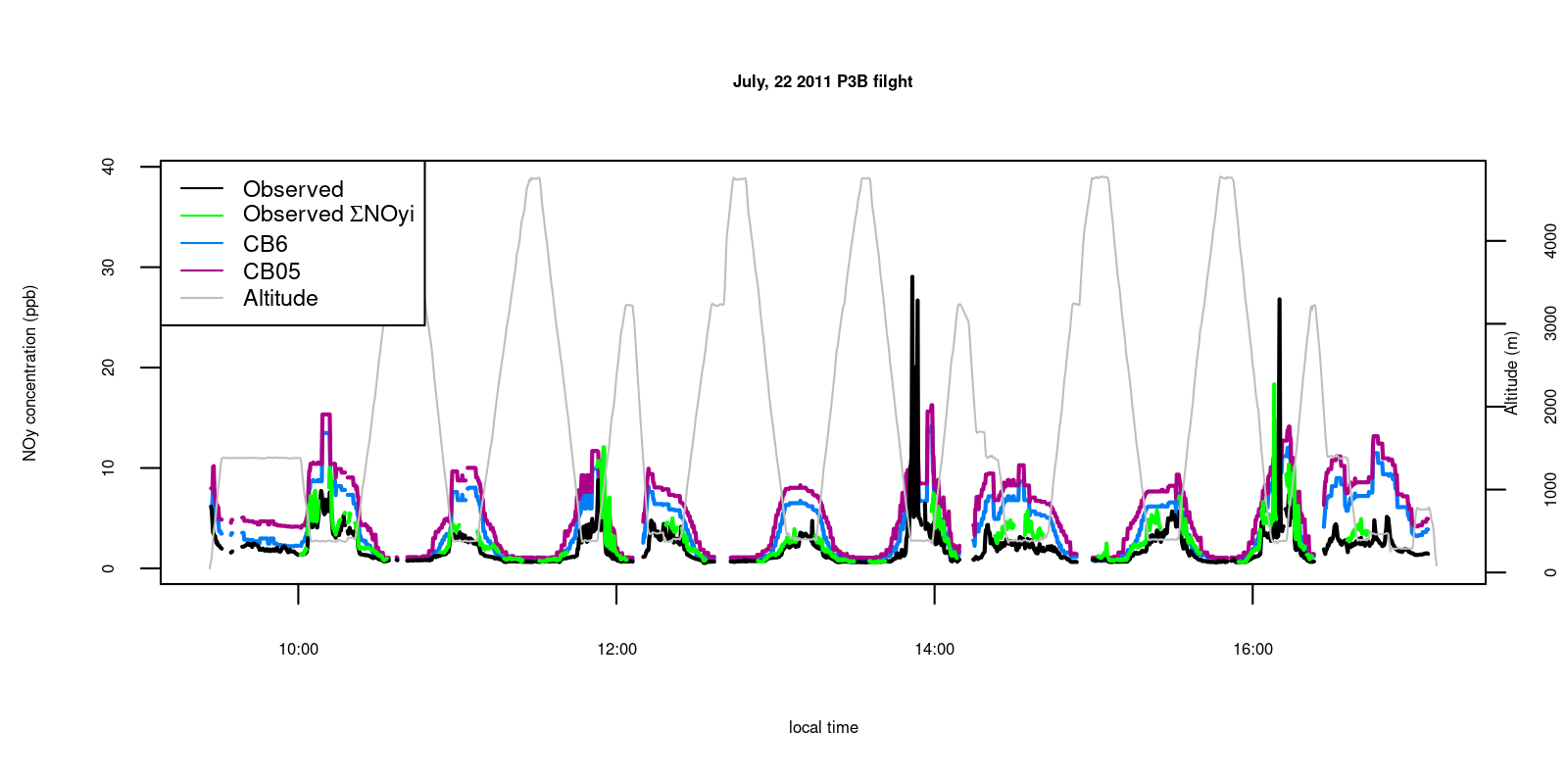


**Figure S30. Paired model and observed NOy from DISCOVER-AQ Baltimore flight on July 22, 2011.**


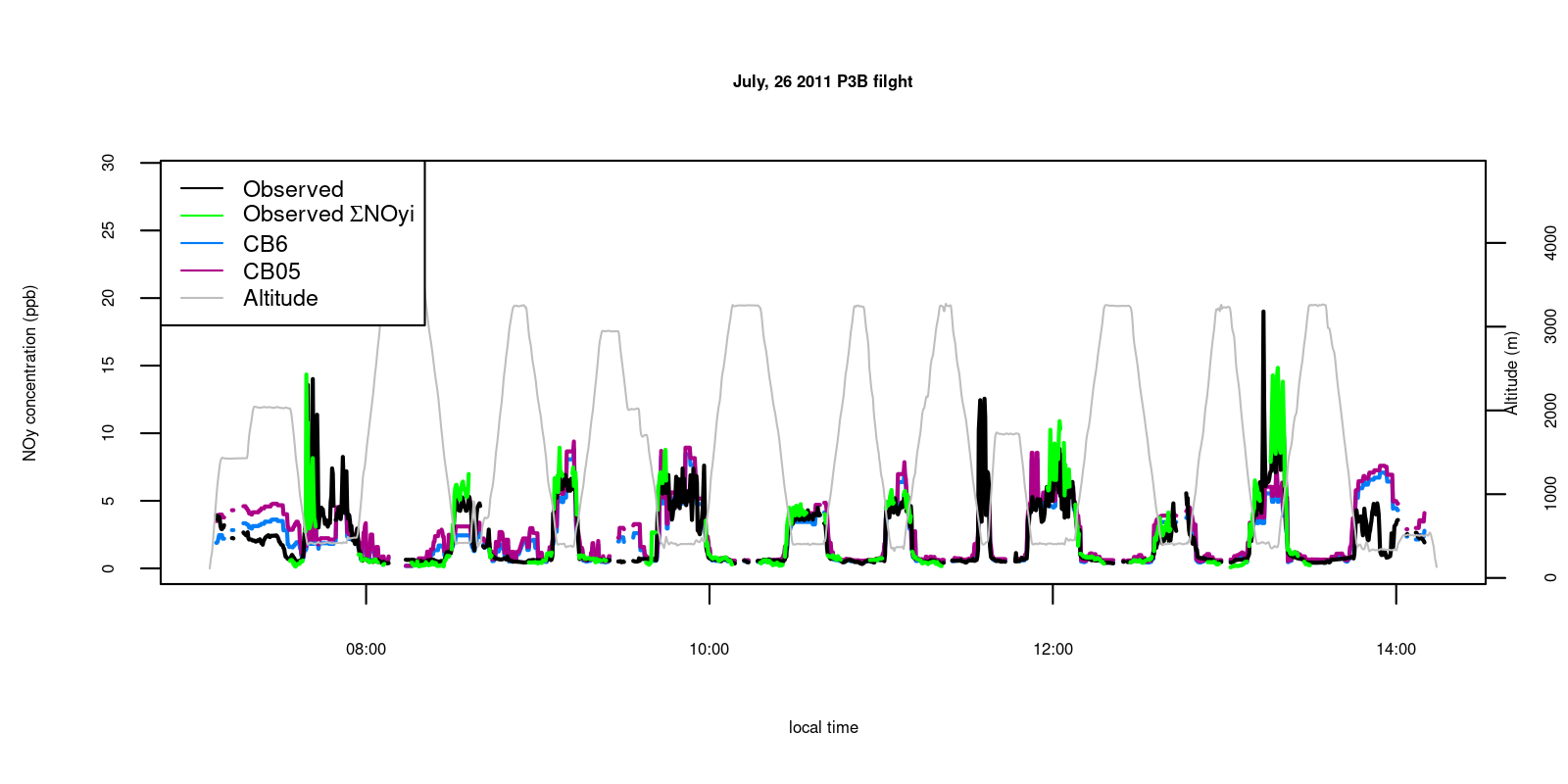


**Figure S31. Paired model and observed NOy from DISCOVER-AQ Baltimore flight on July 26, 2011.**


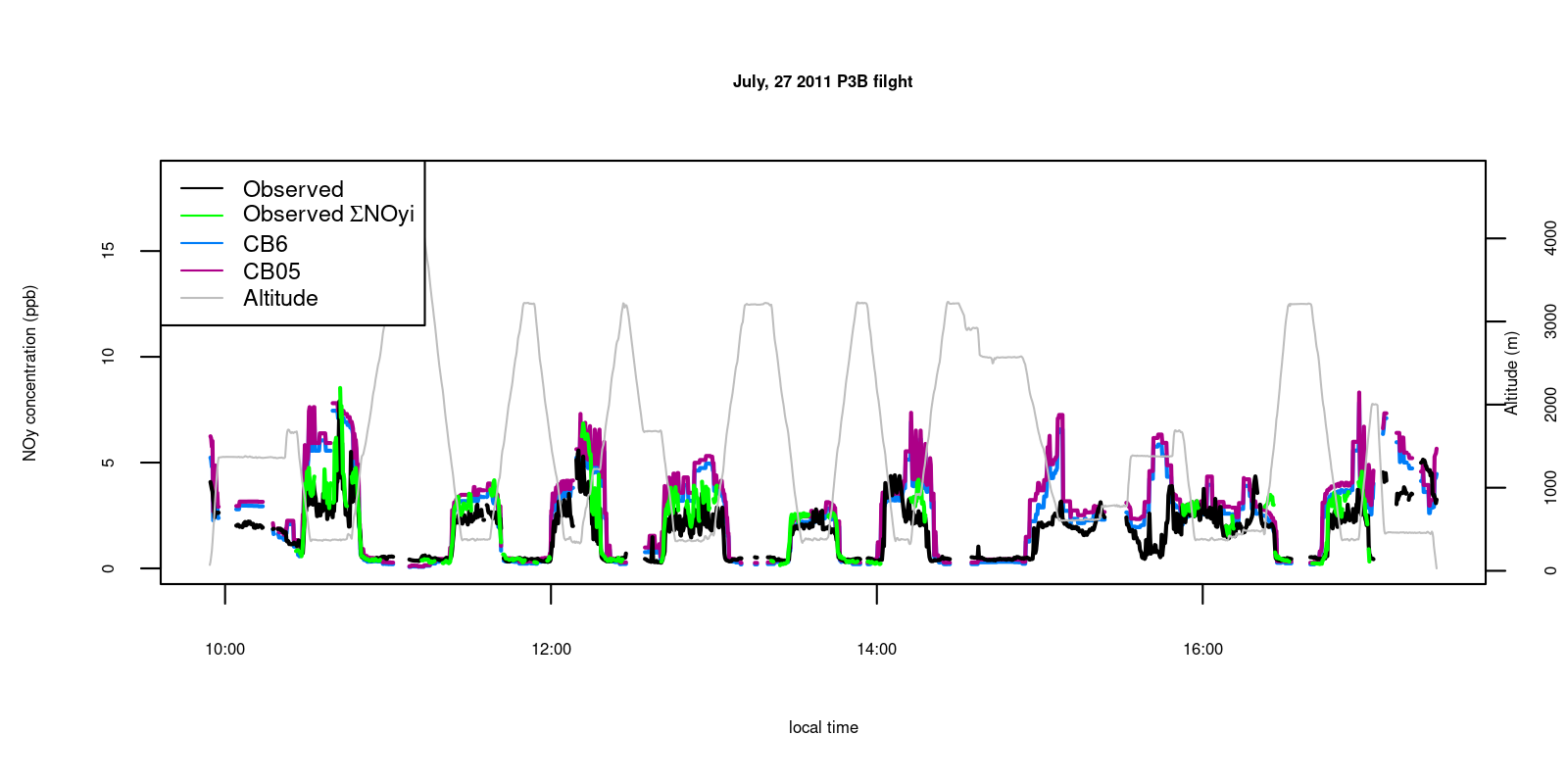


**Figure S32. Paired model and observed NOy from DISCOVER-AQ Baltimore flight on July 27, 2011.**


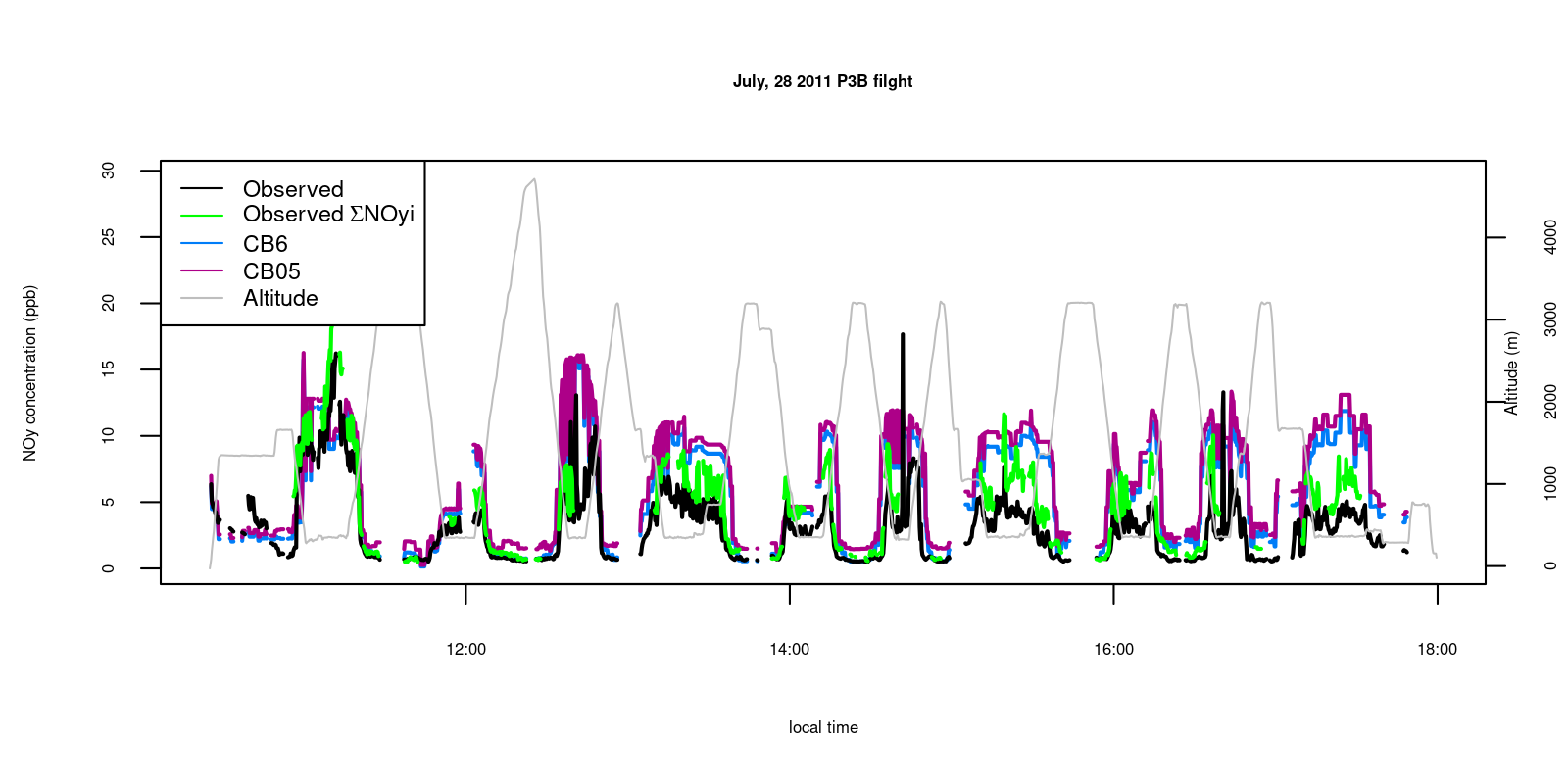


**Figure S33. Paired model and observed NOy from DISCOVER-AQ Baltimore flight on July 28, 2011.**


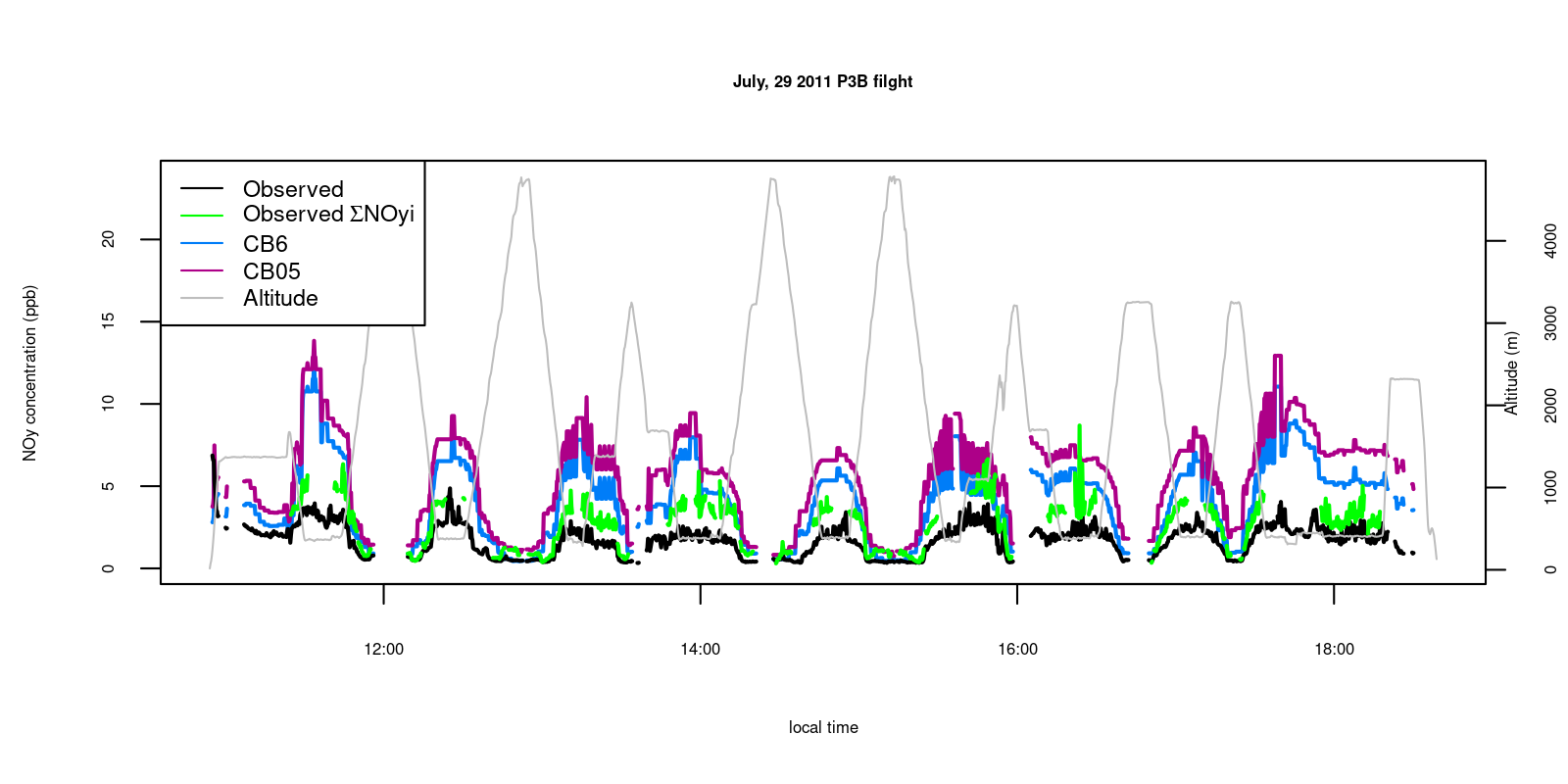


**Figure S34. Paired model and observed NOy from DISCOVER-AQ Baltimore flight on July 29, 2011.**

**Tables S1: List of model chemical mechanism species that were assigned to each measured NOy species.**

| Measured Species | NOx | ANs | PNs | NOy; ∑NOy_i_ |
| --- | --- | --- | --- | --- |
| Modeled species: CB05 | NO + NO2 | NTR | PAN + PANX + OPAN | NO + NO2 + NO3 + 2×N2O5 + HONO + HNO3 + PAN + PANX + PNA + OPAN + NTR + CRON + CRNO + CRN2 + CRPX + ANO3I* + ANO3J* + ANO3K* |
| Modeled species: CB6 | NO + NO2 | NTR1 + NTR2 + INTR | PAN + PANX + OPAN | NO + NO2 + NO3 + 2×N2O5 + HONO + HNO3 + PAN + PNA X+ PNA + OPAN + NTR1 + NTR2 + INTR + CRON + CLNO2 + ANO3I* + ANO3J* + ANO3K* |

*aerosol-phase species converted from µg/m^3^ to ppb for this calculation

## References Supplementary Material

Anderson DC, Loughner CP, Diskin G, Weinheimer A, Canty TP, Salawitch RJ, Worden HM, Fried A, Mikoviny T, Wisthaler A, et al. 2014. Measured and modeled CO and NOy in DISCOVER-AQ: An evaluation of emissions and chemistry over the eastern US. *Atmos Environ* **96**: 78–87. Elsevier Ltd. doi: 10.1016/j.atmosenv.2014.07.004

Federal Highway Administration. 2011. Vehicle Travel Information System (VTRIS). Available at https://fhwaapps.fhwa.dot.gov/vtris-wp/

Kang D, Foley KM, Mathur R, Roselle SJ, Pickering KE, Allen DJ. 2019. Simulating lightning NO production in CMAQv5.2: performance evaluations. *Geosci Model Dev* **12**(10): 4409–4424. Copernicus Publications. doi: 10.5194/gmd-12-4409-2019

Lee HJ, Chatfield RB, Bell ML. 2018. Spatial analysis of concentrations of multiple air pollutants using NASA DISCOVER-AQ aircraft measurements: Implications for exposure assessment. *Environ Res* **160**: 487–498. doi: https://doi.org/10.1016/j.envres.2017.10.017

Reed AJ, Thompson AM, Kollonige DE, Martins DK, Tzortziou MA, Herman JR, Berkoff TA, Abuhassan NK, Cede A. 2015. Effects of local meteorology and aerosols on ozone and nitrogen dioxide retrievals from OMI and pandora spectrometers in Maryland, USA during DISCOVER-AQ 2011. *J Atmos Chem* **72**(3): 455–482. doi: 10.1007/s10874-013-9254-9

Simon H, Valin LC, Baker KR, Henderson BH, Crawford JH, Pusede SE, Kelly JT, Foley KM, Owen RC, Cohen RC, et al. 2018. Characterizing CO and NOy Sources and Relative Ambient Ratios in the Baltimore Area Using Ambient Measurements and Source Attribution Modeling. *J Geophys Res Atmos* **123**: 3304–3320. doi: 10.1002/2017JD027688

U.S. Environmental Protection Agency. 2016. Technical Support Document (TSD): Preparation of Emissions Inventories for the Version 6.3, 2011 Emissions Modeling Platform. Available at https://www.epa.gov/sites/production/files/2016-09/documents/2011v6_3_2017_emismod_tsd_aug2016_final.pdf.

Zhang Y, Wang Y, Chen G, Smeltzer C, Crawford J, Olson J, Szykman J, Weinheimer AJ, Knapp DJ, Montzka DD, et al. 2016. Large vertical gradient of reactive nitrogen oxides in the boundary layer: Modeling analysis of DISCOVER-AQ 2011 observations. *J Geophys Res Atmos* **121**(4): 1922–1934. John Wiley & Sons, Ltd. doi: 10.1002/2015JD024203
